# Imbalanced gut microbiota fuels hepatocellular carcinoma development by shaping the hepatic inflammatory microenvironment

**DOI:** 10.1038/s41467-022-31312-5

**Published:** 2022-07-08

**Authors:** Kai Markus Schneider, Antje Mohs, Wenfang Gui, Eric J. C. Galvez, Lena Susanna Candels, Lisa Hoenicke, Uthayakumar Muthukumarasamy, Christian H. Holland, Carsten Elfers, Konrad Kilic, Carolin Victoria Schneider, Robert Schierwagen, Pavel Strnad, Theresa H. Wirtz, Hanns-Ulrich Marschall, Eicke Latz, Benjamin Lelouvier, Julio Saez-Rodriguez, Willem de Vos, Till Strowig, Jonel Trebicka, Christian Trautwein

**Affiliations:** 1 Department of Medicine III, University Hospital RWTH Aachen, Aachen, Germany; 2 Department of Microbiology, Perelman School of Medicine, University of Pennsylvania, Philadelphia, PA 19104 USA; 3 Helmholtz Centre for Infection Research, Braunschweig, Germany and Hannover Medical School, Hannover, Germany; 4 Institute for Computational Biomedicine, Bioquant, Heidelberg University, Faculty of Medicine, and Heidelberg University Hospital, Heidelberg, Germany; 5 Joint Research Centre for Computational Biomedicine (JRC-COMBINE), RWTH Aachen University, Faculty of Medicine, Aachen, Germany; 6 The Institute for Translational Medicine and Therapeutics, Perelman School of Medicine, University of Pennsylvania, Philadelphia, PA 19104 USA; 7 European Foundation for the Study of Chronic Liver Failure (EF-CLIF), 08021 Barcelona, Spain; 8 Translational Hepatology, Department of Internal Medicine I, Goethe University Frankfurt, 60323 Frankfurt, Germany; 9 Department of Molecular and Clinical Medicine/Wallenberg Laboratory, Sahlgrenska Academy, University of Gothenburg, Gothenburg, Sweden; 10 Institute of Innate Immunity, Medical Faculty, University of Bonn, 53127 Bonn, Germany; 11 Department of Infectious Diseases and Immunology, University of Massachusetts Medical School, Worcester, MA 01655 USA; 12 German Center for Neurodegenerative Diseases, 53127 Bonn, Germany; 13 Vaiomer SAS, Labège, France; 14 Laboratory of Microbiology, Wageningen University, 6708 WE Wageningen, The Netherlands; 15 Human Microbiome Research Program, Faculty of Medicine, University of Helsinki, P.O. Box 63, 00014 Helsinki, Finland

**Keywords:** Hepatocellular carcinoma, Microbiome, Tumour immunology

## Abstract

Hepatocellular carcinoma (HCC) is a leading cause of cancer-related deaths worldwide, and therapeutic options for advanced HCC are limited. Here, we observe that intestinal dysbiosis affects antitumor immune surveillance and drives liver disease progression towards cancer. Dysbiotic microbiota, as seen in *Nlrp6*^*−/−*^ mice, induces a Toll-like receptor 4 dependent expansion of hepatic monocytic myeloid-derived suppressor cells (mMDSC) and suppression of T-cell abundance. This phenotype is transmissible via fecal microbiota transfer and reversible upon antibiotic treatment, pointing to the high plasticity of the tumor microenvironment. While loss of *Akkermansia muciniphila* correlates with mMDSC abundance, its reintroduction restores intestinal barrier function and strongly reduces liver inflammation and fibrosis. Cirrhosis patients display increased bacterial abundance in hepatic tissue, which induces pronounced transcriptional changes, including activation of fibro-inflammatory pathways as well as circuits mediating cancer immunosuppression. This study demonstrates that gut microbiota closely shapes the hepatic inflammatory microenvironment opening approaches for cancer prevention and therapy.

## Abstract (web summary)

Steatohepatitis is a chronic hepatic inflammation associated with increased risk of hepatocellular carcinoma progression. Here the authors show that intestinal dysbiosis in mice lacking the inflammasome sensor molecule NLRP6 aggravates steatohepatitis and accelerates liver cancer progression, a process that can be delayed by antibiotic treatment.

## Introduction

Hepatocellular carcinoma (HCC) is the third leading cause of cancer-related deaths worldwide and the dominant cause of death in patients with compensated liver cirrhosis^1,2^. HCC incidence keeps rising and despite recent advances, therapeutic options remain limited^3^. HCC frequently arises in the context of chronic liver diseases (CLDs), with viral hepatitis B and C as well as alcoholic and non-alcoholic steatohepatitis (NASH) being the most common causes^4^. These conditions are characterized by chronic hepatic inflammation and continuous liver damage leading to hepatocyte cell death, which prompts compensatory proliferation and precedes hepatocarcinogenesis^5^. Therefore, a better understanding of these inflammatory processes is critical for developing new therapeutic strategies.

The NF-kB pathway is a core-signaling hub in hepatocytes that integrates the activity of various stress-related and inflammatory mediators^6^. NF-kB is a transcription factor that can translocate to the nucleus and initiate gene transcription. Two different pathways can trigger its activation: the canonical pathway via cytokines such as TNFα, IL-1β, or TLR agonists and the noncanonical pathway, which is mainly important in B cells^6^. The canonical pathway is mediated by phosphorylation of IκB by a high-molecular kinase complex, which is formed by two different catalytic IκB kinase 1 (IKK1 or alpha) and IKK2 (also IKKbeta) subunits as well as its regulatory subunit IκB kinase (IKK) subunit NF-kB essential modulator (NEMO or IKKγ). We and others have shown that blocking NF-kB activity in hepatocytes reduces inflammatory gene expression^7^. However, this process at the same time results in impaired expression of anti-apoptotic genes promoting overshooting cell death and compensatory proliferation^8^. Hence, conditional ablation of NEMO triggers spontaneous steatohepatitis and hepatocarcinogenesis in 12-months-old *NEMO*^*∆hepa*^ mice^8,9^. These mice are a well-established model to study steatohepatitis progression towards HCC^8^. In previous work, we have shown that activation of the canonical NF-kB pathway by experimental stimuli such as lipopolysaccharide (LPS) exacerbates this phenotype^10^.

The liver receives 2/3 of its blood supply through the portal vein. Hence, the liver is continuously exposed to a vast amount of pathogen- and microbe-associated molecular patterns (PAMPs and MAMPs), which bind to pathogen recognition receptors (PRRs), trigger NF-kB activation in hepatocytes and non-parenchymal cells, thereby driving liver disease progression^11^. This may critically shape the hepatic inflammatory microenvironment and fuel HCC development in the absence of NEMO.

In the diseased liver, senescence surveillance of pre-malignant hepatocytes by T cells limits liver cancer development by mounting specific immune responses^12^. Based on their high expression of PRRs, Ly6C^hi^CD11b^+^F4/80^low^ monocytic-derived suppressor cells (MDSCs) are well equipped to sense MAMPs and expand upon PRR activation^13^. Importantly, these cells can suppress the CD8^+^ cytotoxic T cell response and thus limit anti-tumor immunity^14^.

Mice lacking the inflammasome sensor molecule NLRP6 develop a dysbiotic colitogenic microbiota composition when housed under specific pathogen-free(SPF) conditions^15,16^. It has been shown that intestinal dysbiosis in mice deficient for NLRP6 promotes steatohepatitis via Toll-like receptor 4 (TLR4) and TLR9, a phenotype transmissible to co-housed wild-type (WT) mice^17^. Conversely, we and others have shown that microbiota depletion using broad-spectrum antibiotics dampens experimental steatohepatitis^17,18^. In recent clinical landmark studies, the presence of the gut microbiota and distinct bacteria were essential for an efficient anti-tumor immunotherapy^19–21^. Studies have linked the presence of the bacterium *Akkermansia muciniphila* to favorable treatment response to immunotherapy in several solid malignancies, including HCC^20,21^. Interestingly, murine studies show that oral supplementation with *A. muciniphila* reduces pro-inflammatory bacterial (LPS) and improves alcoholic liver disease^22,23^.

While the link between intestinal dysbiosis and liver disease progression is well established, mechanisms by which gut microbiota and bacterial translocation shape the hepatic inflammatory milieu and affect anti-tumor immune surveillance remain incompletely understood^24^. In human liver disease, intestinal dysbiosis is associated with intestinal barrier impairment, reduced microbiota diversity, overgrowth of certain unfavorable bacteria, and absence of beneficial communities^25^. Since *Nlrp6*^−*/−*^ mice mimic these hallmarks of intestinal dysbiosis, we used these mice as a tool to investigate how intestinal dysbiosis orchestrates the tumor microenvironment and affects the anti-tumor response during steatohepatitis progression^26^. We hypothesized that *Nlrp6*^−*/−*^-mediated intestinal dysbiosis aggravates steatohepatitis and increases tumor burden in *NEMO*^*∆hepa*^ mice. In this work, we demonstrate that intestinal barrier impairment and bacterial translocation dynamically induce expansion of mMDSCs and suppression of CD8^+^ T cells, which can be blocked by antibiotic treatment and reversed by the targeted reintroduction of the commensal *A. muciniphila*. Similarly, in a cohort of cirrhosis patients, we observe a strong association between bacterial translocation and activation of fibro-inflammatory pathways that mediate cancer immunosuppression highlighting the close functional interaction between gut and liver during liver disease progression.

## Results

### Absence of NLRP6 augments liver disease progression in *NEMO*^*∆hepa*^*/Nlrp6*^−*/−*^ mice

**Fig. 1:**
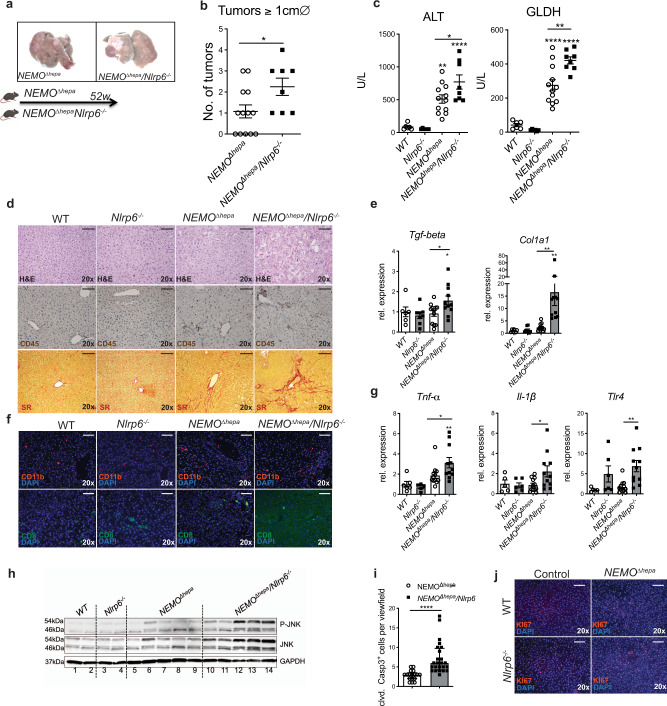
Loss NLRP6 augments liver disease progression in NEMO^∆hepa^ mice. **a** Macroscopic appearance of *NEMO*^*∆hepa*^ and *NEMO*^*∆hepa*^*/Nlrp6*^−*/−*^ livers at 52 weeks of age. **b** Quantification of liver tumors with a diameter larger than 1 cm in *NEMO*^*∆hepa*^ (*n* = 13) and *NEMO*^*∆hepa*^*/Nlrp6*^−*/−*^ (*n* = 8) livers; unpaired two-tailed Student’s *t* test, 95% CI −2.239 to −0.107, *p* = 0.0328. **c** Serum ALT and GLDH levels of 52-week-old *NEMO*^*∆hepa*^ (*n* = 12)*, NEMO*^*∆hepa*^*/Nlrp6*^−*/−*^ (*n* = 8)) and respective controls (*WT* (*n* = 6)*, Nlrp6*^−*/−*^ (*n* = 6)); one-way ANOVA with Tukey’s multiple comparisons test (ALT: WT vs. *NEMO*^*∆hepa*^, 95% CI −708.8 to −141.2, *p* = 0.0018; WT vs. *NEMO*^*∆hepa*^*/Nlrp6*^−*/−*^, 95% CI −1023 to −410.1, *p* = <0.0001; *NEMO*^*∆hepa*^ vs. *NEMO*^*∆hepa*^*/Nlrp6*^−*/−*^, 95% CI −519.1 to −0.93, *p* = 0.0489, GLDH: WT vs. *NEMO*^*∆hepa*^, 95% CI −339.4 to −127.3, *p* < 0.0001; WT vs. *NEMO*^*∆hepa*^*/Nlrp6*^−*/−*^, 95% CI −494.2 to −265.1, *p* = <0.0001; *NEMO*^*∆hepa*^ vs. *NEMO*^*∆hepa*^*/Nlrp6*^−*/−*^, 95% CI −243.1 to −49.49, *p* = 0.049). **d** Representative pictures of H&E-stained liver sections, immunohistochemical (IHC) staining of CD45 and Sirius red stained liver sections of 52-week-old *WT, Nlrp6*^−*/−*^*, NEMO*^*∆hepa*^, and *NEMO*^*∆hepa*^*/Nlrp6*^−*/−*^ livers, representative of 2 independent experiments; Scale bar: 100 µm. **e** RT-qPCR analysis of pro-fibrotic mRNA expression (*TGFβ*, *Col1a1*) in whole liver of *NEMO*^*∆hepa*^ (*n* = 12)*, NEMO*^*∆hepa*^*/Nlrp6*^−*/−*^ (*n* = 11) and respective controls (*WT* (*n* = 6)*, Nlrp6*^−*/*−^ (*n* = 8)); one-way ANOVA with Sidak’s multiple comparisons test (*TGFb*: WT vs. *NEMO*^*∆hepa*^, 95% CI −0.661 to 0.8128, *p* = ns; *Nlrp6*^−*/−*^ vs. *NEMO*^*∆hepa*^*/Nlrp6*^−*/−*^,95% CI –1.418 to −0.0483, *p* = 0.0328; *NEMO*^*∆hepa*^ vs. *NEMO*^*∆hepa*^*/Nlrp6*^−*/−*^, 95% CI −1.252 to −0.0219, *p* = 0.0405; *Col1a1*: WT vs. *NEMO*^*∆hepa*^, 95% CI −13.08 to 10.38, *p* = *ns*; *Nlrp6*^−*/−*^ vs. *NEMO*^*∆hepa*^*/Nlrp6*^−*/−*^,95% CI –26.78 to −3.865, *p* = 0.006; *NEMO*^*∆hepa*^ vs. *NEMO*^*∆hepa*^*/Nlrp6*^−*/−*^, 95% CI −24.53 to −3.950, *p* = 0.004). **f** Representative pictures of immunofluorescence (IF) stainings of CD11b and CD8 of *NEMO*^*∆hepa*^*, NEMO*^*∆hepa*^*/Nlrp6*^−*/−*^ and respective controls (*WT, Nlrp6*^−^^*/−*^*)*. Nuclei were counterstained with DAPI, representative of 2 experiments; Scale bar: 100 µm. **g** RT-qPCR analysis of inflammatory mRNA expression (*TNFα, IL-1β,Tlr4*) in whole liver of *NEMO*^*∆hepa*^ (*n* = 12)*, NEMO*^*∆hepa*^*/Nlrp6*^−*/−*^ (*n* = 11) and respective controls (*WT* (*n* = 4)*, Nlrp6*^−*/−*^ (*n* = 5))*;* one-way ANOVA with Sidak’s multiple comparisons test (*Tnfa*: WT vs. *NEMO*^*∆hepa*^, 95% CI −2.37 to 0.6196, *p* = ns; *Nlrp6*^−*/−*^ vs. *NEMO*^*∆hepa*^*/Nlrp6*^−*/−*^, 95% CI –4.04 to −0.806, *p* = 0.0015; *NEMO*^*∆hepa*^ vs. *NEMO*^*∆hepa*^*/Nlrp6*^−*/−*^, 95% CI −2.505 to −0.006, *p* = 0.023; *IL-1β*: WT vs. *NEMO*^*∆hepa*^, 95% CI −1.45 to 1.72, *p* = ns; *Nlrp6*^−*/−*^ vs. *NEMO*^*∆hepa*^*/Nlrp6*^−*/−*^, 95% CI –2.90 to −0.1197, *p* = ns; *NEMO*^*∆hepa*^ vs. *NEMO*^*∆hepa*^*/Nlrp6*^−*/−*^, 95% CI −2.601 to −0.1138, *p* = 0.047; *Tlr4*: WT vs. *NEMO*^*∆hepa*^, 95% CI −5.62 to 4.156, *p* = *ns*; *Nlrp6*^−*/−*^ vs. *NEMO*^*∆hepa*^*/Nlrp6*^−*/−*^, 95% CI –6.61 to −2.71, *p* = ns; *NEMO*^*∆hepa*^ vs. *NEMO*^*∆hepa*^*/Nlrp6*^*−/−*^, 95% CI −8.989 to −1.323, *p* = 0.005). **h** Immunoblot analysis of liver protein extracts from 52-week-old mice of all indicated genotypes for JNK, p-JNK and GAPDH as loading control; representative of 2 experiment. **i** Histological quantification of Cleaved Caspase 3 positive cells per viewfield; *NEMO*^*∆hepa*^ (*n* = 17) and *NEMO*^*∆hepa*^*/Nlrp6*^−*/−*^ (*n* = 24) viewfields one-way ANOVA with Tukey’s multiple comparisons test (*NEMO*^*∆hepa*^ vs. *NEMO*^*∆hepa*^*/Nlrp6*^*−/−*^, 95% CI −8.141 to −3.410, *p* < 0.0001). **j** Representative pictures of IF staining of KI67. Nuclei were counterstained with DAPI, representative of 2 experiments; Scale bar: 100 µm. All Data are presented as the mean ± standard error of the mean (SEM). Experiments are considered significant at *p* < 0.05 (*), *p* < 0.01 (**), *p* < 0.001 (***) and *p* < 0.0001 (****). Individual data points represent biological replicates unless otherwise stated. Source data are provided as a Source data file.

The inflammasome sensor molecule NLRP6 has been identified as a key regulator of host–microbiota homeostasis of the intestine^16^. To study the impact of *Nlrp6*^−*/−*^-mediated intestinal dysbiosis on steatohepatitis progression, we crossed *NEMO*^*∆hepa*^ with *Nlrp6*^−*/−*^ mice. 52-week-old *NEMO*^*∆hepa*^*/Nlrp6*^−*/−*^ mice displayed a significantly higher tumor burden (Fig. 1a, b) and significantly increased liver-to-bodyweight ratio compared to *NEMO*^*∆hepa*^ mice (Supplementary Fig. 1a).

*NEMO*^*∆hepa*^*/Nlrp6*^−*/−*^ mice showed increased liver injury as indicated by higher serum levels of alanine aminotransferase (ALT) and glutamate dehydrogenase (GLDH), as well as alkaline phosphatase (AP) and bilirubin compared to *NEMO*^*∆hepa*^ mice (Fig. 1c, Supplementary Fig. 1b). In H&E-stained liver sections and immunohistochemistry staining of CD45, *NEMO*^*∆hepa*^ and *NEMO*^*∆hepa*^*/Nlrp6*^−*/−*^ mice showed pronounced immune cell infiltration (Fig. 1d). Liver fibrosis was evidenced by Sirius red (SR) staining as well as messenger RNA (mRNA) expression of the pro-fibrotic genes *Tgfβ* and *Collagen1a1* (Fig. 1d, e). Liver sections revealed that both *NEMO*^*∆hepa*^ and *NEMO*^*∆hepa*^*/Nlrp6*^−*/−*^ mice showed pronounced immune cell infiltration (Fig. 1d). However, the livers of *NEMO*^*∆hepa*^*/Nlrp6*^−*/−*^ mice contained significantly increased numbers of CD11b^+^ cells compared to *NEMO*^*∆hepa*^ mice (Fig. 1f, Supplementary Fig. 1c). In contrast, CD8^+^ T cells were reduced upon loss of NLRP6 (Fig. 1f). Notably, loss of NLRP6 in WT mice was not sufficient to cause liver injury, inflammation, or fibrosis (Fig. 1, Supplementary Fig. 1).

As previously shown, in the absence of NEMO, activation of the canonical NF-kB pathway results in hepatocyte cell death due to the loss of anti-apoptotic gene expression^8^. Therefore, we characterized how hepatic inflammation impacts cell death and compensatory proliferation in these mice. While mRNA expression of the pro-inflammatory cytokines *Tnfa, Il6, Il1β* as well as inflammasome components *Caspase-1* and *Nlrp3* remained unchanged in tumor tissue (Supplementary Fig. 1d), infiltration of myeloid CD11b^+^ was linked to higher gene expression levels of the pro-inflammatory genes *Tnfa*, *Tlr4, Il1β*, *Nlrp3 and Ccl5* in whole liver tissue without macroscopic tumors (Fig. 1g, Supplementary Fig. 1e). Previous reports suggest that absence of NLRP6 can result in overactivation of NLRP3^27^. Whereas *Nlrp3* expression was increased on the mRNA level, we could not confirm this on the protein level (Supplementary Fig. 1e, f). Moreover, *Nlrp3* gene expression was unchanged in the early 13 weeks’ time point, suggesting that overexpression of NLRP3 inflammasome in the absence of NLRP6 does not mediate the observed phenotype (Supplementary Fig. 1g). The inflammatory phenotype of 52-week-old mice was associated with pronounced pJNK activation in *NEMO*^*∆hepa*^*/Nlrp6*^−*/−*^ mice (Fig. 1h) correlating with increased apoptotic hepatocyte cell death shown by cleaved Caspase3 staining and compensatory proliferation, based on KI67 staining (Fig. 1i, j and Supplementary Fig. 1h).

Together, these data demonstrate that the absence of NLRP6 orchestrates the inflammatory response in the tumor microenvironment and drives liver disease progression towards fibrosis and cancer in *NEMO*^*∆hepa*^*/Nlrp6*^−*/−*^ mice.

### Loss of NLRP6 is associated with intestinal dysbiosis and barrier impairment in *NEMO*^*∆hepa*^*/Nlrp6*^−*/*−^ mice

WT and *Nlrp6*^−*/−*^ littermates from an initial heterozygous *Nlrp6*^*+/*−^ breeding pair were used and separated after weaning to create independent WT and *Nlrp6*^−*/−*^ lines, which were kept for at least 3 generations under specific pathogen-free (SPF) conditions to allow the development of the dysbiotic *Nlrp6*^−*/−*^ community. Subsequently, we employed male mice of these lines to investigate the impact of intestinal dysbiosis on steatohepatitis progression. Microbiota composition of 13-week- and 52-week-old *NEMO*^*∆hepa*^ and *NEMO*^*∆hepa*^*/Nlrp6*^−*/−*^ as well as WT and *Nlrp6*^−*/−*^ control mice was analyzed in cecal samples using 16S rRNA gene amplicon sequencing.

**Fig. 2:**
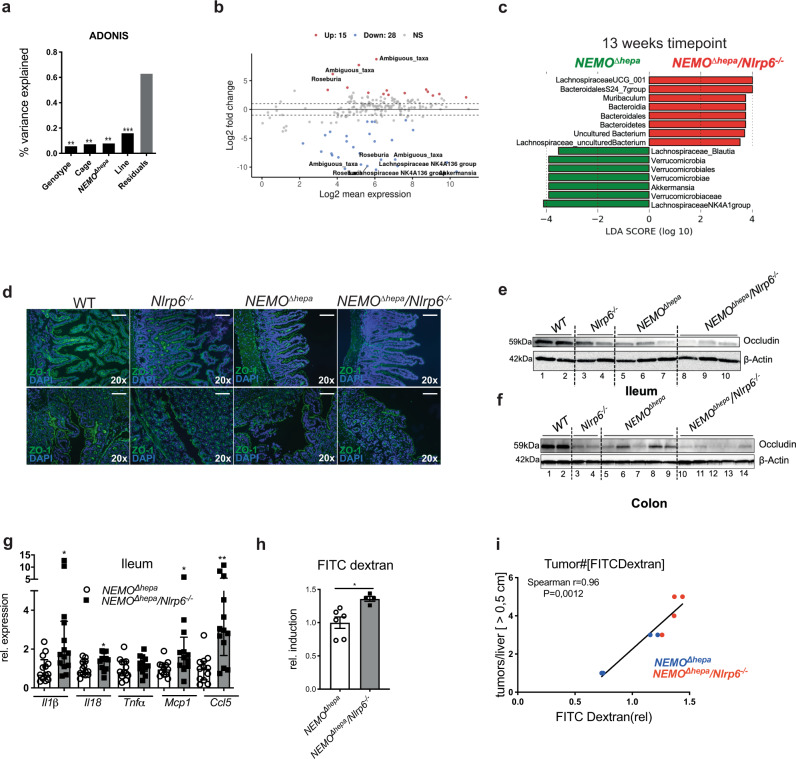
Loss of NLRP6 results in intestinal dysbiosis and barrier impairment correlating with steatohepatitis activity and tumor burden. **a** Cecal microbiota composition of 13-week-old mice (WT (*n* = 8), *Nlrp6*^−*/−*^ (*n* = 9), *NEMO*^*∆hepa*^ (*n* = 13), *NEMO*^*∆hepa*^*/Nlrp6*^−*/−*^ (*n* = 11)) was analyzed using 16S rRNA gene amplicon sequencing. Bar chart based on permutational multivariate analysis of variance (ADONIS) presenting the percentage of variance of the gut microbiota explained by the factors “Genotype” (*R*^2^ = 0.057, **p < 0.006), “Cage” (*R*^2^ = 0.073, **p < 0.004), “Nemo” (*R*^2^ = 0.079, **p < 0.002),” Line”. Genotype - all included genotypes; Cage – individual cages; Nemo – *WT* & *Nlrp6*^−*/−*^ mice vs. *NEMO*^*∆hepa*^ & *NEMO*^*∆hepa*^*/Nlrp6*^*−*^^*/−*^; Line – *WT* & *NEMO*^*∆hepa*^ vs. *Nlrp6*^−*/−*^ *& NEMO*^*∆hepa*^*/Nlrp6*^−*/−*^ (*R*^2^ = 0.16, ***p < 0.001). **b** Analysis for differential abundance of microbiota via DESeq analysis of 13 weeks old *NEMO*^*∆hepa*^ (*n* = 13) and *NEMO*^*∆hepa*^*/Nlrp6*^−*/−*^ (*n* = 11) mice. **c** Linear discriminant analysis (LDA) of effect size (LEfSe) between *NEMO*^*∆hepa*^ (*n* = 13) and *NEMO*^*∆hepa*^*/Nlrp6*^−*/−*^ (*n* = 11). **d** Representative pictures of IF stainings of ZO-1 in ileum and colon of *NEMO*^*∆hepa*^*, NEMO*^*∆hepa*^*/Nlrp6*^−*/−*^ and respective controls (*WT, Nlrp6*^−*/−*^*)*. Nuclei were counterstained with DAPI, representative of 2 independent experiments; Scale bar: 100 µm. **e**, **f** Immunoblot analysis of ileum and colon protein extracts from 52-week-old mice for Occludin and β-actin as loading control of indicated genotypes, representative of 2 experiments. **g** RT-qPCR analysis of inflammatory mRNA expression (*IL-1β, Il-18, TNFα, Mcp1, Ccl5*) in the ileum of *NEMO*^*∆hepa*^ (*n* = 12) *and NEMO*^*∆hepa*^*/Nlrp6*^−*/−*^ (*n* = 12) mice, unpaired *t*-test (*IL-1β*: *NEMO*^*∆hepa*^ vs. *NEMO*^*∆hepa*^*/Nlrp6*^−*/−*^, 95% CI 0.0235–2.421, *p* = 0.046; *Il-18*: *NEMO*^*∆hepa*^ vs. *NEMO*^*∆hepa*^*/Nlrp6*^−*/−*^, 95% CI 0.0293–0.7369, *p* = 0.035; *TNFα*: *NEMO*^*∆hepa*^ vs. *NEMO*^*∆hepa*^*/Nlrp6*^−*/−*^, 95% CI −0.210 to 0.595, *p* = n.s.; *Mcp1*: *NEMO*^*∆hepa*^ vs. *NEMO*^*∆hepa*^*/Nlrp6*^−*/−*^, 95% CI 0.097–1.932, *p* = 0.032; *Ccl5*: *NEMO*^*∆hepa*^ vs. *NEMO*^*∆hepa*^*/Nlrp6*^−*/−*^, 95% CI 1.023–4.706, *p* = 0.0038. **h** In vivo intestinal permeability assessed by oral gavage of FITC-dextran. Rel. induction of FITC-dextran permeability in *NEMO*^*∆hepa*^ (*n* = 6) and *NEMO*^*∆hepa*^*/Nlrp6*^−*/−*^ mice (*n* = 4), unpaired *t*-test: *NEMO*^*∆hepa*^ vs. *NEMO*^*∆hepa*^*/Nlrp6*^−*/−*^, 95% CI 0.101–0.615, *p* = 0.012*)*, pooled data from 2 independent experiments. **i** Strong correlation (Spearman) of intestinal barrier function evidenced by FITC-dextran permeability with tumor number in *NEMO*^*∆hepa*^ (blue dots) and *NEMO*^*∆hepa*^*/Nlrp6*^−*/−*^ (red dots) mice (8 pairs), *p* = 0.0012 (two-sided), *r* = 0.96. All Data are presented as the mean ± standard error of the mean (SEM; experiments are considered significant at *p* < 0.05 (*), *p* < 0.01 (**), *p* < 0.001 (***), and *p* < 0.0001 (****). Individual data points represent biological replicates unless otherwise stated. Source data are provided as a Source data file.

First, the beta-diversity, the difference in the gut microbiota composition between mice, was compared based on bray-Curtis dissimilarity among 13-week- and 52-week-old mouse groups. To evaluate the relative contribution of genotype (WT, *Nlrp6*^−^^*/−*^*, NEMO*^*∆hepa*^, *NEMO*^*∆hepa*^*/Nlrp6*^−*/−*^), cage, NEMO genotype (WT, *Nlrp6*^−*/−*^ vs. *NEMO*^*∆hepa*^, *NEMO*^*∆hepa*^*/Nlrp6*^−*/−*^) as well as mouse line (*Nlrp6*^−*/−*^*, NEMO*^*∆hepa*^*/Nlrp6*^−*/−*^ vs. WT, *NEMO*^*∆hepa*^) on the differences in microbiota composition between the groups, we performed permutational multivariate analysis of variance (ADONIS). Genotype, individual cage, *NEMO*^*∆hepa*^ genotype and mouse line explained a significant proportion of total microbiota variability (genotype *R*^2^ = 0.057, **p < 0.006; cage *R*^2^ = 0.073, **p < 0.004; *NEMO*^*∆hepa*^ genotype *R*^2^ = 0.079, **p < 0.002; mouse line *R*^2^ = 0.16, ***p < 0.001) in 13-week-old mice (Fig. 2a). As expected, these analyses revealed a significant cage effect. We, therefore, expanded our ADONIS analyses by including genotype and cage with strata option set to “cage.” Using this approach, genotype still explained a significant proportion of total microbiota variability (*R* = 0.296, *p* = 0.003).

Interestingly, although *NEMO*^*∆hepa*^ and *NEMO*^*∆hepa*^*/Nlrp6*^−*/−*^ were co-housed with their respective *NEMO*^*fl/fl*^ (referred to as WT) and *NEMO*^*fl/fl*^*/Nlrp6*^−*/−*^ littermate controls, conditional NEMO deficiency and the resulting steatohepatitis had a reproducible impact on microbiota composition. Importantly, beta-diversity analysis confirmed the development of a distinct microbiota in the *Nlrp6*^−*/−*^ line, which explained the highest proportion of 16% of total microbiota variability (Fig. 2a).

We performed differential abundance analysis based on the negative binomial distribution (DESeq2) and linear discriminant effect size analysis (LEfSe) to dissect which specific bacteria account for the observed differences. These analyses revealed a strong relative increase in *Muribaculum* in *NEMO*^*∆hepa*^*/Nlrp6*^−*/−*^ mice, while *Verrucomicrobiaceae* and several members of the family *Lachnospiraceae* were significantly reduced (Fig. 2b, c). Pairwise comparisons of all groups revealed a decrease of *Roseburia and Lachnospiraceae* in *Nlrp6*^−*/−*^ mice compared to WT mice. Interestingly, *Akkermansia* were increased in *NEMO*^*∆hepa*^ compared to WT littermates, which disappeared in *NEMO*^*∆hepa*^*/Nlrp6*^−*/−*^ (Supplementary Fig. 2a–c).

These bacteria were absent in *NEMO*^*∆hepa*^*/Nlrp6*^−*/−*^ both at the 13-weeks and 52-weeks’ time point, which discriminated *NEMO*^*∆hepa*^ from *NEMO*^*∆hepa*^*/Nlrp6*^−*/−*^ *in* LEfSe analyses (Fig. 2c, Supplementary Fig. 2d–f).

Next, we analyzed intestinal tissue sections of the different genotypes to explore the functional implications of the observed changes in microbiota composition. *A. muciniphila* is a well-known mucin-degrading bacterium. Its presence has been linked to thickening of mucus layers and intestinal barrier improvement^28^. Therefore, we analyzed colonic mucus layers. Loss of NLRP6 was associated with reduced thickness of colonic mucus layers, which was most pronounced in *NEMO*^*∆hepa*^*/Nlrp6*^−*/−*^ mice (Supplementary Fig. 2g).

Intestinal barrier function was studied in more detail by performing immunofluorescence staining for the tight-junction (TJ) protein zonula occludens1 (ZO-1) in different parts of the intestine. ZO-1 expression was reduced in duodenum, jejunum, ileum, and colon of 52-week-old *NEMO*^*∆hepa*^ mice compared to WT mice (Fig. 2d, Supplementary Fig. 2h). However, *NEMO*^*∆hepa*^*/Nlrp6*^−*/−*^ mice displayed a further reduction of ZO-1, which was most pronounced in the ileum and colon. In line, occludin protein expression was found to be reduced in ileum and colon tissue lysates of 52-week-old *NEMO*^*∆hepa*^*/Nlrp6*^−*/−*^ mice compared to *NEMO*^*∆hepa*^ mice (Fig. 2e, f). Interestingly, disruption of the TJ barrier was associated with increased mRNA expression of inflammatory genes such as *Il1β*, *Il18, Mcp1* and *Ccl5* and increased infiltration of CD11b^+^ cells in ileal tissue (Fig. 2g, Supplementary Fig. 2i).

Together, intestinal dysbiosis upon lack of NLRP6 expression prompted disruption of the intestinal TJ barrier and increased inflammatory gene expression.

### Intestinal permeability correlates with steatohepatitis activity and increased tumor burden

Next, we studied the functional implications of intestinal dysbiosis and intestinal barrier impairment in *NEMO*^*∆hepa*^*/Nlrp6*^−*/−*^ mice. Here, we evaluated in vivo intestinal barrier function in a cohort of 52-week-old *NEMO*^*∆hepa*^ and *NEMO*^*∆hepa*^*/Nlrp6*^−*/−*^ mice and respective controls. Permeability of the intestinal barrier measured by 4000 kDa FITC-dextran was significantly increased in *NEMO*^*∆hepa*^*/Nlrp6*^−*/−*^ compared to *NEMO*^*∆hepa*^ controls (Fig. 2h). Strikingly, ALT levels and tumor number demonstrated a strong correlation with intestinal permeability (Fig. 2i, Supplementary Fig. 2j), indicating that intestinal barrier impairment exacerbates liver disease in *NEMO*^*∆hepa*^ mice.

### Loss of NLRP6 shapes the hepatic immune environment

**Fig. 3:**
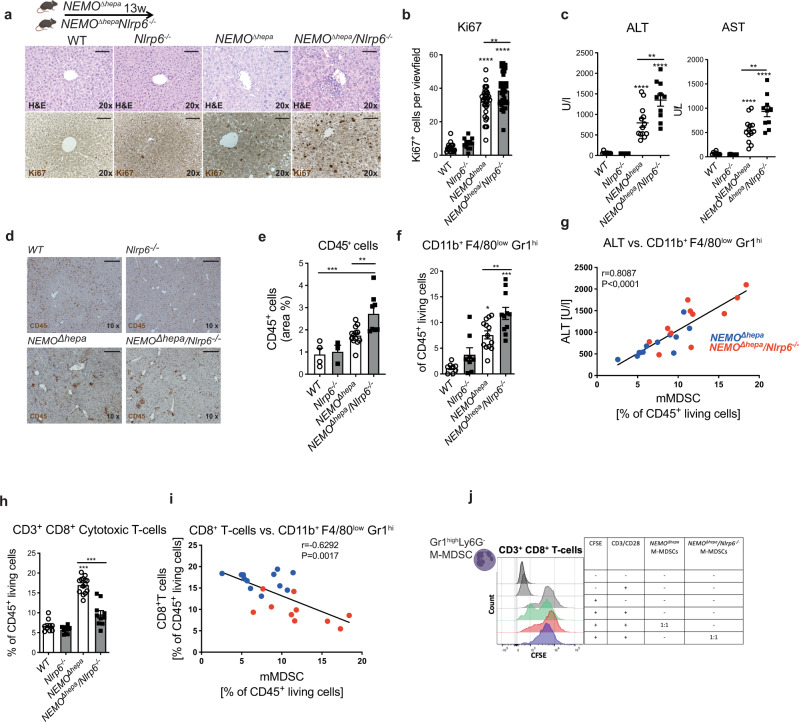
mMDSC drive steatohepatitis activity in *NEMO*^*∆hepa*^*/Nlrp6*^−*/−*^ mice. **a** Representative pictures of H&E-stained liver sections and immunohistochemical staining of KI67 stained liver sections of 13-week-old *WT, Nlrp6*^−*/−*^*, NEMO*^*∆hepa*^, and *NEMO*^*∆hepa*^*/Nlrp6*^−*/−*^ livers; Scale bar: 100 µm. **b** Histological quantification of Ki67^+^ cells per viewfield (*WT* *n* = 18, *Nlrp6*^−*/−*^ *n* = 12, *NEMO*^*∆hepa*^ *n* = 36, *NEMO*^*∆hepa*^*/Nlrp6*^−*/−*^ *n* = 35, one way ANOVA, Tukeys’s multiple comparison test:*Ki67*: WT vs. *NEMO*^*∆hepa*^, 95% CI −34 to −22, *p* < 0.0001; *WT* vs. *NEMO*^*∆hepa*^*/Nlrp6*^−*/−*^,95% CI –40 to −28, *p* < 0.0001; *NEMO*^*∆hepa*^ vs. *NEMO*^*∆hepa*^*/Nlrp6*^−*/−*^, 95% CI −11.54 to −1.67, *p* = 0.0039). **c** Serum alanine aminotransferase (ALT) and aspartate aminotransferase (AST) levels of 13-week-old *NEMO*^*∆hepa*^ (*n* = 13)*, NEMO*^*∆hepa*^*/Nlrp6*^−*/−*^ (*n* = 10) and respective controls (*WT* (*n* = 9)*, Nlrp6*^−*/−*^ (*n* = 8), one-way ANOVA, bonferroni’s multiple comparison test: ALT: WT vs. *NEMO*^*∆hepa*^, 95% CI −1.123 to −344.4, *p* < 0.0001; *WT* vs. *NEMO*^*∆hepa*^*/Nlrp6*^−*/−*^,95% CI –1700 to −875.6, *p* < 0.0001; *NEMO*^*∆hepa*^ vs. *NEMO*^*∆hepa*^*/Nlrp6*^−*/−*^, 95% CI −931.8 to −177.0, *p* = 0.002; AST: WT vs. *NEMO*^*∆hepa*^, 95% CI −764 to −210, *p* < 0.0001; *WT* vs. *NEMO*^*∆hepa*^*/Nlrp6*^−*/−*^, 95% CI –1147 to −588, *p* < 0.0001; *NEMO*^*∆hepa*^ vs. *NEMO*^*∆hepa*^*/Nlrp6*^−*/−*^, 95% CI −1.735 to −0.2338, *p* < 0.001). **d** Representative pictures and **e** quantification of immunohistochemical (IHC) staining of CD45 stained liver sections of 13-week-old mice (*WT*(*n* = 4)*, Nlrp6*^−*/−*^ (*n* = 3)*, NEMO*^*∆hepa*^ (*n* = 13)*,* and *NEMO*^*∆hepa*^*/Nlrp6*^−*/−*^ (*n* = 8), one way ANOVA, Tukey’s multiple comparisons test, *WT* vs. *NEMO*^*∆hepa*^*/Nlrp6*^−*/−*^,95% CI −2855 to −0.8097, *p* < 0.001; *NEMO*^*∆hepa*^ vs. *NEMO*^*∆hepa*^*/Nlrp6*^−*/−*^ 95% CI −7.5 to −0.8, *p* = 0.007); Scale bar: 200 µm. **f** Flow cytometry analysis of monocytic myeloid-derived suppressor cells (mMDSC) isolated from whole liver of 13-week-old *NEMO*^*∆hepa*^ (*n* = 12), *NEMO*^*∆hepa*^*/Nlrp6*^−*/−*^ (*n* = 11) and respective controls (*WT* (*n* = 7)*, Nlrp6*^−*/−*^ (*n* = 8) one-way ANOVA, Sidak’s multiple comparison test: WT vs. *NEMO*^*∆hepa*^, 95% CI −10.1 to −2.7, *p* = 0.0002; *Nlrp6*^−*/−*^ vs. *NEMO*^*∆hepa*^*/Nlrp6*^−*/−*^, 95% CI –11.8 to −4.2, *p* < 0.0001; *NEMO*^*∆hepa*^ vs. *NEMO*^*∆hepa*^*/Nlrp6*^−*/−*^, 95% CI −7.5 to −0.8, *p* = 0.009). **g** The percentage of mMDSCs of CD45^+^ living cells strongly correlates with ALT levels in *NEMO*^*∆hepa*^ and *NEMO*^*∆hepa*^*/Nlrp6*^*-/-*^ mice (Spearman, 23 pairs). **h** Flow cytometry analysis of CD3^+^CD8^+^ cytotoxic T cells isolated from whole liver of 13-week-old *NEMO*^*∆hepa*^ (*n* = 14), *NEMO*^*∆hepa*^*/Nlrp6*^−*/−*^ (*n* = 10) and respective controls (*WT* (*n* = 9)*, Nlrp6*^−*/−*^ (*n* = 9); one-way ANOVA: Sidak’s multiple comparison test: WT vs. *NEMO*^*∆hepa*^, 95% CI −12.4 to −8.0, *p* < 0.0001; *NEMO*^*∆hepa*^ vs. *NEMO*^*∆hepa*^*/Nlrp6*^−*/−*^, 95% CI 5.3 to −9.5, *p* < 0.0001. **i** Percentage of mMDSCs of CD45^+^ living cells and CD8^+^ cytotoxic T-cells is inversely correlated (Spearman 18 pairs). **j** In vitro T-cell proliferation assay. T cells were co-cultured with granulocytic MDSCs or monocytic MDSCs, representative of 2 experiments. All Data are presented as the mean ± standard error of the mean (SEM) Experiments are considered significant at *p* < 0.05 (*), p < 0.01 (**), *p* < 0.001 (***), and *p* < 0.0001 (****). Individual data points represent biological replicates unless otherwise stated. Source data are provided as a Source data file.

Next, we addressed how NLRP6 orchestrates the hepatic immune environment at an early stage of cancer development. 13-week-old *NEMO*^*∆hepa*^*/Nlrp6*^−*/−*^ mice displayed increased leukocyte infiltration, aggravated liver fibrosis evidenced by SR staining and increased hepatocyte proliferation supported by immunohistochemistry (IHC) staining for Ki67 (Fig. 3a, b, Supplementary Fig. 3a). More pronounced liver injury was reflected by significantly increased ALT, AST and GLDH levels in *NEMO*^*∆hepa*^*/Nlrp6*^−*/−*^ mice compared to *NEMO*^*∆hepa*^ mice (Fig. 3c, Supplementary Fig. 3c). In line, *NEMO*^*∆hepa*^*/Nlrp6*^−*/−*^ mice displayed a significantly increased liver-to-body weight ratio compared to *NEMO*^*∆hepa*^ mice (Supplementary Fig. 3d). Epithelial–mesenchymal transition during HCC development can be triggered by stellate cells. Indeed, we found increased stellate cell activation evidenced by aSMA staining in 13-week-old *NEMO*^*∆hepa*^*/Nlrp6*^−*/−*^ compared to *NEMO*^*∆hepa*^ mice (Supplementary Fig. 3e).

However, there was no evidence of increased hedgehog signaling activation in livers of *NEMO*^*∆hepa*^*/Nlrp6*^−*/−*^ mice (Supplementary Fig. 3f).

Intestinal dysbiosis and barrier impairment promote translocation of MAMPs via the portal vein into the liver, where they activate an innate immune response mediated by pathogen recognition receptors (PRRs). *NEMO*^*∆hepa*^*/Nlrp6*^−*/−*^ showed significantly increased infiltration of CD45^+^ leukocytes compared to *NEMO*^*∆hepa*^ livers (Fig. 3d, e). Flow cytometry analyses (FACS) (gating strategy—Supplementary Fig. 4a, b) of whole liver lysates revealed significantly increased infiltration of myeloid-derived suppressor cells (defined as CD45^+^CD11b^+^Ly6G^−^Gr1^hi^) as well as CD11b^+^Ly6G^+^ myeloid cells in *NEMO*^*∆hepa*^*/Nlrp6*^−*/−*^ mice compared to *NEMO*^*∆hepa*^ mice (Fig. 3f, Supplementary Fig. 4c). Strikingly, we found a strong correlation of mMDSC abundance with ALT levels suggesting their role as mediators of liver injury in *NEMO*^*∆hepa*^ mice (***p < 0.0001, *r* = 0.8087) (Fig. 3g).

Besides being well equipped with PRRs to trigger an inflammatory response upon exposure to MAMPs^29^, mMDSCs are defined by their suppressive capacity on T cells in the context of cancer development. Interestingly, *NEMO*^*∆hepa*^*/Nlrp6*^−*/−*^ mice displayed a strong reduction in cytotoxic CD8^+^ T cells (defined as CD45^+^CD3^+^CD8^+^) and CD4^+^ T cells (defined as CD45^+^CD3^+^CD4^+^) (Fig. 3h, Supplementary Fig. 4d). Consistent with suppression of T cells, mMDSC abundance was inversely correlated with cytotoxic T cells (**p = 0.0040, *r* = −0.5767) (Fig. 3i). Other immune myeloid and lymphoid immune cells subsets, including Kupffer cells, NK/NKT cells, and B cells remained unchanged (Supplementary Fig. 4e–h). We observed increased expression of the common M2 marker *Arg1*, while the M1 marker *Nos2* was decreased in *NEMO*^*∆hepa*^*/Nlrp6*^*−/−*^ mice, pointing towards a pro-tumorigenic microenvironment in these mice (Supplementary Fig. 4i).

### *NEMO*^*∆hepa*^*/Nlrp6*^−*/−*^ mMDSCs suppress T cell proliferation in vitro

MDSCs cannot be sufficiently defined based on cell surface marker expression^30^. Thus, we performed additional stainings and functional assays to further study the phenotype of these cells. CD11b^+^ mMDSCs and CD8^+^ T cells showed close proximity, which was most pronounced in livers of *NEMO*^*∆hepa*^*/Nlrp6*^−*/−*^ mice (Supplementary Fig. 5a). Next, we isolated and further characterized these cells and explored their inhibitory capacity on T cells by performing in vitro assays (Supplementary Fig. 5b). T cells were isolated from WT and *Nlrp6*^*−*^^*/−*^ spleens, labeled with CFSE, stimulated with CD3/CD28, and co-cultured with granulocytic MDSC (defined as CD45^+^Ly6G^+^Gr1^hi^) or mMDSCs (defined as CD45^+^Ly6G^−^Gr1^hi^) isolated from *NEMO*^*∆hepa*^ or *NEMO*^*∆hepa*^*/Nlrp6*^−*/−*^ livers by magnetic-activated cell sorting (MACS). We did not observe baseline differences in T cell proliferation between WT and *Nlrp6*^−*/−*^ mice (Supplementary Fig. 5c, d). Moreover, T cell proliferation remained unaffected upon co-culture with hepatic gMDSCs from either genotype (Supplementary Fig. 5c, d). However, mMDSCs strongly suppressed CD8^+^ T cell proliferation, most pronounced upon co-culture with mMDSCs isolated from *NEMO*^*∆hepa*^*/Nlrp6*^−*/−*^ livers (Fig. 3j).

Together, these data demonstrate that intestinal dysbiosis in *NEMO*^*∆hepa*^*/Nlrp6*^*−*^^*/−*^ mice is associated with an expansion of mMDSCs, which had a stronger suppressive capacity when isolated from *NEMO*^*∆hepa*^*/Nlrp6*^−*/−*^ than from *NEMO*^*∆hepa*^ mice.

### Microbiota depletion reshapes hepatic inflammatory microenvironment and ameliorates steatohepatitis

**Fig. 4:**
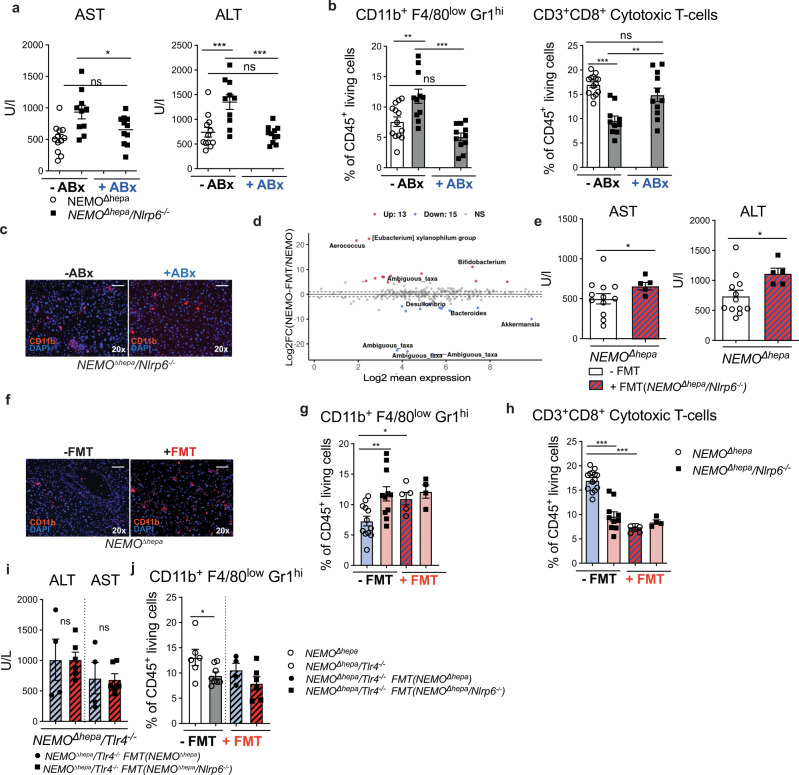
Microbiota modulation reshapes the hepatic inflammatory microenvironment in NEMO^∆hepa^ livers. **a** *NEMO*^*∆hepa*^ and *NEMO*^*∆hepa*^*/Nlrp6*^−*/−*^ mice were treated with broad-spectrum antibiotics (ABx) from week 8 till week 13. Serum aspartate aminotransferase (AST) and alanine aminotransferase (ALT) levels of *NEMO*^*∆hepa*^ (*n* = 12) *and NEMO*^*∆hepa*^*/Nlrp6*^−*/−*^ with (*n* = 11) or without (*n* = 10) 5 weeks ABx treatment, control groups (–Abx) shared with Fig.3c, Fig.4e; one way ANOVA, Sidak’s multiple comparison test: ALT: *NEMO*^*Δhepa*^ vs. *NEMO*^*Δhepa*^*/Nlrp6*^−*/−*^, 95% CI −980.6 to −240.7, *p* = 0.0007; *NEMO*^*Δhepa*^ vs*. NEMO*^*Δhepa*^*/Nlrp6*^−*/−*^ + ABx, 95% CI −316.0 to 405.4, *p* = n.s.; *NEMO*^*Δhepa*^*/Nlrp6*^−*/−*^ vs. *NEMO*^*Δhepa*^*/Nlrp6*^−*/−*^ + ABx, 95% CI 277.8 to 1033, *p* = 0.0004*;* AST: *NEMO*^*Δhepa*^ vs. *NEMO*^*Δhepa*^*/Nlrp6*^−*/−*^ + ABx, 95% CI 8.094 to 552.3, *p* = 0.047; *NEMO*^*Δhepa*^*/Nlrp6*^−*/−*^ vs. *NEMO*^*Δhepa*^*/Nlrp6*^−*/−*^ + ABx, 95% CI −399.3 to 120.6, *p* = n.s. **b** Flow cytometry analysis of monocytic myeloid-derived suppressor cells (mMDSC) and CD3^+^CD8^+^ cytotoxic T-cell isolated from whole liver of 13-week-old *NEMO*^*∆hepa*^ (*n* = 12–14) and *NEMO*^*∆hepa*^*/Nlrp6*^−*/−*^ with (*n* = 11) or without (*n* = 10–11) 5 wks Abx treatment control groups (–Abx) shared with Fig. 3d, f, one way ANOVA, Sidak’s multiple comparison test: CD11b: *NEMO*^*Δhepa*^ vs. *NEMO*^*Δhepa*^*/Nlrp6*^−*/−*^, 95% CI −7.624 to −1.307, *p* = 0.0036; *NEMO*^*Δhepa*^ vs*. NEMO*^*Δhepa*^*/Nlrp6*^−*/−*^ + ABx, 95% CI −0.9433 to 5.374, *p* = n.s*. NEMO*^*Δhepa*^*/Nlrp6*^−*/−*^ vs. *NEMO*^*Δhepa*^*/Nlrp6*^−*/−*^ + ABx, 95% CI 3454 to 9908, *p* < 0.0001*;* CD3+ CD8+: *NEMO*^*Δhepa*^ vs. *NEMO*^*Δhepa*^*/Nlrp6*^−*/−*^, 95% CI − 3.946 to 10.84, *p* < 0.0001; *NEMO*^*Δhepa*^ vs. NEMO^Δhepa^/NLRP6^−*/−*^+ ABx, 95% CI −1.245 to 5.460, *p* = n.s.; *NEMO*^*Δhepa*^*/NLRP6*^−*/−*^ vs. *NEMO*^*Δhepa*^*/Nlrp6*^−*/−*^ + ABx, 95% CI −8.919 to −1.648, *p* = 0.003*;* control groups (–Abx) shared with Fig. 3f, h and (**g**), (**h**). **c** Representative pictures of immunofluorescence staining of CD11b of *NEMO*^*∆hepa*^ and *NEMO*^*∆hepa*^*/Nlrp6*^−*/−*^ with and without Abx treatment. Nuclei were counterstained with DAPI, representative of 2 experiments; Scale bar: 100 µm. **d** Analysis for differential abundance of microbiota via DESeq analysis of 13 weeks old *NEMO*^*∆hepa*^ (*n* = 13) and *NEMO*^*∆hepa*^*-FMT* (*n* = 6) mice. *NEMO*^*∆hepa*^ group shared with Fig. 2. **e** Serum aspartate aminotransferase (AST) and alanine aminotransferase (ALT) levels of *NEMO*^*∆hepa*^ mice treated with *NEMO*^*∆hepa*^*/Nlrp6*^−*/−*^ (+FMT, *n* = 5) microbiota or without treatment (−FMT, *n* = 12), control group (–FMT) shared with Fig.3c, (**a**). **f** Representative pictures of immunofluorescence staining of CD11b of *NEMO*^*∆hepa*^ mice treated with +FMT or −FMT. Nuclei were counterstained with DAPI, representative of 2 experiments; Scale bar: 100 µm. **g** Flow cytometry analysis of monocytic myeloid derived suppressor cells (mMDSC) isolated from whole liver of *NEMO*^*∆hepa*^ mice treated with +FMT (*n* = 5) or –FMT (*n* = 12) and *NEMO*^*∆hepa*^*/Nlrp6*^−*/−*^ treated with +FMT (*n* = 4) or –FMT (*n* = 11), one way ANOVA, Holm–Sidak’s multiple comparison test: *NEMO*^*Δhepa*^ vs. *NEMO*^*Δhepa*^*/Nlrp6*^−*/−*^, *p* = 0.005; Nemo^Δhepa^ vs. Nemo^Δhepa^ +FMT, *p* = 0.040.; control group (–FMT) shared with Fig.4b, Fig. 3f. **h** Flow cytometry analysis of CD3^+^CD8^+^ cytotoxic T-cell isolated from whole liver of *NEMO*^*∆hepa*^ mice treated with +FMT (*n* = 5) or –FMT (*n* = 14) and *NEMO*^*∆hepa*^*/Nlrp6*^−*/−*^ treated with +FMT (*n* = 4) or –FMT (*n* = 10), one-way ANOVA, Sidak’s multiple comparison test: *NEMO*^*Δhepa*^ vs. *NEMO*^*Δhepa*^*/Nlrp6*^−*/−*^, 95% CI 4.296 to 10.49, *p* < 0.0001; Nemo^Δhepa^ vs. Nemo^Δhepa^ + FMT, 95% CI 4.226 to 11.68, p < 0.0001; control group (–FMT) shared with (**b**), Fig. 3h. **i** Serum alanine aminotransferase (ALT) and aspartate aminotransferase (AST) levels of *NEMO*^*∆hepa*^*/Tlr4*^−*/−*^ mice treated with *NEMO*^*∆hepa*^ (*n* = 4) or *NEMO*^*∆hepa*^*/Nlrp6*^−*/−*^ (*n* = 6) microbiota, unpaired *t*-test: AST: 95% CI −159.6 to 313.8, *p* = n.s., ALT: −711.9 to 713.5, *p* = n.s. **j** Flow cytometry analysis of mMDSC isolated from whole liver of *NEMO*^*∆hepa*^*/Tlr4*^−*/−*^ mice treated with *NEMO*^*∆hepa*^ (*n* = 4) or *NEMO*^*∆hepa*^*/Nlrp6*^−*/−*^ (*n* = 6) microbiota and respective controls (*NEMO*^*∆hepa*^ (*n* = 6) and *NEMO*^*∆hepa*^*/Tlr4*^−*/−*^ mice (*n* = 8) without treatment), unpaired *t*-test: *NEMO*^*Δhepa*^*/Tlr4*^−*/−*^ vs*. NEMO*^*Δhepa*^ 95% CI −7.038 to −0.09878, *p* = 0.045. All Data are presented as the mean ± standard error of the mean (SEM) and considered significant at *p* < 0.05 (*), *p* < 0.01 (**), p < 0.001 (***). Individual data points represent biological replicates unless otherwise stated. Source data are provided as a Source data file.

To test whether microbiota shapes the hepatic inflammatory response in *NEMO*^*∆hepa*^*/Nlrp6*^−*/−*^ livers, we treated 8-week-old mice until week 13 using an established combination of non-absorbable broad-spectrum antibiotics (ABx). As previously described^31^, this treatment led to an almost complete microbiota depletion evidenced by enlarged caeca similar to germ-free mice as well as significantly reduced total bacterial DNA content in fecal samples evidenced by qPCR assays using primers for all bacteria (Supplementary Fig. 6a). Strikingly, after ABx treatment liver transaminase levels of *NEMO*^*∆hepa*^*/Nlrp6*^−*/−*^ mice were similar to *NEMO*^*∆hepa*^ mice and significantly reduced compared to non-treated mice (Fig. 4a). Importantly, ABx treatment resulted in a significant reduction of mMDSC as evidenced by FACS and also reflected in a lower number of CD11b^+^ cells in IF staining (Fig. 4b, c). Conversely, ABx treatment resulted in an expansion of CD8^+^ T cells and CD4^+^ T cells, which almost reached the level as found in *NEMO*^*∆hepa*^ livers (Fig. 4b, Supplementary Fig. 6b).

### Immune phenotype of *NEMO*^*∆hepa*^*/Nlrp6*^−*/−*^ mice is transmissible to *NEMO*^*∆hepa*^ mice via FMT and involves TLR4 signaling

Microbiota depletion using ABx ameliorated liver injury, dampened mMDSC infiltration and restored cytotoxic T cell abundance in *NEMO*^*∆hepa*^*/Nlrp6*^−*/−*^ mice (Fig. 4b). To further test the pathogenic relevance of *Nlrp6*^−*/−*^ microbiota, we performed fecal microbiota transfer (FMT) of *NEMO*^*∆hepa*^*/Nlrp6*^−*/−*^ mice into *NEMO*^*∆hepa*^ mice. In accordance with successful FMT, *NEMO*^*∆hepa*^ receiving *NEMO*^*∆hepa*^*/Nlrp6*^−*/−*^ microbiota formed a distinct cluster that clustered close to *NEMO*^*∆hepa*^*/Nlrp6*^−*/−*^ FMT-Donors in NMDS ordination analysis based on Bray–Curtis dissimilarity (Supplementary Fig. 6c). Microbiota transfer resulted in a shift of *NEMO*^*∆hepa*^ microbiota along NMDS axis 1. DESeq2 as well as LEfSe analyses comparing *NEMO*^*∆hepa*^ mice with and without FMT revealed that this shift was mainly driven by differential abundance of *A. muciniphila* (Fig. 4d, Supplementary Fig. 6d). Strikingly, upon FMT all recipient *NEMO*^*∆hepa*^ mice were lacking *A. muciniphila* (Supplementary Fig. 6e), and only one differential OTU (OTU23_ambiguous_taxa) was observed in LEfSe analyses comparing *NEMO*^*∆hepa*^ FMT-recipient with *NEMO*^*∆hepa*^*/Nlrp6*^−*/−*^ FMT-Donors, further supporting successful microbiota transfer.

FMT resulted in a significant increase in liver transaminases AST and ALT in *NEMO*^*∆hepa*^ animals (Fig. 4e). Interestingly, FMT of *NEMO*^*∆hepa*^*/Nlrp6*^−*/−*^ microbiota prompted an increase in absolute numbers of CD45^+^ hepatic leukocytes, significant expansion of hepatic mMDSCs and suppression of cytotoxic T cells in recipient *NEMO*^*∆hepa*^ mice (Fig. 4f–h, Supplementary Fig. 6f).

Since intestinal dysbiosis in *NEMO*^*∆hepa*^*/Nlrp6*^−*/−*^ mice impairs intestinal barrier function and thus promotes activation of PRRs, we tested whether TLR4 is involved in mediating mMDSCs expansion upon FMT of *NEMO*^*∆hepa*^*/Nlrp6*^−*/−*^ microbiota. We generated *NEMO*^*∆hepa*^*/Tlr4*^−*/−*^ mice and transferred microbiota of *NEMO*^*∆hepa*^ or *NEMO*^*∆hepa*^*/Nlrp6*^−*/−*^microbiota into recipient littermates. Strikingly, in *NEMO*^*∆hepa*^*/Tlr4*^−*/−*^ the FMT of *NEMO*^*∆hepa*^*/Nlrp6*^−*/−*^ microbiota did not increase liver injury; serum AST and ALT levels and mMDSC abundance remained unchanged compared to control mice treated with *NEMO*^*∆hepa*^ microbiota (Fig. 4i, j).

Consistent with the involvement of TLR4 in mMDSC expansion, both 8- and 52–week-old *NEMO*^*∆hepa*^*/Tlr4*^−*/−*^ displayed a reduced abundance of these cells (Supplementary Fig. 6g). This observation was associated with significantly reduced liver transaminase levels (Supplementary Fig. 6h, i), reduced cell death as well as proliferation, and a markedly reduced tumor burden in 52-week-old mice (Supplementary Fig. 6j–l). Interestingly, reduced mMDSC abundance in *NEMO*^*∆hepa*^*/Tlr4*^−*/−*^ mice was associated with an increase in CD3^+^CD4^+^ T cells (Supplementary Fig. 6m).

To determine whether this immunologic phenotype was mediated by hematopoietic or parenchymal cells, we performed bone marrow transplantation experiments. Bone marrow chimeric *NEMO*^*∆hepa*^ mice receiving *Tlr4*^−*/−*^ donor bone marrow demonstrated a significant >6-fold reduction in mMDSC abundance compared to *NEMO*^*∆hepa*^ mice receiving WT control bone marrow pointing towards a role of TLR4 in hematopoietic cells for the observed phenotype (Supplementary Fig. 6n).

Together, these data demonstrate that the immune phenotype of *NEMO*^*∆hepa*^*/Nlrp6*^−*/−*^ mice is transmissible to *NEMO*^*∆hepa*^ mice upon FMT. Precisely, TLR4 signaling in hematopoietic cells augments mMDSC infiltration and promotes steatohepatitis progression towards HCC.

### Specific alterations of gut microbiota correlate with liver disease phenotype in *NEMO*^*∆hepa*^ mice

**Fig. 5:**
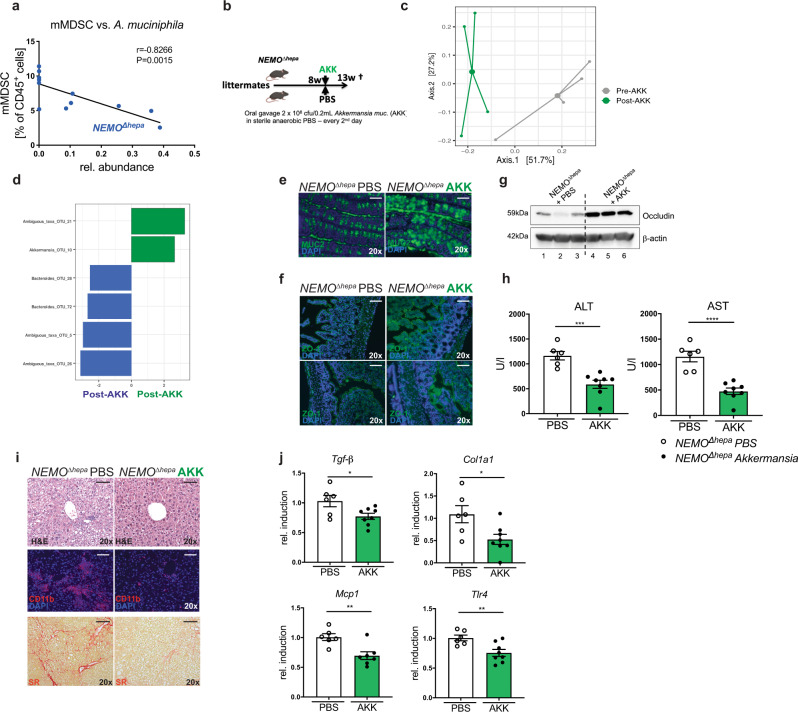
*Akkermansia muciniphila* supplementation ameliorates liver disease in *NEMO*^*∆hepa*^ mice. **a** Inverse correlation (Spearman, 13 pairs) between mMDSC and abundance of *A. muciniphila* (AKK) in caeca of *NEMO*^*∆hepa*^ mice. **b** Experimental setup: 8-week-old *NEMO*^*∆hepa*^ mice were gavaged either with 2.1 × 10^8^  CFU AKK or sterile anaerobic PBS for 5 weeks. **c** Principle Coordinates Analysis (PCoA) plot of microbiota (cecal content, stool) of *NEMO*^*∆hepa*^ mice (*n* = 4) before and after AKK treatment. **d** Results of LDA effect size (LEfSe) of cecal microbiota of *NEMO*^*∆hepa*^ mice (*n* = 4) before and after AKK treatment at bacterial taxa level. **e** Representative pictures of immunofluorescence staining of MUC2 in colon of *NEMO*^*∆hepa*^ mice treated with PBS or AKK. Nuclei were counterstained with DAPI, representative of 2 experiments; Scale bar: 100 µm. **f** Representative pictures of immunofluorescence staining of ZO-1 in ileum and colon of *NEMO*^*∆hepa*^ mice treated with PBS or AKK. Nuclei were counterstained with DAPI, representative of 2 experiments; Scale bar: 100 µm. **g** Immunoblot analysis of colon protein extracts from *NEMO*^*∆hepa*^ mice either treated with PBS or AKK for 5 weeks for occludin and β-actin as loading control, representative of 2 experiments. **h** Serum alanine aminotransferase (ALT) and aspartate aminotransferase (AST) levels of *NEMO*^*∆hepa*^ mice either treated with PBS (*n* = 6) or AKK (*n* = 8) for 5 weeks, unpaired *t*-test: ALT: *NEMO*^*Δhepa*^ AKK vs. *NEMO*^*Δhepa*^ PBS, 95% CI −835.1 to −312.4, *p* < 0.0001; AST: *NEMO*^*Δhepa*^ AKK vs. *NEMO*^*Δhepa*^ PBS, 95% CI −935.6 to −431.0, *p* < 0.0001. **i** Representative pictures of H&E-stained liver sections and immunofluorescence staining for CD11b in either PBS (*n* = 6) or AKK (*n* = 8) treated *NEMO*^*∆hepa*^ mice. Nuclei were counterstained with DAPI, representative of 2 experiments; Scale bar: 100 µm. **j** RT-qPCR analysis of inflammatory and fibrotic mRNA expression (*TGFβ, Col1a1, Mcp-1, Tlr4*) in whole liver of *NEMO*^*∆hepa*^ mice treated with PBS (*n* = 6) or AKK (*n* = 8) for 5 weeks, unpaired *t*-test, *TGFβ*: *NEMO*^*Δhepa*^ AKK (*n* = 8) vs. *NEMO*^*Δhepa*^ PBS (*n* = 6), 95% CI −0.4772 to −0.03676, *p* = 0.026; *Col1a1*: *NEMO*^*Δhepa*^ AKK (*n* = 8) vs. *NEMO*^*Δhepa*^ PBS (*n* = 6), 95% CI −1.023 to −0.1061, *p* = 0.019 *Mcp-1*: *NEMO*^*Δhepa*^ AKK (*n* = 7) vs. *NEMO*^*Δhepa*^ PBS (*n* = 6), 95% CI −0.5069 to −0.1172, *p* = 0.0048; *Tlr4*: *NEMO*^*Δhepa*^ AKK (*n* = 8) vs. *NEMO*^*Δhepa*^ PBS (*n* = 6), 95% CI −0.4238 to −0.07837, *p* = 0.008. All data are presented as the mean ± standard error of the mean (SEM) and considered significant at *p* < 0.05 (*), *p* < 0.01 (**), *p* < 0.001 (***), *p* < 0.001 (****), respectively. Individual data points represent biological replicates unless otherwise stated. Source data are provided as a Source data file.

Intestinal microbiota of *NEMO*^*∆hepa*^*/Nlrp6*^−*/−*^ was significantly different from *NEMO*^*∆hepa*^ mice after 13 and 52 weeks. Interestingly, microbiota modulation immediately reshaped the hepatic inflammatory microenvironment. In a final experiment, we, therefore, aimed to explore which specific changes in microbiota may modulate liver disease activity in *NEMO*^*∆hepa*^ mice. LEfSe analysis showed a major relative reduction in *A. muciniphila* in 13- and 52-week-old *NEMO*^*∆hepa*^*/Nlrp6*^−*/−*^ compared to *NEMO*^*∆hepa*^ mice. The bacterium *A. muciniphila* was significantly reduced in 13- and 52-week-old *NEMO*^*∆hepa*^*/Nlrp6*^−*/−*^ as well as *NEMO*^*∆hepa*^ mice receiving FMT (Fig. 2b, Supplementary Fig. 6d). Moreover, in *NEMO*^*∆hepa*^ microbiota relative abundance of *A. muciniphila* inversely correlated with hepatic mMDSC abundance (Spearman-*r* = 0.8508, *p* = 0.0005) as well as serum ALT and GLDH levels highlighting the relevance of these bacteria in mediating the observed phenotype (Fig. 5a, Supplementary Fig. 7a). Hence, we tested our hypothesis that the transfer of *A. muciniphila* ameliorates liver disease in *NEMO*^*∆hepa*^ mice. 8-week-old *NEMO*^*∆hepa*^ littermate mice were gavaged orally with 2*10^8^ colony forming units (CFUs) *A. muciniphila* or anaerobic PBS 3-times a week for 5 weeks (Fig. 5b). Successful microbiota transfer was confirmed by RT-qPCR and 16S rRNA gene amplicon sequencing of stool samples before and after 5 weeks of gavage (Fig. 5c, Supplementary Fig. 7b, c). Interestingly, AKK treatment did not only increase the abundance of this specific bacterium but resulted in a significant shift in microbiota composition as reflected in distinct clustering in principal coordinates analysis (PCoA) (Fig. 5c, Supplementary Fig. 7d). Treatment with *A. muciniphila* explained a large proportion of total microbiota variability observed in these mice (*R*^2^ = 0.403, *p < 0.05).

LEfSe analysis revealed a reduction of the phylum Bacteroides and expansion of *Akkermansia* (Fig. 5d). In DESeq2 analyses, *Akkermansia* supplementation also led to an increase in the abundance of *Lachospiraceae* and *Blautia*, which was associated with an increase in overall microbiota richness (Supplementary Fig. 7e). Increased abundance of the genus *A. muciniphila* in *NEMO*^*∆hepa*^ mice after transfer could also be confirmed using AldeX2 (Supplementary Table 1). In line with these data, we observed a significant expansion of the colonic mucus layers and increased ZO-1 expression in *NEMO*^*∆hepa*^ mice gavaged with *A. muciniphila* compared to PBS treated control mice (Fig. 5e, f, Supplementary Fig. 7f).

Consistent with these data, western blot analyses of colon tissue lysates revealed a significant and strong increase in occludin protein levels upon *A. muciniphila* administration (Fig. 5g, Supplementary Fig. 7g). Strikingly, serum levels of liver injury markers namely ALT, AST, LDH and GLDH were significantly decreased in *NEMO*^*∆hepa*^ mice gavaged with *A. muciniphila* compared to PBS treated controls (Fig. 5h, Supplementary Fig. 7h). This phenotype was reflected in histology: *A. muciniphila* treatment resulted in reduced liver fibrosis and significantly less leukocyte infiltration as evidenced by CD45 and CD11b staining (Fig. 5i, Supplementary Fig. 7i, j). In line, mRNA expression of fibro-inflammatory genes was significantly reduced *in A. muciniphila* treated *NEMO*^*∆hepa*^ mice (Fig. 5j).

Finally, we asked whether liver disease progression could even be improved in the *Nlrp6*^−*/−*^ dysbiosis model. *A. muciniphila* administration reduced liver injury in *NEMO*^*∆hepa*^*/Nlrp6*^−*/−*^ mice compared to PBS treated *NEMO*^*∆hepa*^ mice (Supplementary Fig. 8a, b), which was associated with significantly reduced infiltration of myeloid CD11b^+^ cells and lower liver fibrosis evidenced by Sirius red staining (Supplementary Fig. 8c, d). Together these data demonstrate that continuous *A. muciniphila* supplementation can reduce liver injury, inflammation, and fibrosis even in the presence of host-derived factors that promote dysbiosis such as NLRP6 deficiency.

### Bacterial translocation is higher in cirrhosis patients and shapes the hepatic transcriptomic landscape

We next tested if our observation in mice may also applies to humans. We therefore collected snap frozen liver tissue from patients with advanced liver cirrhosis of mixed etiology that underwent liver transplantation (*n* = 43) and controls undergoing other abdominal surgery (*n* = 12) (Supplementary Table 2). Small specimen from the same tissue region were cut and further processed for mRNA and isolation of bacterial DNA. Next, we analyzed hepatic bacterial DNA abundance using 16S rRNA gene amplicon sequencing in a strictly controlled environment using a stringent contamination-aware approach described and discussed previously^32,33^.

**Fig. 6:**
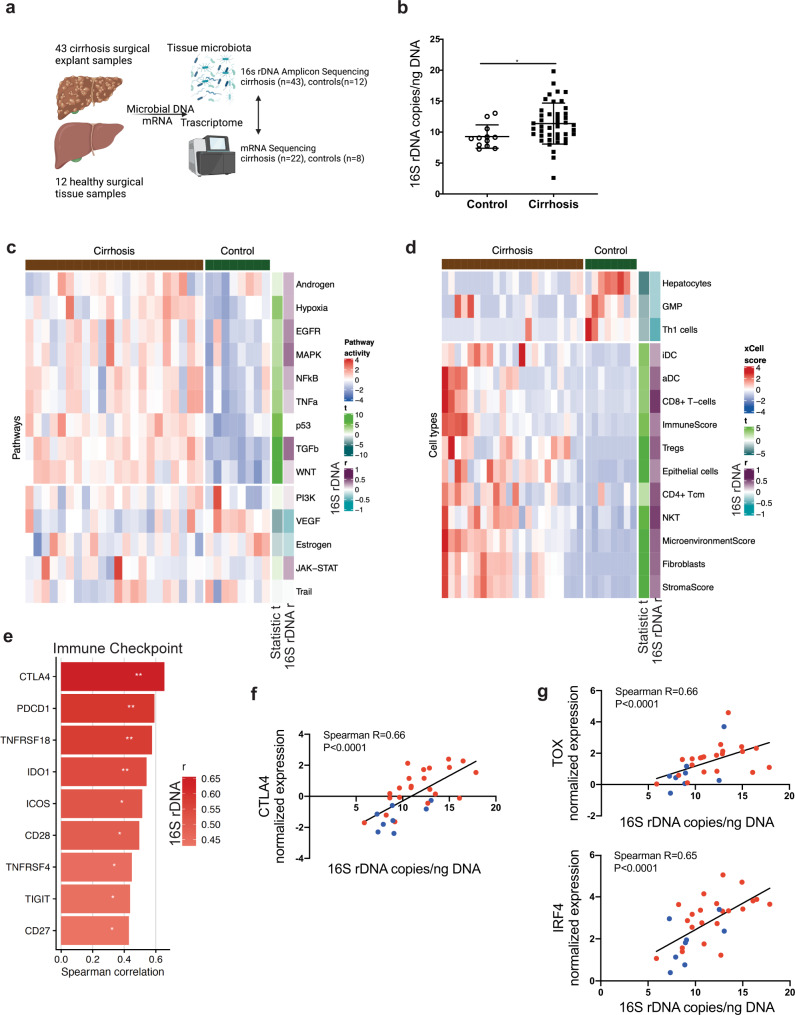
Hepatic bacterial 16s rDNA is increased in cirrhosis patients and shapes the hepatic transcriptomic landscape. **a** Study outline: Snap frozen surgical liver tissue specimen were taken from 44 cirrhosis patients that underwent liver transplantation or 11 healthy controls. DNA and mRNA were isolated from the same tissue specimen and tissue region and subjected to 16 s rRNA gene amplicon sequencing or mRNA sequencing. **b** 16s rRNA gene copies/ng DNA determined by real time quantitative PCR in control (*n* = 12) and cirrhotic (*n* = 43) liver (Mann–Whitney-*U*-Test, *p* = 0.014). **c** Pathway activity based on mRNA-sequencing data and inferred by PROGENy computational pathway analysis in cirrhosis patients (*n* = 22) vs. healthy controls (*n* = 8). Correlation of 16S rRNA gene abundance with pathway activation (Spearman correlation, *n* = 30 pairs). **d** Computational Cell type enrichment analysis and correlation of calculated cell types with 16S rRNA gene abundance of cirrhotic patients (*n* = 22) and healthy controls (*n* = 8). **e** 16S rRNA gene abundance strongly correlates with the expression of immune checkpoint genes (Spearman correlation, *n* = 30 pairs, two-tailed). **f** Correlation of *CTLA4* and **g** transcription factors involved in T-cell exhaustion (TOX, IRF4) with rRNA gene copies/ng genomic DNA (Spearman, *n* = 30 pairs, all 2-tailed, *p* < 0.0001). All Data are presented as the mean ± standard error of the mean (SEM) and considered significant at *p* < 0.05 (*), *p* < 0.01 (**), *p* < 0.001 (***). Source data are provided as a Source data file.

We also studied host gene expression by mRNA sequencing to assess how bacterial translocation affects the hepatic transcriptomic landscape (study outline see Fig. 6a).

Cirrhosis patients displayed significantly higher 16S rRNA gene copies per ng of total DNA compared to controls measured by RT-qPCR (Fig. 6b). Cirrhosis patients displayed higher alpha-diversity reflected in the Shannon index (Supplementary Fig. 9a). MDS ordination showed moderate clustering between the cirrhosis and control group (Supplementary Fig. 9b) (*R*^2^ = 0.038, *p* = 0.01, ADONIS) and *Stenotrophomonas, Roseburia, Sphingobiom* as well as *Psychrobacter* discriminated cirrhosis patients from controls in LEfSe analyses (Supplementary Fig. 9c). The order *Lactobacillales* was negatively correlated with patient’s MELD score and bilirubin levels (Supplementary Table 3).

PCA of transcriptomics data showed a distinct clustering of cirrhosis patients and controls (Supplementary Fig. 9d). Pathway analyses using the tool PROGENy^34,35^ identified fibro-inflammatory pathways TGFβ, NFκB, TNFα, and hypoxia and pathways associated with liver damage-repair, regeneration and malignant transformation such as EGFR, MAPK, P53, PI3K, and WNT to be significantly upregulated in cirrhosis patients compared to controls (Fig. 6c, Supplementary Table 3). Activation of MAPK (*r* = 0.366, *p* = 0.0464) and TGFβ (*r* = 0.383, *p* = 0.0367) signaling pathways correlated with 16S rDNA abundance in the liver (Fig. 6c). Moreover, *Clostridiales* abundance correlated with MAPK, EGFR, TNFa and NFκB pathway activation (Supplementary Table 4).

Several oncogenic transcription factors (TFs) inferred with DoRothEA^35^ including *FOS, ETS2, RELB, SOX10, ERG, WT1 and JUND* and TFs supporting stemness such as *PBPJ and ARID3A* were upregulated in cirrhosis patients and correlated with bacterial translocation (Supplementary Fig. 9e). Furthermore, cancer related genes correlated with 16 S rRNA gene abundance (Supplementary Fig. 9f).

### Bacterial translocation shapes the inflammatory microenvironment and promotes expression of T cell exhaustion markers in liver cirrhosis

Based on our murine data, we next specifically investigated the impact of bacterial translocation on the hepatic inflammatory milieu in human liver cirrhosis. To this end, we computationally dissected the hepatic cellular landscape based on gene expression profiles using xCell^36^. Cirrhotic livers showed enrichment for overall immune and stroma cells reflected in the microenvironment, stroma, and Immune xcell-scores, while the hepatocyte score was found to be relatively reduced (Fig. 6d). Interestingly, higher 16S rRNA gene abundance was associated with increased CD8^+^ T cells, NKT cells, central memory CD4^+^ and regulatory T cells, the latter of which have been implicated in tumor immunosuppression (Fig. 6d, Supplementary Table 5).

Several immune checkpoint genes including *PDCD1* encoding for PD-1 and cytotoxic T-lymphocyte-associated protein 4 (CTLA4) showed a strong correlation with 16S rRNA abundance (*PDCD1*: Spearman-*R* = 0.59, *P* = 0.0007; *CTLA4*: Spearman-*R* = 0.66, *P* < 0.0001, Fig. 6e, f). In line with these findings, gene expression and transcription factor activity of thymocyte selection associated high motility group box (*TOX*), interferon regulatory factor 4(*IRF4*) and *REL* and *BACH2*—which instruct T cell exhaustion and immune suppression in human cancer^37–40^—strongly correlated with 16S rRNA gene abundance (*TOX*: Spearman-*R* = 0.66, *P* < 0.0001; *IRF4:* Spearman *R* = 0.65, *P* < 0.0001, Fig. 6g, Supplementary Fig. 9e). Conversely, transcription factor expression and activity of *TOX3*—which has been associated with an activated T cell state^41^—was among the 5 TF with the strongest inverse correlation with 16S rDNA abundance (Supplementary Fig. 9g).

Activation of an innate immune response upon exposure with MAMPs and PAMPs depends on sensing via PRRs. Interestingly, the expression of several PRRs such as *NOD1*, *NLRP3, and TLR2* all correlated with bacterial translocation (Supplementary Fig. 9h). MDSCs are important sensors of MAMPs and PAMPs and may dampen T cell function. Expression levels of the MDSC markers colony-stimulating factor receptor2a (*CSFR2A*) and interleukin1-receptor2 (*IL1R2*) both demonstrated a strong correlation with 16S rRNA gene abundance (Supplementary Fig. 9i).

Together, these data show that bacterial translocation in cirrhotic patients is strongly associated with fibro-inflammatory pathways as well as TF activation linked to immunosuppression and T cell exhaustion.

## Discussion

In the last decade, the gut-liver axis and gut microbiota have emerged as cornerstones in the pathogenesis of chronic liver diseases^11,42–44^. Our present study defines intestinal barrier impairment and bacterial translocation as key mechanisms that shape the hepatic inflammatory microenvironment and fuel liver disease progression towards cirrhosis and HCC.

Chronic diseases, environmental and dietary factors associated with modern western lifestyles as well as medication have been found to contribute to intestinal dysbiosis. These factors trigger qualitative and quantitative changes in bacterial communities and directly affect the systemic inflammatory status^25,45^. As the liver is constantly exposed to a vast amount of microbiota-derived products from the gut via the portal vein, changes in intestinal homeostasis particularly impact liver physiology^11,46^.

Mouse models represent a suitable tool to functionally study mechanisms of gut-liver interaction allowing comprehensive mechanistic investigations. For our mechanistic studies, we decided to study chronic liver disease progression in *NEMO*^*∆hepa*^ mice lacking the inflammasome sensor molecule NLRP6, which has been identified as an important regulator of host-microbial crosstalk at the gut mucosal surface^15^. *NEMO*^*∆hepa*^ mice develop spontaneous steatohepatitis^8^, liver fibrosis and finally HCC. Interestingly, microbiota of *NEMO*^*∆hepa*^ was different from WT mice and these mice demonstrated reduced barrier function compared to WT littermate controls. The mechanisms by which hepatic loss of NEMO impairs in the intestinal barrier will be subject to future studies and could be mediated by changes in bile acid composition or low-grade systemic inflammation. Although the loss of NLRP6 NEMO other IKK components have not been described in human HCC, this mouse model nicely reflects essential mechanisms of human liver disease progression and allowed us to study the impact of intestinal dysbiosis induced by loss of NLRP6 on liver disease progression.

In our study, changes in gut microbiota composition of *NEMO*^*∆hepa*^/*Nlrp6*^−*/−*^ mice translated into impaired intestinal barrier function strongly correlating with markers of steatohepatitis activity as well as tumor burden. Interestingly, microbiota of *NEMO*^*∆hepa*^/*Nlrp6*^−*/−*^ mice was significantly different from *NEMO*^*∆hepa*^ mice. Specifically, *Nlrp6* deletion was associated with an increased abundance of the pathobiont *Muribaculum*, while *Akkermansia muciniphila* was absent. Several human, as well as murine studies, have demonstrated health benefits of *A. muciniphila* by promoting intestinal barrier function via regulation of intestinal mucus layers and acetate and propionate production^22,23,28,47^. Interestingly, loss of *A. muciniphila* has recently been described in patients with early HCC^48^.

To address whether the observed phenotype was caused by altered microbiota, we performed microbiota modulation experiments. Interestingly, the phenotype of *NEMO*^*∆hepa*^/*Nlrp6*^−*/−*^ mice was transmissible via FMT of the unfavorable *NEMO*^*∆hepa*^/*Nlrp6*^−*/−*^ community and reversible upon ABx treatment. NLRP6 deficiency in *NEMO*^*∆hepa*^ mice or transfer of *NEMO*^*∆hepa*^/*Nlrp6*^−*/−*^ microbiota transfer into *NEMO*^*∆hepa*^ mice triggered a pronounced infiltration of hepatic myeloid cells (defined as CD11b^+^Ly6G^+^Gr1^hi^). We termed these cells as mMDSCs based on surface marker expression and after confirming their in vitro suppressive capacity on T cell proliferation. These dynamic changes were linked to the reduced abundance of T cells pointing towards the high cellular plasticity of the hepatic inflammatory microenvironment related to microbiota. Several studies have highlighted the anti-tumor activity of CD8^+^ T cells in HCC^49,50^, however, this is dependent on their phenotype and tissue microenvironment^51^. They may also promote liver damage and progression towards HCC^52^. Future CD8^+^ T cell depletion experiments could help to establish causality in this model. HCC development Kupffer cells and macrophages may undergo phenotypic changes and promote a pro-tumorigenic microenvironment^53^. In our study we did not observe changes in Kupffer cell abundance, however, hepatic gene expression pointed towards an M2-skewed microenvironment. Regulatory T cells can be programmed in the gut and might exert their immunosuppressive function in the liver^54,55^. While not being the focus of our study, future studies on this mechanism might advance our understanding of gut-mediated immune modulation during HCC development.

Based on previous data and the landmark paper by Dapito et al., we hypothesized that PRR signaling and especially TLR4 may be an important orchestrator of this response^56^. Interestingly, *NEMO*^*∆hepa*^*/Tlr4*^−*/−*^ mice displayed a reduced abundance of MDSCs and an increase in CD4^+^ T cells, which was linked to a lower tumor burden at 52 weeks. Accordingly, transfer of the dysbiotic *NEMO*^*∆hepa*^/*Nlrp6*^−*/−*^ community failed to induce an expansion of mMDSCs in *NEMO*^*∆hepa*^*/Tlr4*^−*/−*^ mice. In line with previous data, these results clearly suggest an involvement of TLR4 mediated PRR signaling in MDSC expansion^57^. However, we cannot exclude that other PRRs, as well as other microbiota dependent pathways, may form equally important circuits directing the inflammatory response in the cirrhotic liver^58^.

Almost all cases of HCC arise in the context of cirrhosis, where chronic inflammation mediated by innate and adaptive immune responses drives disease progression^59^. However, immunosurveillance by T and B cells can also limit hepatocarcinogenesis^12^. In this context, the interplay between T cells and MDSCs is critical as MDSCs accumulation may induce T cell exhaustion promoting HCC progression^60,61^. Hence, immune-mediated mechanisms are essential during liver disease progression towards malignant transformation and dissecting their different functions will define novel therapeutic options. Gut microbiota can direct hepatic immunity in multiple ways via MAMPs, microbial metabolites, bile acids as well as short chain fatty acids^62,63^.

In patients, assessing the intestinal barrier as well as bacterial translocation and its molecular impact on the liver are challenging as there are no good non-invasive serum markers of barrier dysfunction and hepatic inflammation. While a series of studies have linked altered gut microbiota composition and metagenomic profiling to clinical and histopathological cirrhosis phenotypes^64,65^, data on whether these changes direct the hepatic inflammatory response or modulate the transcriptional landscape have not been available yet^24^. Based on our murine models, we hypothesized that bacterial translocation may also shape the hepatic inflammatory microenvironment in patients with advanced liver cirrhosis. We, therefore, assessed bacterial translocation by an established protocol of 16S rRNA analysis from liver tissue, quantified total bacterial DNA content, performed 16S rRNA gene amplicon sequencing and correlated these data with transcriptomic data generated from the same tissue specimen. In line with our murine data, bacterial translocation strongly correlated with fibro-inflammatory transcriptional pathways in human liver cirrhosis implicating bacterial translocation as a driver of liver disease progression.

A recent clinical study compared 20 Child Pugh A cirrhotic NAFLD patients with and without early HCC^48^. The authors found increased serum markers of intestinal inflammation (calprotectin) as well as permeability (ZO-1 and LPS) in cirrhosis patients vs. healthy controls. Interestingly, impaired barrier function was associated with reduced abundance of *Akkermansia* in NAFLD cirrhotic patients compared with controls and correlated with circulating mMDSCs in the HCC group.

Our functional data in the *NEMO*^*∆hepa*^ mouse model are in line with these findings. Moreover, in *NEMO*^*∆hepa*^ mice we observed profound changes of mMDSC and T cell abundance after short-term microbiota modulation. Additionally, supplementation with the single bacterium *Akkermansia muciniphila* improved intestinal barrier function, reduced infiltration of MDSCs and dampened steatohepatitis activity. Together, these data call for further studies to assess therapeutic supplementation with *Akkermansia* in HCC patients. The ideal study would involve the collection of liver biopsies as well as microbiota specimens, which would allow correlation of intestinal microbiota with hepatic 16S rRNA gene abundance and the hepatic transcriptional profile.

In a recent clinical trial daily supplementation of *A. muciniphila* for 3 months was well-tolerated, improved insulin sensitivity and blood lipid profiles in obese insulin-resistant individuals^66^. Similar to our murine data, modulating gut microbiota has the potential to reshape the hepatic inflammatory milieu in HCC patients, a hypothesis that is also inspired by a series of studies highlighting the role of microbiota in immune checkpoint therapy^20,67,68^. Here, recent studies have linked *Akkermansia* abundance to favorable treatment responses, while broad spectrum antibiotic intake before therapy which induces intestinal dysbiosis – impaired treatment responses^20^. While the strong immune-mediated pathogenesis highlights HCC as a particularly interesting target for immunotherapies, characteristics of the hepatic tumor microenvironment define a high barrier of resistance to immunotherapy^69,70^. Although the cirrhotic liver tissue they studied may not specifically reflect the HCC microenvironment, the observed correlations between hepatic 16S rRNA abundance and expression of fibro-inflammatory pathways, genes involved in cancer immunosuppression as well as MDSCs, T cell exhaustion, and PRR-signaling are likely also relevant in disease progression towards HCC. It is tempting to speculate that hepatic 16S rRNA gene abundance may serve as a biomarker of intestinal barrier impairment and dysbiosis that helps to predict treatment response to immune therapies and identify patients that could benefit from microbiota modulation. PCR-based measurements could be easily implemented in standard clinical biopsy workflows. A limitation of our study is that functional microbiota modulation studies were only performed in mice—additional studies in humans are needed. Based on our microbiota analyses as well as extensive literature on *A. muciniphila* and intestinal homeostasis, we focused our functional experiments on this commensal bacterium. In our study we observed a decrease in the abundance of *Blautia* in *NEMO*^*∆hepa*^/*Nlrp6*^*−/−*^ mice as well. Various recent publications have demonstrated anti-inflammatory probiotic properties of *Blautia* species due to production of short-chain fatty acids^71^. It is likely that *Blautia* or other commensal strains might be protective as well. Future studies could explore the role of *Blautia* in the context of HCC development. Finally, microbiota modulation experiments using germ-free mice would provide even more experimental precision. In these studies, it would be interesting to study whether the transfer of *Nlrp6*^−*/−*^ microbiota will eventually result in enhanced HCC development. Our current bulk RNA sequencing data clearly links bacterial translocation to fibro-inflammatory pathways as well as TF expression involved in T cell exhaustion. However, future studies including protein and histology data are warranted to substantiate these findings.

In summary, our data demonstrate that gut microbiota directly influence the hepatic inflammatory microenvironment in mice and men. An unfavorable microbiota—as seen in dysbiotic *NEMO*^*∆hepa*^*/Nlrp6*^−*/−*^ and transmissible to *NEMO*^*∆hepa*^ mice—fuel liver disease progression by promoting mMDSCs and dampening CD8^+^ T cells. Importantly, microbiota modulation immediately reshapes the inflammatory microenvironment providing a rational for microbiota targeted therapies. The strong association of liver tissue microbiota and hepatic transcriptomic profile in cirrhosis patients calls for larger studies to assess its diagnostic application.

## Methods

### Mice

*Male Alb-cre-NEMO*^*∆hepa*^*, Alb-cre-NEMO*^*fl/fl*^ *referred to as WT, Alb-cre-NEMO*^*∆hepa*^*/Nlrp6*^−*/−*^ and *Alb-cre-NEMO*^*∆hepa*^*/Tlr4*^−*/−*^ of the C57Bl6 background were bred and housed in the central animal facility of the University hospital RWTH Aachen. *NEMO*^*∆hepa*^*/Nlrp6*^−*/−*^ and *NEMO*^*∆hepa*^ lines were generated from an initial heterozygous breeding and then separated for at least 3 generations to allow the development of the *Nlrp6*^−*/−*^ dysbiotic microbiota community^25^. Subsequently, these two lines were kept strictly separate and we did not allow any exchange of mice or bedding material between the two lines as the microbiota related phenotype of these mice has been shown to be transmissible upon co-housing^17^.

All mice were housed in the individually ventilated cages with access to a standard chow diet and drinking water ad libitum. Upon birth, male mice were assigned to either no treatment, FMT or ABx groups and followed up until week 13. Experiments for these age progression experiments were run and analyzed in parallel. FMT or ABx was initiated in the respective groups at 7–9 weeks of age and continued until week 13. All mice were housed at a temperature of 21−23 °C with relative humidity of 35–65% and 12 h light/dark cycle. All animal experiments were approved by the appropriate German authorities (LANUV. North Rhine-Westphalia. (#AZ84-02.04.2013.A184 (C.T.), (#AZ84-02.04.2013.A260 (C.T.), #AZ84-02.04.2017.A 327 (C.T.), #AZ84-03.04.2013.A240 (C.T.)) All mice were treated in accordance to the criteria of the German administrative panels on laboratory animal care as outlined in the “Guide for the Care and Use of Laboratory Animals” prepared by the National Academy of Sciences and published by the National Institutes of Health (NIH publication 86-23 revised 1985).

### Cirrhosis cohort

Human cirrhosis liver tissue specimen were taken from patients that underwent liver transplantation between 1999 and 2005 at the University Hospital Bonn (Supplemental Table 2). The human ethics committee of the University of Bonn (029/13) approved the study. Healthy surgical tissue specimen were obtained from patients who underwent clinically indicated liver resection at University Hospital Bonn or University Hospital rechts der Isar of the Technical University Munich. All patients gave written informed consent to use excess biopsy material for research purposes. The study of these pseudonymized tissue specimen has been approved by the local ethics committee RWTH Aachen University (EK 196/19).

### Depletion of microbiota with broad spectrum antibiotics

For microbiota depletion, a broad-spectrum antibiotic cocktail (ampicillin 1 g/l, vancomycin 1 g/l, gentamycin 160 mg/l, metronidazole 1 g/l) was administered in the drinking water of 8-week-old *NEMO*^*∆hepa*^/*Nlrp6*^−/−^ mice. To decrease the bitter taste of the antibiotics, 25 g glucose were added per liter. Antibiotic treatment was performed until week 13. Antibiotic water was replenished every second day.

### Fecal microbiota transfer

For microbiota modulation experiments (fecal microbiota transfer, FMT), *NEMO*^*∆hepa*^ mice were treated for 5 weeks three times/week (Monday–Wednesday and Friday) via oral gavage with 200 µl of fecal dilution. To prepare this dilution, per mouse 20 mg of freshly harvested stool (immediately upon defecation) was collected from donor mice. Stool pellets were pooled and then vortexed for 5 min in 20 mg/100 µl anaerobic PBS to homogenize it almost entirely. Next, samples were gently centrifuged for 5 min at 350 × *g* to allow stool particulate to settle. The supernatant was collected and diluted 1:1 in anaerobic PBS. 200 µl of this suspension was transferred by oral gavage into recipient mice.

This is Akkermansia muciniphila MucT strain was isolated in the lab of Willem de Vos^28,66^. It was grown as detailed by Depommier et al. *Akkermansia muc*. was stored in Glycerol at a concentration of 2 × 10^8^ CFU/100 µl at −80 °C. Immediately before gavage *Akkermansia* was thawed and diluted 1:2 in anaerobic PBS reduced with 0.5 g/l of l-cysteine–HCl. Mice were then gavaged with either 200 µl of this solution or anaerobic PBS.

### Bone marrow transplantation

Bone marrow cells from WT and *Tlr4*^−*/−*^ donors were transplanted into 6-week-old WT, and *NEMO*^*Δhepa*^ recipients after ablative γ-irradiation. Recipients were radiated twice with 6 Gy with an interval of 4 h. Donors were sacrificed and femur and tibia were exposed. With a fine needle the medullary canal was flushed with Hanks/FCS. After twice washing with Hanks/FCS, cells were counted, and recipients received 1 × 10^6^ cells via tail vein injection after the second radiation. During the first four weeks mice received antibiotic water to minimize the danger of infection. Mice were sacrificed 8 weeks after transplantation.

### Intestinal permeability in vivo

Isothiocyanate conjugated dextran (FITC-dextran. molecular mass 4.0 kDa. Uppsala. Sweden) dissolved in PBS at a concentration of 200 mg/ml was administered to mice (10 ml/kg body weight) by oral gavage. 4 h after gavage the mice were sacrificed under general anesthesia by isoflurane. Blood samples were collected from inferior vena cava and immediately stored at 4 °C in in the dark. Concentration of FITC in serum was determined by spectrophotofluorometry at an excitation wavelength of 485 nm (20 nm band width) and an emission wavelength of 528 nm (20 nm band width). Relative induction of FITC signal relative to age-matched WT control mice was calculated.

### H&E—histology

Hematoxylin and eosin (H&E) staining was performed as previously described^18^. Briefly, tissue sections fixed in 4% paraformaledehyde (PFA) were cut into 2 µm sections. Tissue sections were deparaffinized and rehydrated. Next samples were stained with Mayer’s Hematoxylin solution for 1 min. Samples were rinsed in tap water for 15 min, placed in distilled water for 30 s, placed in 95% alcohol for 30 s and next counterstained in Eosin solution for 1 min. Finally, samples were dehydrated and mounted with coverslips using the the Roti^®^ Histokit.

### Sirius Red staining

Liver fibrosis development was studied using the following protocol. First, tissue sections embedded in paraffin were stained with Sirius red. For this purpose, tissue sections were deparaffinized by heating the slides at 65 °C for 15 min, followed by 2 × 5 min in xylene, and rehydration by introducing a descending concentration of ethanol (100% ethanol and 96% ethanol, 5 min in 70% ethanol and distilled water). Tissue sections were then placed for 45 min in a 0.1% Sirius red solution, followed by 2 × 15 s incubation in 0.5% glacial acetic acid. Finally, sections were dehydrated by ascending alcohol incubations (2 min 96%, 2 × 5 min 100% ethanol and 2 × 5 min xylene). Mounting of Tissue sections was performed with coverslips using the Roti^®^ Histokit.

### Immunohistochemistry staining

Five µm thick formalin-fixed, paraffin-embedded liver tissue sections were used to perform immunohistochemical stainings. First, the tissue sections were deparaffinized and rehydrated. For Antigen recovery, sections were heated in a pressure cooker in citrate buffer (pH 6.0). The tissue sections were then immersed in H_2_O_2_ solution (0.3% in methanol) for 10 min to block the endogenous peroxidases. To further block unspecific binding, the tissue sections were incubated in 1% bovine serum albumin in PBS for 2 h. Blocking was followed by incubation of the tissue sections overnight with the primary antibodies (Supplementary Table 6) at 4 °C in a humid chamber. After primary antibody incubations tisue sections were washed thoroughly in PBS. Next, the tissue sections were incubated with appropriate horseradish peroxidase-conjugated secondary antibodies (Supplementary Table 6) in a humid chamber at room temperature. Visualized of target signals was achieved by staining with 3,3′-diaminobenzidine solution (Vector Laboratories, Burlingame, CA, USA) for 2–5 min under the microscope. The nuclei were visualized by hematoxylin counterstaining. Finally, the stained sections were dehydrated in increasing concentrations of ethanol and mounted in Entellan.

### Immunofluorescence staining

After collection tissue specimens were immediately embedded in Tissue-Tek. Using a cryotome, tissues were cut into 5 µm-thick sections and stored at −80 °C. Slides were air-dried for 30 min at RT followed by 4% PFA fixation. Next, tissue samples were encircled using a hydrophobic pen and blocked with 5% goat serum for 1 h at RT in a humidity chamber.

After blocking, samples were incubated with the primary antibodies (Supplementary Table 6) at 4 °C in a humidity chamber overnight. Samples were washed thoroughly in PBS and then incubated with the secondary antibodies (Supplementary Table 6) for 1 h in a humidity chamber. After incubation, sections were washed again thoroughly in PBS. Finally, sections were mounted in a DAPI (Vector Laboratories, Burlingame, CA, USA) aqueous medium to counterstain nuclei. Staining of mucus and gut bacteria was performed according to an established protocol^72^. Briefly, colon tissue sections containing feces were fixed using the Carnoy fixation method (60% absolute methanol, 30% chloroform, 10% glacial acetic acid). After paraffin embedding, mucus and gut bacteria were stained with an anti-Muc2 primary antibody and a fluorescence in situ hybridization (FISH) probe against eubacteria (16S rRNA: 5′-GCTGCCTCCCGTAGGAGT-3′).

### Flow cytometry analysis of intrahepatic leukocytes

Same amounts of livers were digested by collagenase type IV for 1 h at 37 °C (Worthington Biochemical Corporation, Lakewood, NJ, USA) and intrahepatic immune cells were isolated by multiple differential centrifugation steps as detailed^73^. Cell isolates were incubated with blocking buffer for 30 min to block the unspecific binding sites of cell surface, then divided into two subgroups and stained with fluorochrome-conjugated antibodies either for myeloid cells FITC Rat anti-Mouse Ly-6G (561105; BD bioscience, Heidelberg, Germany), CD11b Monoclonal Antibody (M1/70), PE (12-0112-82, Thermo Fisher Scientific, Waltham, MA, USA), APC anti-mouse CD11c (117310, Biolegend, San Diego, CA, USA), F4/80 Monoclonal Antibody (BM8), PE-Cyanine7 (25-4801-82, Thermo Fisher Scientific, Waltham, MA, USA), PerCP-Cy™5.5 Rat Anti-Mouse Ly-6G and Ly-6C (552093, BD bioscience, Heidelberg, Germany), APC-Cy™7 Rat Anti-Mouse CD45 (557659, BD bioscience, Heidelberg, Germany) (1:200) or lymphocytes CD3e Monoclonal Antibody (145-2C11), APC (17-0031-83, Thermo Fisher Scientific, Waltham, MA, USA), CD4 Monoclonal Antibody (GK1.5), PE (12-0041-83, Thermo Fisher Scientific, Waltham, MA, USA) CD8a Monoclonal Antibody (53-6.7), FITC (11-0081-85, Thermo Fisher Scientific, Waltham, MA, USA), PerCP-Cy™5.5 Rat Anti-Mouse CD19 (551001, BD bioscience, Flow cytometry measurements were performed on a FACS Fortessa or FACS Canto instrument (BD, bioscience, Heidelberg, Germany). Data were analyzed with the FlowJo software (Ashland, OR, USA).

### DNA Isolation and 16S rRNA amplicon sequencing

For 16 S rRNA gene sequencing, DNA was isolated from fecal samples using an established protocol^74^. Briefly, each sample (around 200 mg) was resuspended in 500 µl of extraction buffer (200 mM Tris, 20 mM EDTA, 200 mM NaCl, pH 8.0). 200 µl of 20% SDS. 500 µl of phenol:chloroform:isoamyl alcohol (24:24:1) and 100 µl of zirconia/silica beads (0.1 mm diameter). Samples were homogenized twice with a bead beater (BioSpec, Bartlesville, OK, USA) for 2 min. After precipitation of DNA, crude DNA extracts were resuspended in TE Buffer with 100 µg/ml RNase I and column purified to remove PCR inhibitors.

Amplification of the V4 region (F515/R806) of the 16S rRNA gene was performed according to previously described protocols^75^. Briefly, for 16S rRNA amplicon sequencing 25 ng of DNA were used per PCR reaction (30 µl). The PCR conditions consisted of initial denaturation for 30 s at 98 °C, followed by 25 cycles (10 s at 98 °C, 20 s at 55 °C, and 20 s at 72 °C. Each sample was amplified in triplicates and subsequently pooled. After normalization PCR amplicons were sequenced on an Illumina MiSeq platform (PE250).

16S rRNA analysis was conducted based on a previously described computational workflow^76^. In brief, obtained reads were assembled, quality controlled and clustered using Usearch8.1 (http://www.drive5.com/usearch/). Next, reads were merged using -fastq_mergepairs –with fastq_maxdiffs 30 and quality controlled with fastq_filter (-fastq_maxee 1), minimum read length 200 bp. The OTU and representative sequences were determined using the UPARSE algorithm^77^, followed by taxonomy assignment using a curated Silva database v128^78^ and the RDP Classifier^79^ with a bootstrap confidence cutoff of 80%. The OTU absolute abundance table and mapping file were used for statistical analyses and data visualization in the R statistical programming environment (http://www.rproject.org) package phyloseq^80^. The permutational multivariate ANOVA (ADONIS test) was performed in R. Factors with *p* value < 0.05 were considered as significant. Differential abundance analysis (DAA) was performed using a consensus approach based on multiple methods (DESeq2, LefSE, and ALDEx2) to help ensure robust biological interpretation^81^. DESeq2 was performed using the parameters, test = “Wald”, fitType = “parametric”, alpha = 0.01)^82^. OTUs were considered significantly DA between genotypes if their adjusted p-value was <0.05 and if the estimated 2-fold change was >2 (Love et al., 2014, McMurdie and Holmes, 2014). LefSe was performed using the R wrapper *lefser* (Khleborodova A 2021) with the following parameters kruskal.threshold = “0.05”, wilcox.threshold = “0.05”, lda.threshold = “2.5”. ALDEX2^83^ Was performed using default settings, OTUs were considered significantly DA between contrasts if (*we.eBH* Expected Benjamini–Hochberg corrected *p* value of Welch’s *t* test) or (*wi.eBH* Expected Benjamini–Hochberg corrected *p* value of Wilcoxon test) was <0.05.

### 16S rDNA quantitation and taxonomic profiling in liver tissue

Microbial DNA was isolated from frozen liver biopsies with a protocol designed to minimize the risk of contamination between samples, by the environment or experimenters as previously described^32^. Negative controls consisting of molecular grade water were placed in separate isolation tubes during the isolation process and processed simultaneously throughout the protocol. DNA was amplified using real-time polymerase chain reaction (qPCR) amplification using universal 16S primers targeting the hypervariable V3–V4 region of the bacterial 16s ribosomal RNA gene. qPCR was performed on a ViiA 7^®^ PCR system (Life Technologies, Carlsbad, CA, USA) using Sybr Green technology. Quality control and quantification of the extracted nucleic acids were performed based on gel electrophoresis (1% w/w agarose in TBE 0.5x) and absorption spectroscopy with a NanoDrop 2000 UV spectrophotometer (Thermo Fisher Scientific, Waltham, MA, USA). High-throughput next-generation sequencing of microbial rDNA was performed using Illumina MiSeq technology as previously described^84^. Next, (a) The last 20 bases of reads R1 were removed; (b) the last 40 bases of reads R2 were removed; (c) amplicons <350 or >500 nucleotides in length were removed; (d) OTUs with a frequency <0.005% of the total record frequency have been removed; (e) Total Sum Scaling (TSS) normalization was used to normalize OTU read counts to relative frequencies. Because the number of sequences per sample was high and fairly constant between samples (Supplementary Fig. 10a), we chose not to rarefy the data in order to normalize the number of sequences in each sample.

Numerous controls both in vitro and in silico were included to ensure the absence of artifacts related to non-specific amplification of eukaryotic DNA or reagent contamination^33^. Negative controls and liver samples were compared based on qPCR and beta diversity analyses and showed a clear separation (Supplementary Fig. 10b,c).

In line with our previous data, these numerous quality controls demonstrate that potential bacterial contamination was well contained and had a negligible impact on the taxonomic profiles of the samples in our study^33,85,86^.

### qRT-PCR

Frozen tissue samples from liver or intestine were homogenized in 1 ml Trizol Reagent (Life Technologies, Carlsbad, CA, USA). 200 µl chloroform were added to separate the phases, the upper aqueous phase was transferred into a new collection tube. 500 µl isopropanol were added and the samples remained at RT for 15 min. Afterwards, the RNA was pelleted by centrifugation at 13,000 × *g* for 10 min at 4 °C, the supernatant was discarded, and the pellets were washed twice with ethanol 70%. Next pellets were air dried and 300 µl DEPC water was used for resuspension. For transcription 1 µg of the isolated mRNA were used and reverse transcription into cDNA was performed using Omniscript^®^ RT Kit (Cat. No. 205113. Qiagen, Venlo, The Netherlands) according to the manufacturer’s protocol. Real-time PCR reactions were performed with Real-Time PCR System Quant studio 6 Flex (Thermo Fisher Scientific, Waltham, MA, USA) and Fast SYBR^®^ GreenER Master Mix (Thermo Fisher Scientific, Waltham, MA, USA) according to manufacturer’s recommendations. The primers were diluted 1:10 fold or 1:50 respectively. All primer sequences are listed (Supplemental Table 7). The Quant Studio Flex software (Thermo Fisher Scientific, Waltham, MA, USA) was used for analysis. In the following the relative mRNA expression was calculated with the 2^−ΔΔ^CT method comparing target gene expression to the GAPDH house-keeping gene.

### Library preparation and mRNA sequencing

After quality control with the Agilent Tape Station 4200 RNA ScreenTape Analysis and quantification with the QuantiFluor RNA System (Promega), the library preparation was done according to the manufacturer’s protocol with the Illumina TruSeq Stranded Total RNA Library Prep Gold kit with IDT for Illumina—TruSeq RNA UD Indexes. Sequencing of the library pool was done on one lane using the Illumina NovaSeq 6000 S4 Reagent Kit (200 cycles) with the NovaSeq Xp 4-Lane Kit.

### mRNA sequencing analysis

#### Pre-processing and normalization of RNA-seq data

FASTq files were aligned against the reference genome using the web application BioJupies. The count data were normalized using the Bioconductor package edgeR (version 3.30.0) that filters for lowly expressed genes and corrects for differences in library composition^87^. Using the Bioconductor package limma (version 3.44.1) we transformed the normalized data to log2-counts per million^88^.

#### Transcription factor activity inference with DoRothEA

Transcription factor (TF) activity can be inferred from gene expression data by interrogating the expression of the respective transcriptional targets (i.e., its regulon). It has been shown that this approach is more robust and accurate than observing the expression of the TF itself. We used DoRothEA as the regulon resource as it contains signed TF-target interactions for the majority of all human (and mouse) TFs^35^. Internally DoRothEA uses the statistical method viper to access the TF activity from gene expression data and returns for each TF a normalized enrichment score (NES) that we consider a proxy for TF activity.

DoRothEA was applied to the normalized gene expression matrix with the following arguments: “method = ‘scale’”, “nes = T,” “minsize = 4” and “eset.filter = F”, using the Bioconductor package dorothea (version 1.0.0; https://saezlab.github.io/dorothea/).

Differences in TF activities between healthy and cirrhotic patients were computed with a *t*-test. To adjust *p*-values for multiple hypothesis testing we computed the false discovery rate (FDR).

#### Pathway activity inference with PROGENy

PROGENy is a tool that allows predicting pathway activities from gene expression data in human (and mouse)^34^. Instead of interrogating the expression of pathway members, PROGENy takes the expression of the most responsive genes of a pathway into account. These most responsive genes upon pathway perturbation are referred to as footprints (the concept of footprints is reviewed in ref. ^89^. With PROGENy it is possible to infer the activity of these 14 signaling pathways in human (and mouse): Androgen, EGFR, Estrogen, Hypoxia, JAK-STAT, MAPK, NFkB, PI3K, TGFb, TNFa, Trail, p53, VEGF and WNT.

We applied PROGENy to the normalized gene expression matrix with the following parameters “top = 100”, “perm = 1”, “scale = T”, using the Bioconductor package progeny (version 1.10.0; https://saezlab.github.io/progeny/).

Differences in pathway activities between healthy and cirrhotic patients were computed with a *t*-test. To adjust p-values for multiple hypothesis testing we computed the false discovery rate (FDR).

#### Cell types enrichment with xCell

xCell is a tool that performs sample-wise cell type enrichment from gene expression data^36^. We subsetted the collection of the original 64 immune and stromal cell types to cell types relevant for the liver and the studied phenotype (“iDC”, “ImmuneScore”, “CD8+ T-cells”, “Tregs”, “Epithelial cells”, “NKT”, “MicroenvironmentScore”, “Fibroblasts”, “StromaScore”, “Hepatocytes”, “Th1 cells”, “GMP”, “CD4+ Tcm”, “aDC”).

As suggested by the xCell vignette we transformed the raw counts of the gene expression data to transcripts per million (TPM). Afterward, xCell was applied to the TPM matrix using the R package xCell (version 1.1.0; https://github.com/dviraran/xCell).

Differences in cell type enrichment between healthy and cirrhotic patients were computed with a *t*-test. To adjust *p*-values for multiple hypothesis testing we computed the false discovery rate (FDR).

### Immunoblotting

The liver and intestine tissue samples were homogenized with NP-40 Buffer containing phosphatase inhibitor cocktail tables (cOmplete mini, PhosSTOP (Roche, Basel, Switzerland) for protein isolation. Protein concentrations were measured using BIO-RAD protein reagent, then adapted to 2 µg/µl, before the proteins were separated electrophoretically on pre-cast 4–12% polyacrylamide gel (Bio-Rad, Hercules, CA, USA) in SDS running buffer at 160 V. After running, the gel was immediately placed in buffer to transfer the proteins to the nitrocellulose blotting membrane with the Trans-Blot Turbo Transfer System (Bio-Rad, Hercules, CA, USA). The success of transfer was checked using Ponceau Red. Before incubating with primary antibodies, the membrane was blocked with 5% non-fat dry milk or 5% BSA diluted in TBS-Tween (TBST 0.5%) to prevent unspecific antibody binding. Subsequently, the membrane was incubated with primary antibodies diluted 1:1000 in 5% dry milk or BSA overnight at 4 °C under agitation. The horseradish peroxidase (HRP)-conjugated secondary antibodies were diluted 1:2000 in 5% dry milk and the membrane was incubated for 1 h at RT. ECL substrate (Pierce, Waltham, MA, USA) developing solution was used before image acquisition with the LAS mini 4000 developing machine (Fuji). Protein expression was quantitatively analyzed with ImageJ in relation to the expression of GAPDH. The following antibodies were used in this study: β-actin (A2066, Sigma-Aldrich, St. Louis, MO, USA), Occludin (71-1500, Thermo Fisher Scientific, Waltham, MA, USA 71-1500), p-JNK/p-SAPK (#9251S, Cell signaling, Danvers, MA, USA). JNK/SAPK (#9252S, Cell signaling, Danvers, MA, USA). GAPDH (AHP1628, Bio-Rad, Hercules, CA, USA).

### In-vitro MDSC assay

#### MDSC isolation

MDSCS were isolated with Myeloid-Derived Suppressor Cell Isolation Kit (mouse; 130-094-538, Miltenyi, Wuppertal, Germany) from liver. After preparing a single cell suspension, the cell number was determined. Cell suspension was centrifuged at 300 × *g* for 10 min at 4 °C. Supernatant was aspirated completely. Cell pellet was resuspended in 350 μl of buffer per 10^8^ total cells and 50 µl of FcR Blocking Reagent per 10^8^ total cells were added, mixed, and incubated for 10 min in the refrigerator (2−8 °C). 100 μl of Anti-Ly-6G-Biotin (MDSC-Kit) were added, mixed, and incubated for 10 min in the refrigerator (2−8 °C). Cells were washed by adding 5−10 ml of buffer per 10^8^ cells and centrifuged at 300 × *g* for 10 min at 4 °C. Supernatant was aspirated completely and up to 10^8^ cells were resuspended in 800 μl of buffer. 200 μl of Anti-Biotin MicroBeads were added, mixed, and incubated for 15 min in the refrigerator (2−8 °C). Cells were washed by adding 10−20 ml of buffer per 10^8^ cells and centrifuged at 300 × *g* for 10 min at 4 °C. Supernatant was aspirated completely and up to 10^8^ cells were resuspended in 500 μl of buffer. LS Column was placed in the magnetic field of a suitable MACS Separator. Column was rinsed with 3 ml of buffer and cell suspension applied onto the column. Flow-through was collected which contained the unlabeled cells. Column was washed with 3 × 3 ml of buffer. The unlabeled cells which passed through were combined with the effluent from step 3; These cells represented the unlabeled pre-enriched Gr-1^dim^Ly-6G^–^ cell fraction. Column was removed from separator and a collection tube was placed under. 5 ml of buffer was added onto the column and the magnetically labeled cells were flushed out by firmly pushing the plunger into the column. These cells represented the labeled Gr-1^high^Ly-6G^+^ cell fraction.

The unlabeled pre-enriched Gr-1^dim^Ly-6G^−^ cell fraction was centrifuged at 300 × *g* for 10 min at 4 °C. Supernatant was aspirated completely and up to 10^8^ cells were resuspended in 400 µl buffer. 100 µl of Anti-Gr-1-Biotin per 10^8^ cells was added, mixed, and incubated for 10 min at 4 °C. Per 10^8^ cells 5–10 ml of buffer were added and centrifuged at 300 × *g* for 10 min at 4 °C. Supernatant was aspirated completely and up to 10^8^ cells were resuspended in 900 μl of buffer. In addition, 100 µl of Streptavidin MicroBeads were added, mixed, and incubated for 15 min at 4 °C. 10–20 ml buffer per 10^8^ cells were added and centrifuged at 300 × *g* for 10 min at 4 °C. Supernatant was aspirated completely and up to 10^8^ cells were resuspended in 500 μl of buffer. MS columns were placed in the magnetic field and 500 µl of buffer were added onto the column. Cell suspension was applied onto the column and the collected and represented the unlabeled cells. The column was washed 3 × 500 µl. All flow through were collected. Column was removed from separator and a collection tube was placed under. 1 ml of buffer was added onto the column and the magnetically labeled cells were flushed out by firmly pushing the plunger into the column. These cells represented the labeled Gr-1^dim^Ly-6G^−^ cell fraction.

#### T cell isolation

T cells were isolated with (mouse; 130-095-130, Miltenyi, Wuppertal, Germany) from spleen. After preparing a single cell suspension, cell number was determined. Up to 10^7^ cells were resuspended in 40 µl buffer and 10 µl of biotin–antibody cocktail per 10^7^ total cells were added, mixed, and incubated for 5 min at 4 °C. 30 µl of buffer and 20 µl of Anti-Biotin MicroBeads per 10^7^ total cells were added, mixed, and incubated for 10 min at 4 °C. LS columns were placed in the magnetic field and 3 ml of buffer added onto the column. Cell suspension was applied onto the column and flow through collected. Column was washed 3 × 3 ml and flow through collected.

#### T cell CFSE labeling

T cells were centrifuged with 300 × *g* for 10 min at 4 °C and resuspended in 1 ml PBS/0.1% BSA. A solution of CFDA-SE (Vybrant CFDA SE Cell Tracer Kit, V12883, Thermo Fisher Scientific, Waltham, MA, USA) from DMSO Stock at 2X final labeling solution was prepared (100 µM). T cells were resuspended in 1 ml solution containing CFDA-SE dilution and incubated in the dark for 15 min at 37 °C. Cells were quenched with 4 ml ice cold T cell medium and centrifuged with 300 × *g* for 10 min at 4 °C. Cells were washed two times.

#### In vitro T cell assay

U bottom 96 wells were coated with 2 mg/ml CD3 antibody (6-0032-85 (Clone 17A2; 1 mg/ml) Thermo Fisher Scientific, Waltham, MA, USA) and incubated for 2 h at 37 °C. Plates were washed three times with PBS prior to the start of the assay. T cells (10^5^ cells) were incubated with gMDSCs or mMDSCs in the following ratios: 1:0, 1:1, 1:2 or 1:4. Additionally, 10 µg/µl of CD28 (553294 (Clone 27.51; 1 mg/ml) BD bioscience, Heidelberg, Germany) was added per well.

Proliferation was analyzed using a FACS Fortessa (BD, Bioscience, Heidelberg, Germany). Data were analyzed with the FlowJo software (Ashland, OR, USA).

### Measurement of routine serum parameters

Routine serum parameters alanine aminotransferase (ALT), aspartate aminotransferase (AST), glutamate dehydrogenase (GLDH) and alkaline phosphatase (AP) were measured in the central laboratory of clinical chemistry in RWTH Aachen University Hospital.

### Quantification and statistical analyses

For comparisons of two groups, significance was tested by unpaired two-tailed Student’s *t* test. In case of more than two groups, we employed one-way ANOVA followed by Tukey-test with adjusted *p*-value for multiple comparisons. For not normally distributed data, two groups were compared using Wilcoxon–Mann–Whitney-Test and in case of more than two groups Kruskal–Wallis test with Dunn–Bonferroni-Test was used. Data were considered significant between experimental groups as: *p < 0.05. **p < 0.01 or ***p < 0.001.

Statistical analyses of 16S microbiota data was performed using R version 3.4.3 (2017-11-30) (http://www.rproject.org) and the packages ‘phyloseq’. and ‘ggplot2’^80,90^. The permutational multivariate analysis of variance test (ADONIS) and analysis of similarities (ANOSIM) were computed with 999 permutations. For ADONIS tests, a *R*^2^ > 0.1 (effect size 10%) and *p*-value < 0.05 was considered as significant. RNA Sequencing data were analyzed using R as detailed above. The clinical cirrhosis cohort was analyzed using IBM SPSS Statistics software (Version 25). For graphic representation and statistical analysis R version 3.6, Rstudio and GraphPad Prism 8.0 were used.

### Reporting summary

Further information on research design is available in the Nature Research Reporting Summary linked to this article.

## Supplementary information

Supplementary Information

Reporting Summary

## Data Availability

Raw sequence reads of 16S rRNA amplicon sequencing are available via BioProject databases: Murine data BioProject ID:PRJNA648423 Human data BioProject ID: PRJNA842663 Raw sequence reads of bulk RNA sequencing of human samples are available via BioProject PRJNA844027 Taxonomy assignment was performed using a curated Silva database v128 (Quast C, 2013). The remaining data are available within the Article, Supplementary Information or Source Data file. Source data are provided with this paper.

## Source data

Source Data

## References

[1] Bray, F. et al. Global cancer statistics 2018: GLOBOCAN estimates of incidence and mortality worldwide for 36 cancers in 185 countries. *CA. Cancer J. Clin*. 10.3322/caac.21492 (2018). 10.3322/caac.21492 30207593

[2] Galle, P. R. et al. EASL Clinical Practice Guidelines: management of hepatocellular carcinoma. *J. Hepatol*. 10.1016/j.jhep.2018.03.019 (2018). 10.1016/j.jhep.2018.03.019 29628281

[3] Medavaram, S. & Zhang, Y. Emerging therapies in advanced hepatocellular carcinoma. *Exp. Hematol. Oncol.*10.1186/s40164-018-0109-6 (2018). 10.1186/s40164-018-0109-6 PMC6076403 30087805

[4] Forner, A., Reig, M. & Bruix, J. Hepatocellular carcinoma. *The Lancet*10.1016/S0140-6736(18)30010-2 (2018). 10.1016/S0140-6736(18)30010-2 29307467

[5] Luedde, T., Kaplowitz, N. & Schwabe, R. F. Cell death and cell death responses in liver disease: mechanisms and clinical relevance. *Gastroenterology*10.1053/j.gastro.2014.07.018 (2014). 10.1053/j.gastro.2014.07.018 PMC4531834 25046161

[6] Luedde, T. & Schwabe, R. F. NF-κB in the liver-linking injury, fibrosis and hepatocellular carcinoma. *Nat. Rev. Gastroenterol. Hepatol.*10.1038/nrgastro.2010.213 (2011). 10.1038/nrgastro.2010.213 PMC3295539 21293511

[7] Ben-Neriah, Y. & Karin, M. Inflammation meets cancer, with NF-κB as the matchmaker. *Nat. Immunol.*10.1038/ni.2060 (2011). 10.1038/ni.2060 21772280

[8] Luedde, T. et al. Deletion of NEMO/IKKγ in liver parenchymal cells causes steatohepatitis and hepatocellular carcinoma. *Cancer Cell*10.1016/j.ccr.2006.12.016 (2007). 10.1016/j.ccr.2006.12.016 17292824

[9] Kondylis, V. et al. NEMO prevents steatohepatitis and hepatocellular carcinoma by inhibiting RIPK1 kinase activity-mediated hepatocyte apoptosis. *Cancer Cell*10.1016/j.ccell.2015.10.001 (2015). 10.1016/j.ccell.2015.11.007 PMC5628166 28843278

[10] Bettermann, K. et al. TAK1 suppresses a NEMO-dependent but NF-κB-independent pathway to liver cancer. *Cancer Cell*10.1016/j.ccr.2010.03.021 (2010). 10.1016/j.ccr.2010.03.021 20478530

[11] Macpherson AJ, Heikenwalder M, Ganal-Vonarburg SC (2016). The liver at the nexus of host–microbial interactions. Cell Host Microbe.

[12] Kang, T. W. et al. Senescence surveillance of pre-malignant hepatocytes limits liver cancer development. *Nature*10.1038/nature10599 (2011). 10.1038/nature10599 22080947

[13] Delano, M. J. et al. MyD88-dependent expansion of an immature GR-1 + CD11b + population induces T cell suppression and Th2 polarization in sepsis. *J. Exp. Med*. 10.1084/jem.20062602 (2007). 10.1084/jem.20062602 PMC2118626 17548519

[14] Wang, T. et al. The adaptor protein CARD9 protects against colon cancer by restricting mycobiota-mediated expansion of myeloid-derived suppressor cells. *Immunity*10.1016/j.immuni.2018.08.018 (2018). 10.1016/j.immuni.2018.08.018 PMC6880241 30231984

[15] Levy M (2015). Microbiota-modulated metabolites shape the intestinal microenvironment by regulating NLRP6 inflammasome signaling. Cell.

[16] Elinav, E. et al. NLRP6 inflammasome regulates colonic microbial ecology and risk for colitis. *Cell*10.1016/j.cell.2011.04.022 (2011). 10.1016/j.cell.2011.04.022 PMC3140910 21565393

[17] Henao-Mejia J (2012). Inflammasome-mediated dysbiosis regulates progression of NAFLD and obesity. Nature.

[18] Schneider KM (2015). CX3CR1 is a gatekeeper for intestinal barrier integrity in mice: limiting steatohepatitis by maintaining intestinal homeostasis. Hepatology.

[19] Matson V (2018). The commensal microbiome is associated with anti PD-1 efficacy in metastatic melanoma patients. Science (80-.).

[20] Routy B (2018). Gut microbiome influences efficacy of PD-1-based immunotherapy against epithelial tumors. Science (80-.).

[21] Zheng, Y. et al. Gut microbiome affects the response to anti-PD-1 immunotherapy in patients with hepatocellular carcinoma. *J. Immunother. Cancer*10.1186/s40425-019-0650-9 (2019). 10.1186/s40425-019-0650-9 PMC6651993 31337439

[22] Plovier H (2017). A purified membrane protein from *Akkermansia muciniphila* or the pasteurized bacterium improves metabolism in obese and diabetic mice. Nat. Med..

[23] Grander, C. et al. Recovery of ethanol-induced *Akkermansia muciniphila* depletion ameliorates alcoholic liver disease. *Gut*10.1136/gutjnl-2016-313432 (2017). 10.1136/gutjnl-2016-313432 28550049

[24] Schwabe, R. F. & Greten, T. F. Gut microbiome in HCC—mechanisms, diagnosis and therapy. *J. Hepatol.*10.1016/j.jhep.2019.08.016 (2020). 10.1016/j.jhep.2019.08.016 31954488

[25] Levy M, Kolodziejczyk AA, Thaiss CA, Elinav E (2017). Dysbiosis and the immune system. Nat. Rev. Immunol..

[26] Levy M, Shapiro H, Thaiss CA, Elinav E (2017). NLRP6: a multifaceted innate immune sensor. Trends Immunol..

[27] Li M (2019). NLRP6 deficiency aggravates liver injury after allogeneic hematopoietic stem cell transplantation. Int. Immunopharmacol..

[28] Derrien, M., Vaughan, E. E., Plugge, C. M. & de Vos, W. M. *Akkermansia municiphila* gen. nov., sp. nov., a human intestinal mucin-degrading bacterium. *Int. J. Syst. Evol. Microbiol*. 10.1099/ijs.0.02873-0 (2004). 10.1099/ijs.0.02873-0 15388697

[29] Ray, A., Chakraborty, K. & Ray, P. Immunosuppressive MDSCS induced by TLR signaling during infection and role in resolution of inflammation. *Front. Cell. Infect. Microbiol.*10.3389/fcimb.2013.00052 (2013). 10.3389/fcimb.2013.00052 PMC3776133 24066282

[30] Bronte, V. et al. Recommendations for myeloid-derived suppressor cell nomenclature and characterization standards. *Nat. Commun.*10.1038/ncomms12150 (2016). 10.1038/ncomms12150 PMC4935811 27381735

[31] Kennedy, E. A., King, K. Y. & Baldridge, M. T. Mouse microbiota models: comparing germ-free mice and antibiotics treatment as tools for modifying gut bacteria. *Front. Physiol*. 10.3389/fphys.2018.01534 (2018). 10.3389/fphys.2018.01534 PMC6220354 30429801

[32] Lluch, J. et al. The characterization of novel tissue microbiota using an optimized 16S metagenomic sequencing pipeline. *PLoS ONE*10.1371/journal.pone.0142334 (2015). 10.1371/journal.pone.0142334 PMC4636327 26544955

[33] Schierwagen, R. et al. Trust is good, control is better: technical considerations in blood microbiome analysis. *Gut*10.1136/gutjnl-2019-319123 (2019). 10.1136/gutjnl-2019-319123 PMC7306979 31203205

[34] Schubert, M. et al. Perturbation-response genes reveal signaling footprints in cancer gene expression. *Nat. Commun*. 10.1038/s41467-017-02391-6 (2018). 10.1038/s41467-017-02391-6 PMC5750219 29295995

[35] Holland, C. H., Szalai, B. & Saez-Rodriguez, J. Transfer of regulatory knowledge from human to mouse for functional genomics analysis. *Biochim. Biophys. Acta—Gene Regul. Mech*. 10.1016/j.bbagrm.2019.194431 (2020). 10.1016/j.bbagrm.2019.194431 31525460

[36] Aran, D., Hu, Z. & Butte, A. J. xCell: digitally portraying the tissue cellular heterogeneity landscape. *Genome Biol*. 10.1186/s13059-017-1349-1 (2017). 10.1186/s13059-017-1349-1 PMC5688663 29141660

[37] Scott, A. C. et al. TOX is a critical regulator of tumour-specific T cell differentiation. *Nature*10.1038/s41586-019-1324-y (2019). 10.1038/s41586-019-1324-y PMC7698992 31207604

[38] Man, K. *et al*. Transcription Factor IRF4 Promotes CD8+ T cell exhaustion and limits the development of memory-like T cells during chronic infection. *Immunity*10.1016/j.immuni.2017.11.021 (2017). 10.1016/j.immuni.2017.11.021 29246443

[39] Roychoudhuri, R. et al. The transcription factor BACH2 promotes tumor immunosuppression. *J. Clin. Investig.*10.1172/JCI82884 (2016). 10.1172/JCI82884 PMC4731158 26731475

[40] Li T (2020). c-Rel is a myeloid checkpoint for cancer immunotherapy. Nat. Cancer.

[41] Zeng, D., Lin, H., Cui, J. & Liang, W. TOX3 is a favorable prognostic indicator and potential immunomodulatory factor in lung adenocarcinoma. *Oncol. Lett*. 10.3892/ol.2019.10748 (2019). 10.3892/ol.2019.10748 PMC6732997 31516613

[42] Loomba, R. et al. Gut microbiome-based metagenomic signature for non-invasive detection of advanced fibrosis in human nonalcoholic fatty liver disease. *Cell Metab*. 10.1016/j.cmet.2017.04.001 (2017). 10.1016/j.cmet.2017.04.001 PMC5502730 28467925

[43] Schnabl B, Brenner DA (2014). Interactions between the intestinal microbiome and liver diseases. Gastroenterology.

[44] Liao, L. et al. Intestinal dysbiosis augments liver disease progression via NLRP3 in a murine model of primary sclerosing cholangitis. *Gut*10.1136/gutjnl-2018-316670 (2019). 10.1136/gutjnl-2018-316670 30872395

[45] Tilg H, Cani PD, Mayer EA (2016). Gut microbiome and liver diseases. Gut.

[46] Queck A (2020). Role of portal venous platelet activation in patients with decompensated cirrhosis and TIPS. Gut.

[47] Everard A (2013). Cross-talk between *Akkermansia muciniphila* and intestinal epithelium controls diet-induced obesity. Proc. Natl Acad. Sci. USA.

[48] Ponziani, F. R. et al. Hepatocellular carcinoma is associated with gut microbiota profile and inflammation in nonalcoholic fatty liver disease. *Hepatology*10.1002/hep.30036 (2019). 10.1002/hep.30036 29665135

[49] Gabrielson A (2016). Intratumoral CD3 and CD8 T-cell densities associated with relapse-free survival in HCC. Cancer Immunol. Res..

[50] Fu J (2007). Increased regulatory T cells correlate with CD8 T-cell impairment and poor survival in hepatocellular carcinoma patients. Gastroenterology.

[51] Ma, J. et al. PD1Hi CD8+ T cells correlate with exhausted signature and poor clinical outcome in hepatocellular carcinoma. *J. Immunother. Cancer*10.1186/s40425-019-0814-7 (2019). 10.1186/s40425-019-0814-7 PMC6884778 31783783

[52] Wolf MJ (2014). Metabolic activation of intrahepatic CD8+ T cells and NKT cells causes nonalcoholic steatohepatitis and liver cancer via cross-talk with hepatocytes. Cancer Cell.

[53] Kumar V, Patel S, Tcyganov E, Gabrilovich DI (2016). The nature of myeloid-derived suppressor cells in the tumor microenvironment. Trends Immunol..

[54] Kobayashi N (2007). FOXP3+ regulatory T cells affect the development and progression of hepatocarcinogenesis. Clin. Cancer Res. J. Am. Assoc. Cancer Res..

[55] Behary J (2021). Gut microbiota impact on the peripheral immune response in non-alcoholic fatty liver disease related hepatocellular carcinoma. Nat. Commun..

[56] Dapito DH (2012). Promotion of hepatocellular carcinoma by the intestinal microbiota and TLR4. Cancer Cell.

[57] Yang, Y. et al. LPS expands MDSCs by inhibiting apoptosis through the regulation of the GATA2/let-7e axis. *Immunol. Cell Biol*. 10.1111/imcb.12204 (2019). 10.1111/imcb.12204 30221399

[58] Ost, M. et al. Myeloid-derived suppressor cells in bacterial infections. *Front. Cell. Infect. Microbiol.*10.3389/fcimb.2016.00037 (2016). 10.3389/fcimb.2016.00037 PMC4814452 27066459

[59] Farazi PA, DePinho RA (2006). Hepatocellular carcinoma pathogenesis: from genes to environment. Nat. Rev..

[60] Gabrilovich, D. I. & Nagaraj, S. Myeloid-derived suppressor cells as regulators of the immune system. *Nat. Rev. Immunol.*10.1038/nri2506 (2009). 10.1038/nri2506 PMC2828349 19197294

[61] Kalathil S, Lugade AA, Miller A, Iyer R, Thanavala Y (2013). Higher frequencies of GARP(+)CTLA-4(+)Foxp3(+) T regulatory cells and myeloid-derived suppressor cells in hepatocellular carcinoma patients are associated with impaired T-cell functionality. Cancer Res..

[62] Thaiss CA, Zmora N, Levy M, Elinav E (2016). The microbiome and innate immunity. Nature.

[63] Schneider KM, Albers S, Trautwein C (2018). Role of bile acids in the gut–liver axis. J. Hepatol..

[64] Caussy, C. et al. A gut microbiome signature for cirrhosis due to nonalcoholic fatty liver disease. *Nat. Commun*. 10.1038/s41467-019-09455-9 (2019). 10.1038/s41467-019-09455-9 PMC6440960 30926798

[65] Boursier J (2016). The severity of nonalcoholic fatty liver disease is associated with gut dysbiosis and shift in the metabolic function of the gut microbiota. Hepatology.

[66] Depommier, C. et al. Supplementation with *Akkermansia muciniphila* in overweight and obese human volunteers: a proof-of-concept exploratory study. *Nat. Med.*10.1038/s41591-019-0495-2 (2019). 10.1038/s41591-019-0495-2 PMC6699990 31263284

[67] Helmink, B. A., Khan, M. A. W., Hermann, A., Gopalakrishnan, V. & Wargo, J. A. The microbiome, cancer, and cancer therapy. *Nat. Med.*10.1038/s41591-019-0377-7 (2019). 10.1038/s41591-019-0377-7 30842679

[68] Gopalakrishnan V (2018). Gut microbiome modulates response to anti-PD-1 immunotherapy in melanoma patients. Science (80-.).

[69] Mittal, D., Gubin, M. M., Schreiber, R. D. & Smyth, M. J. New insights into cancer immunoediting and its three component phases-elimination, equilibrium and escape. *Curr. Opin. Immunol.*10.1016/j.coi.2014.01.004 (2014). 10.1016/j.coi.2014.01.004 PMC4388310 24531241

[70] Pinato DJ (2020). Immune-based therapies for hepatocellular carcinoma. Oncogene.

[71] Venegas, D. P. et al. Short chain fatty acids (SCFAs) mediated gut epithelial and immune regulation and its relevance for inflammatory bowel diseases. *Front. Immunol.*10.3389/fimmu.2019.00277 (2019). 10.3389/fimmu.2019.00277 PMC6421268 30915065

[72] Johansson MEV, Hansson GC (2012). Preservation of mucus in histological sections, immunostaining of mucins in fixed tissue, and localization of bacteria with FISH. Methods Mol. Biol..

[73] Heymann F (2012). Hepatic macrophage migration and differentiation critical for liver fibrosis is mediated by the chemokine receptor C-C motif chemokine receptor 8 in mice. Hepatology.

[74] Turnbaugh PJ (2009). A core gut microbiome in obese and lean twins. Nature.

[75] Caporaso JG (2011). Global patterns of 16S rRNA diversity at a depth of millions of sequences per sample. Proc. Natl Acad. Sci. USA.

[76] Gálvez EJC, Iljazovic A, Gronow A, Flavell R, Strowig T (2017). Shaping of intestinal microbiota in Nlrp6-and Rag2-deficient mice depends on community structure shaping of intestinal microbiota in Nlrp6-and Rag2-deficient mice depends on community structure. Cell Rep..

[77] Edgar RC (2013). UPARSE: highly accurate OTU sequences from microbial amplicon reads. Nat. Methods.

[78] Quast, C. et al. The SILVA ribosomal RNA gene database project: Improved data processing and web-based tools. *Nucleic Acids Res*. **41**, D590-6 (2013). 10.1093/nar/gks1219 PMC3531112 23193283

[79] Cole, J. R. et al. The ribosomal database project (RDP-II): introducing myRDP space and quality controlled public data. *Nucleic Acids Res*. 10.1093/nar/gkl889 (2007). 10.1093/nar/gkl889 PMC1669760 17090583

[80] McMurdie PJ, Holmes S (2013). phyloseq: an R package for reproducible interactive analysis and graphics of microbiome census data. PLoS ONE.

[81] Nearing JT (2022). Microbiome differential abundance methods produce different results across 38 datasets. Nat. Commun..

[82] Love, M. I., Huber, W. & Anders, S. Moderated estimation of fold change and dispersion for RNA-seq data with DESeq2. *Genome Biol*. **15**, 550 (2014). 10.1186/s13059-014-0550-8 PMC4302049 25516281

[83] Fernandes AD (2014). Unifying the analysis of high-throughput sequencing datasets: characterizing RNA-seq, 16S rRNA gene sequencing and selective growth experiments by compositional data analysis. Microbiome.

[84] Lelouvier, B. et al. Changes in blood microbiota profiles associated with liver fibrosis in obese patients: a pilot analysis. *Hepatology*10.1002/hep.28829 (2016). 10.1002/hep.28829 27639192

[85] Païssé, S. et al. Comprehensive description of blood microbiome from healthy donors assessed by 16S targeted metagenomic sequencing. *Transfusion*10.1111/trf.13477 (2016). 10.1111/trf.13477 26865079

[86] Anhê, F. F. et al. Type 2 diabetes influences bacterial tissue compartmentalisation in human obesity. *Nat. Metab*. 10.1038/s42255-020-0178-9 (2020). 10.1038/s42255-020-0178-9 32694777

[87] Robinson, M. D., McCarthy, D. J. & Smyth, G. K. edgeR: a bioconductor package for differential expression analysis of digital gene expression data. *Bioinformatics*10.1093/bioinformatics/btp616 (2009). 10.1093/bioinformatics/btp616 PMC2796818 19910308

[88] Ritchie, M. E. et al. Limma powers differential expression analyses for RNA-sequencing and microarray studies. *Nucleic Acids Res*. 10.1093/nar/gkv007 (2015). 10.1093/nar/gkv007 PMC4402510 25605792

[89] Dugourd, A. & Saez-Rodriguez, J. Footprint-based functional analysis of multiomic data. *Curr. Opin. Syst. Biol.*10.1016/j.coisb.2019.04.002 (2019). 10.1016/j.coisb.2019.04.002 PMC7357600 32685770

[90] Wickham, H. *ggplot2: Elegant Graphics for Data Analysis* (Springer, New York, 2009).

