# Supplementary material for: Imbalanced gut microbiota fuels hepatocellular carcinoma development by shaping the hepatic inflammatory microenvironment
Source: Nat Commun. 2022 Jul 8;13:3964. doi: 10.1038/s41467-022-31312-5 (PMC9270328; doi:10.1038/s41467-022-31312-5)
Supplement: Supplementary file 1 — Supplementary Information [file 41467_2022_31312_MOESM1_ESM.pdf]

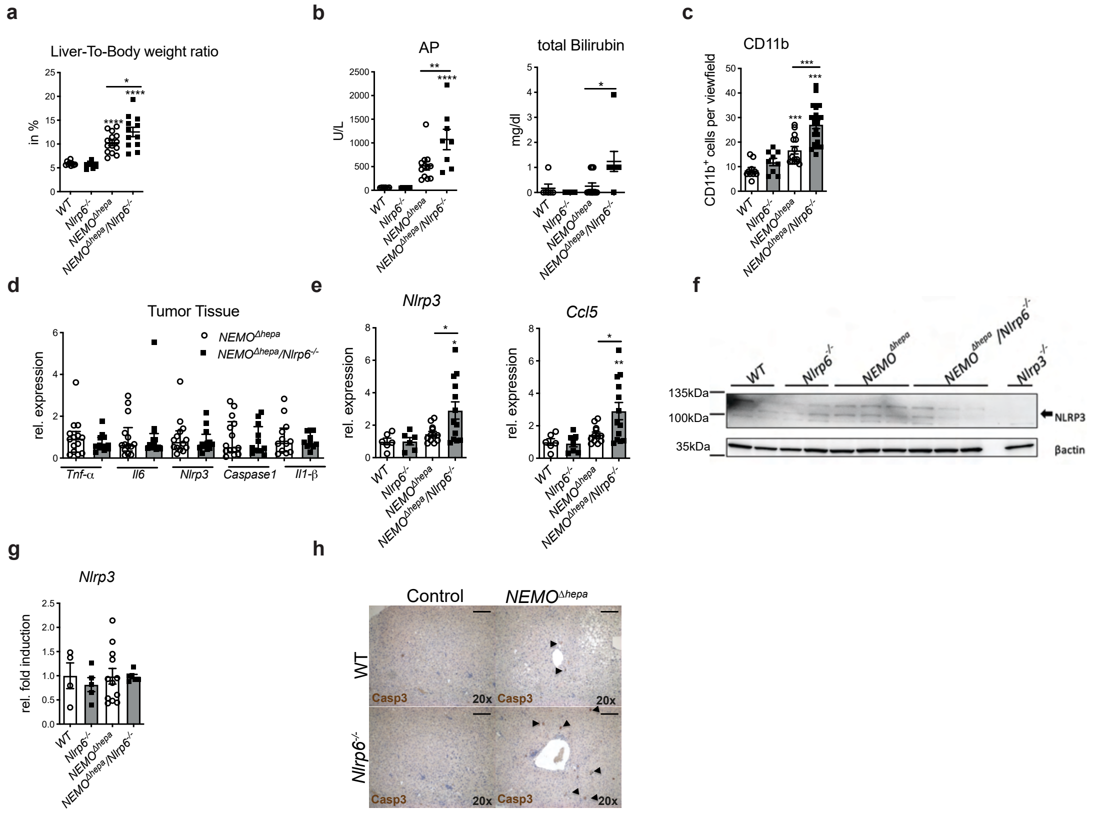

Supplementary Figure 1: Loss NLRP6 augments liver disease progression in NEMO$^{\Delta hepa}$ mice.

(a) Liver-to-Body weight ratio of 52-week-old *NEMO$^{\Delta hepa}$* (n=14), *NEMO$^{\Delta hepa}$/Nlrp6$^{-/-}$* (n=12) and respective controls (*WT* (n=9), *Nlrp6$^{-/-}$*(n=9)), one-way ANOVA with Sidak's multiple comparisons test (WT vs. *NEMO$^{\Delta hepa}$*, 95% CI -6.66 to -2.03, *p*<0.0001; *Nlrp6$^{-/-}$* vs. *NEMO$^{\Delta hepa}$/Nlrp6$^{-/-}$*,95% CI –9.32 to -4.54, *p*=<0.0001; *NEMO$^{\Delta hepa}$* vs. *NEMO$^{\Delta hepa}$/Nlrp6$^{-/-}$*, 95% CI -4.41 to -0.15, *p*=0.033) **(b)** Serum AP and total bilirubin levels of 52-week-old *NEMO$^{\Delta hepa}$*(n=12), *NEMO$^{\Delta hepa}$/Nlrp6$^{-/-}$* (n=8) and respective controls (*WT* (n=6), *Nlrp6$^{-/-}$*(n=6)); one-way ANOVA with Sidak's multiple comparisons test (AP: WT vs. *NEMO$^{\Delta hepa}$*, 95% CI -923.6 to -0.28, *p=ns*; *Nlrp6$^{-/-}$* vs. *NEMO$^{\Delta hepa}$/Nlrp6$^{-/-}$*,95% CI –1515 to -516.9, *p*=<0.0001; *NEMO$^{\Delta hepa}$* vs. *NEMO$^{\Delta hepa}$/Nlrp6$^{-/-}$*, 95% CI -975.9 to -132.5, *p*=0.0072; Bilirubin: WT vs. *NEMO$^{\Delta hepa}$*, 95% CI -0.9147 to 0.7496, *p=ns*; *Nlrp6$^{-/-}$* vs. *NEMO$^{\Delta hepa}$/Nlrp6$^{-/-}$*,95% CI -1.746 to -0.2269, *p*=0.014) **(c)** Histological quantification CD11b positive cells per viewfield on IF stainings of *NEMO$^{\Delta hepa}$(n=14), NEMO$^{\Delta hepa}$/Nlrp6$^{-/-}$(n=23)* and respective control (*WT* (n=10), *Nlrp6$^{-/-}$* (n=9)) mice. Data points represent view fields, one-way ANOVA with Sidaks's multiple comparisons test (WT vs. *NEMO$^{\Delta hepa}$*, 95% CI -14.54 to -1.684, *p*=0.009; *Nlrp6$^{-/-}$* vs. *NEMO$^{\Delta hepa}$/Nlrp6$^{-/-}$*,95% CI -24.41 to -12.65, *p*=<0.0001; *NEMO$^{\Delta hepa}$* vs. *NEMO$^{\Delta hepa}$/Nlrp6$^{-/-}$*, 95% CI -15.68 to -5.152, *p<0.0001*. **(d)** Analysis of mRNA expression of depicted genes in tumor tissue and macroscopically tumor free surrounding tissue (*NEMO$^{\Delta hepa}$(n=12-14), NEMO$^{\Delta hepa}$/Nlrp6$^{-/-}$(n=10-12)*), *TNFα, IL 6, Nlrp3, Caspase1, IL-1β, unpaired t-test: TNFα: NEMO$^{\Delta hepa}$* vs. *NEMO$^{\Delta hepa}$/Nlrp6$^{-/-}$*, 95% CI -0.6539 to 1.121, *p=n.s; IL 6: NEMO$^{\Delta hepa}$* vs. *NEMO$^{\Delta hepa}$/Nlrp6$^{-/-}$*, 95% CI -1,258 to 1,249, *p=n.s; Nlrp3: NEMO$^{\Delta hepa}$* vs. *NEMO$^{\Delta hepa}$/Nlrp6$^{-/-}$*, 95% CI -0.7768 to 0.9108, *p=n.s; Caspase1: NEMO$^{\Delta hepa}$* vs. *NEMO$^{\Delta hepa}$/Nlrp6$^{-/-}$*, 95% CI -0.5726 to 1.312, *p=n.s; IL-1β: NEMO$^{\Delta hepa}$* vs. *NEMO$^{\Delta hepa}$/Nlrp6$^{-/-}$*, 95% CI -0.3358 to 0.9637, *p=n.s*. **(e)** Relative Gene expression in 52-week-old *NEMO$^{\Delta hepa}$* (n=12), *NEMO$^{\Delta hepa}$/Nlrp6$^{-/-}$* (n=13) and respective controls (*WT* (n=7), *Nlrp6$^{-/-}$*(n=8)) *of Nlrp3 and Ccl5, Nlrp3: WT* vs.

NEMO$^{\Delta hepa}$*95%* -2.005 to 1.075, p=n.s; *NEMO*$^{\Delta hepa}$ vs. *NEMO*$^{\Delta hepa}$*/Nlrp6*$^{-/-}$*: 95% CI* -2.677 to -0.2105, *p*=0.0176, *Ccl5: WT* vs. *NEMO*$^{\Delta hepa}$*95%* -1.986 to 1.055, p=n.s; *NEMO*$^{\Delta hepa}$ vs. *NEMO*$^{\Delta hepa}$*/Nlrp6*$^{-/-}$*: 95% CI* -2.661 to -0.2261, p=.016*.* **(f)** Immunoblot analysis of liver protein extracts from 52-week-old mice of all indicated genotypes for NLRP3 and βactin as loading control. Liver extract of an *Nlrp3*$^{-/-}$ mouse serves as negative control (last lane), representative of 2 experiments **(g)** Relative gene expression in 13-week-old *NEMO*$^{\Delta hepa}$ (n=12)*, NEMO*$^{\Delta hepa}$*/Nlrp6*$^{-/-}$ (n=6) and respective controls (*WT* (n=4)*, Nlrp6*$^{-/-}$(n=5)*) of Nlrp3, one-way ANOVA* **(h)** Representative IHC staining of Cleaved Caspase 3 of 52-week-old *WT, Nlrp6*$^{-/-}$*, NEMO*$^{\Delta hepa}$*, and NEMO*$^{\Delta hepa}$*/Nlrp6*$^{-/-}$ livers, representative of 2 experiments; Scale bar: 100 µm. All Data are presented as the mean ± standard error of the mean (SEM) and considered significant at p < 0.01 (**), p < 0.001 (***) and p < 0.0001 (****). Individual data points represent biological replicates unless otherwise stated. Source data are provided as a Source data file.

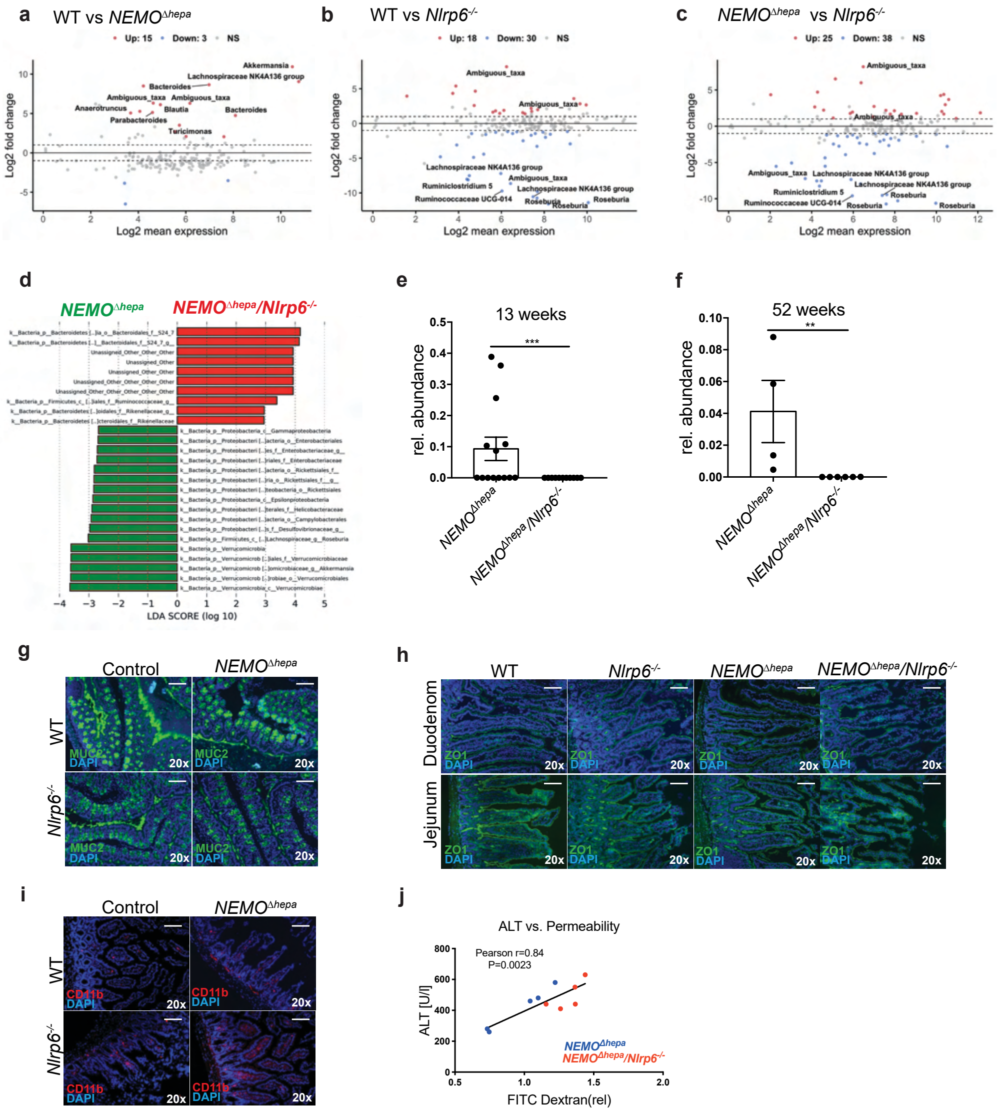

Supplementary Figure 2: Loss of NLRP6 results in intestinal dysbiosis and barrier impairment correlating with steatohepatitis activity and tumor burden.
(a) Analysis for differential abundance of microbiota via DESeq analysis of WT (n=8) and *NEMO*$^{\Delta hepa}$ (n=13) mice. **(b)** Analysis for differential abundance of microbiota via

DESeq analysis of WT (n=8) and *Nlrp6*$^{-/-}$ mice (n=9). **(c)** Analysis for differential abundance of microbiota via DESeq analysis of *NEMO*$^{\Delta hepa}$ (n=13) and *Nlrp6*$^{-/-}$ mice (n=9). **(d)** LDA effect size analysis identifies differentially abundant taxa between 52w old mouse groups (*NEMO*$^{\Delta hepa}$/*Nlrp6*$^{-/-}$ (n=6), *NEMO*$^{\Delta hepa}$ (n=4)).**(e)** 13-week-old *NEMO*$^{\Delta hepa}$/*Nlrp6*$^{-/-}$ (n=11) mice lack *A. muciniphila compared to NEMO*$^{\Delta hepa}$ (n=14), Mann Whitney test: *NEMO*$^{\Delta hepa}$ vs. *NEMO*$^{\Delta hepa}$/*Nlrp6*$^{-/-}$, $p<0.0001$ **(f)** 52-week-old *NEMO*$^{\Delta hepa}$/*Nlrp6*$^{-/-}$ (n=6) mice lack *A. muciniphila* compared to *NEMO*$^{\Delta hepa}$ (n=4), Mann Whitney test: *NEMO*$^{\Delta hepa}$ vs. *NEMO*$^{\Delta hepa}$/*Nlrp6*$^{-/-}$, $p<0.001$. **(g)** Representative pictures of immunofluorescence staining of MUC2 in colon of *NEMO*$^{\Delta hepa}$, *NEMO*$^{\Delta hepa}$/*Nlrp6*$^{-/-}$ and respective controls (*WT, Nlrp6*$^{-/-}$). Nuclei were counterstained with DAPI, representative of 2 experiments; Scale bar: 100 µm. **(h)** Representative pictures of immunofluorescence staining of ZO-1 in duodenum and jejunum of *NEMO*$^{\Delta hepa}$, *NEMO*$^{\Delta hepa}$/*Nlrp6*$^{-/-}$ and respective controls (*WT, Nlrp6*$^{-/-}$). Nuclei were counterstained with DAPI, representative of 2 experiments; Scale bar: 100 µm. **(i)** Representative pictures of immunofluorescence staining of CD11b in ileum of *NEMO*$^{\Delta hepa}$, *NEMO*$^{\Delta hepa}$/*Nlrp6*$^{-/-}$ and respective controls (*WT, Nlrp6*$^{-/-}$). Nuclei were counterstained with DAPI, representative of 2 experiments; Scale bar: 100 µm. **j)** Strong correlation of intestinal barrier function evidenced by FITC-dextran permeability with serum ALT levels in *NEMO*$^{\Delta hepa}$ and *NEMO*$^{\Delta hepa}$/*Nlrp6*$^{-/-}$ mice (Pearson), 10 pairs, two-tailed, *p*=0.0023). All Data are presented as the mean ± standard error of the mean (SEM) and considered significant at p < 0.01 (**), p < 0.001 (***) and p < 0.0001 (****). Individual data points represent biological replicates unless otherwise stated. Source data are provided as a Source data file.

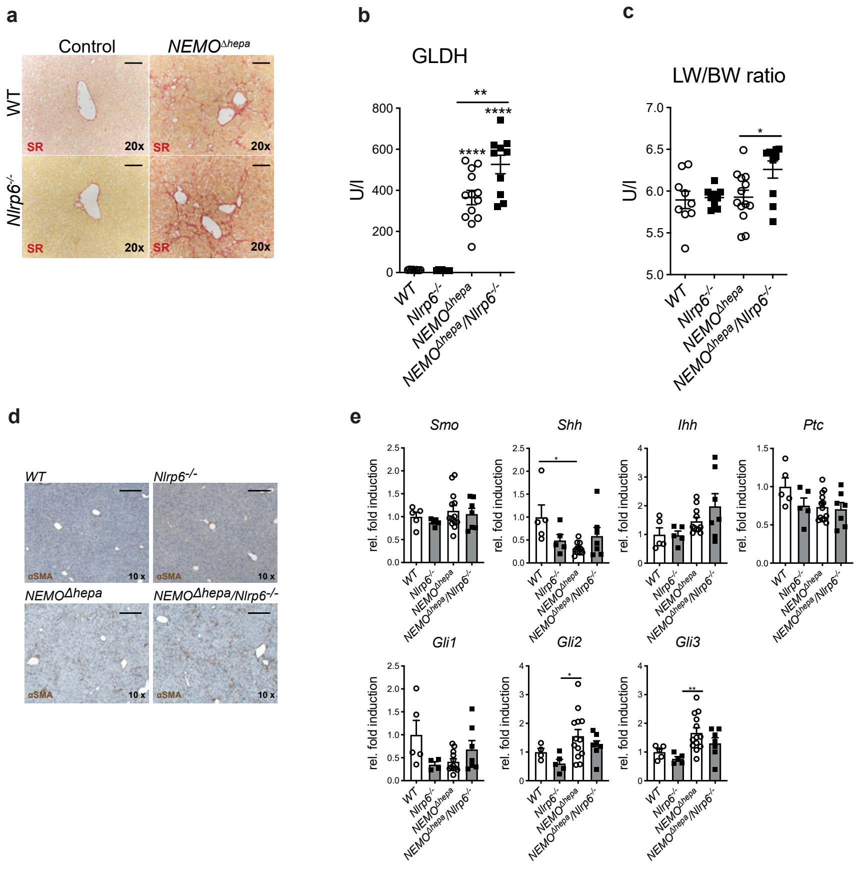

Supplementary Figure 3: Increased steatohepatitis activity in 13-week-old *NEMO*$^{\Delta hepa}$/*Nlrp6*$^{-/-}$ mice.

(a) Representative pictures of Sirius red stained liver sections of 13-week-old *WT,* $Nlrp6^{-/-}$, $NEMO^{\Delta hepa}$, $NEMO^{\Delta hepa}/Nlrp6^{-/-}$, representative of 2 experiments; Scale bar: 100 µm. **(b)** Serum GLDH levels of 13-week-old *WT* (n=8)*,* $Nlrp6^{-/-}$(n=8), $NEMO^{\Delta hepa}$ (n=13) and $NEMO^{\Delta hepa}/Nlrp6^{-/-}$ (n=10) mice, one-way ANOVA, Bonferroni's multiple comparison test *WT* vs. $NEMO^{\Delta hepa}$ *95%* -482.1 to -222.9, p<.0001, $Nlrp6^{-/-}$ vs. $NEMO^{\Delta hepa}/Nlrp6^{-/-}$ *95%* -651.6 to -378.1, p<0.0001, $NEMO^{\Delta hepa}$ vs. $NEMO^{\Delta hepa}/Nlrp6^{-/-}$*95%CI* -282.3 to -39.73 p=.0043. **(c)** Liver-to-Body weight ratio of 13-week-old $NEMO^{\Delta hepa}$ (n=13)*,* $NEMO^{\Delta hepa}/Nlrp6^{-/-}$(n=10) and respective controls (*WT* n=9)*,* $Nlrp6^{-/-}$ (n=9)*,* one-way ANOVA, Bonferroni's multiple comparison test, *WT* vs. $NEMO^{\Delta hepa}/Nlrp6^{-/-}$ *95%* -0.3590 to 0.2915, p=n.s, $Nlrp6^{-/-}$ vs. $NEMO^{\Delta hepa}/Nlrp6^{-/-}$ *95%* -0.3308 to 0.3197, p=n.s, $NEMO^{\Delta hepa}$ vs. $NEMO^{\Delta hepa}/Nlrp6^{-/-}$*95%CI* -0.6046 to -0.05655, p=.015*, pooled data from >3 independent experiments* **(d)** Representative pictures and quantification of immunohistochemical (IHC) staining of αSMA stained liver sections of 13-week-old *WT*(n=5)*,* $Nlrp6^{-/-}$(n=3)*,* $NEMO^{\Delta hepa}$ (n=13)*, and* $NEMO^{\Delta hepa}/Nlrp6^{-/-}$ (n=8) mice; Scale bar: 200 µm. **(e)** RT-qPCR analysis of hedgehog associated mRNA expression (*Smo, Shh, Ihh, Ptc, Gli1, Gli2 and Gli3*) in whole liver of $NEMO^{\Delta hepa}$ (n=13)*,* $NEMO^{\Delta hepa}/Nlrp6^{-/-}$(n=7) and respective controls (*WT* (n=5)*,* $Nlrp6^{-/-}$ (n=5), one-way ANOVA, Tukey's multiple comparison test Shh: *WT* vs. $NEMO^{\Delta hepa}$ *95%CI:* 0.1046 to 1.255, p=0.0165; Gli2: $Nlrp6^{-/-}$ vs. $NEMO^{\Delta hepa}$ *95%CI:* -1.859 to -0.04125, p=0.038, Gli3: $Nlrp6^{-/-}$ vs. $NEMO^{\Delta hepa}$ *95%CI:* -1.626 to -0.1840, p=0.0099). All Data are presented as the mean ± standard error of the mean (SEM) and considered significant at p < 0.01 (**), p < 0.001 (***) and p < 0.0001 (****). Individual data points represent biological replicates unless otherwise stated. Source data are provided as a Source data file.

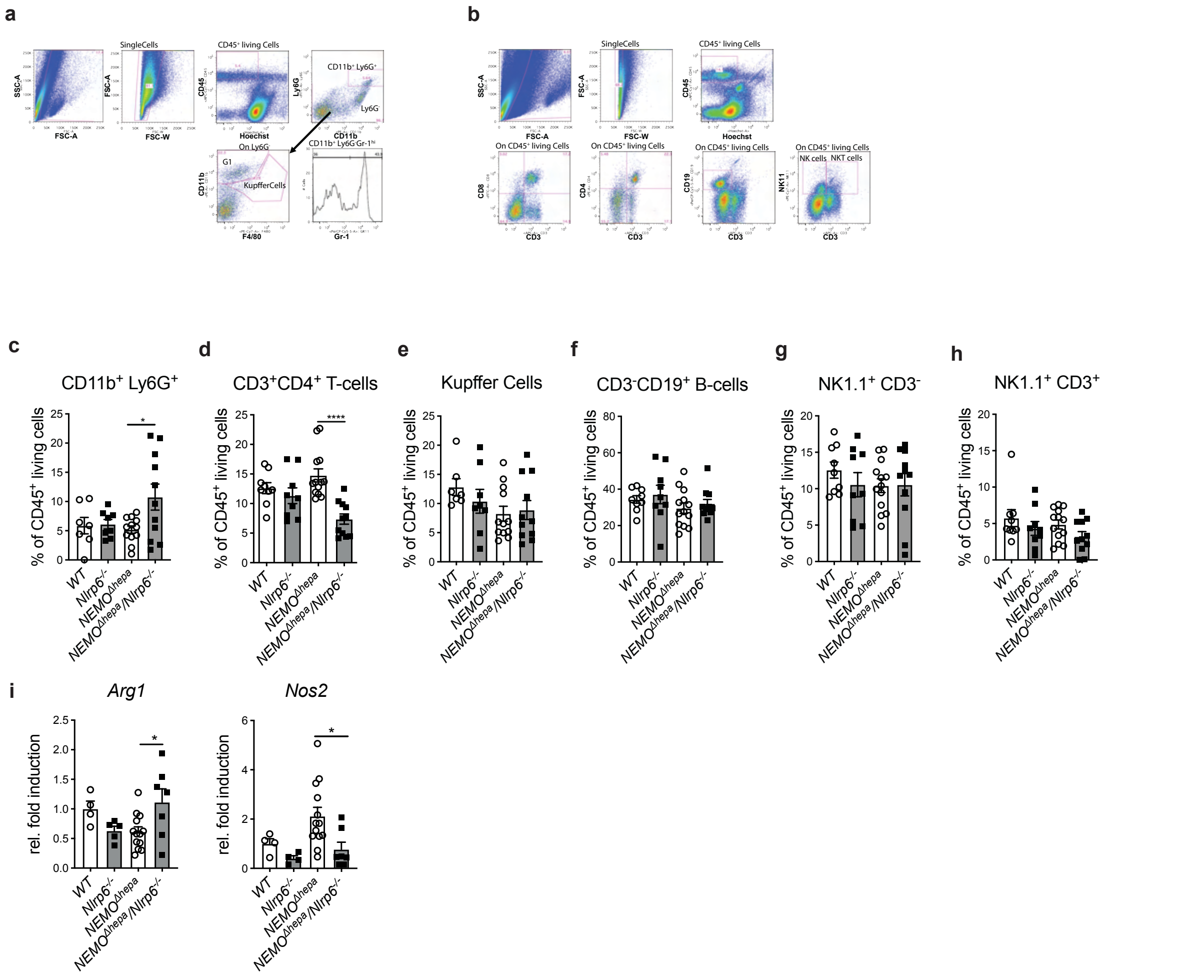

Supplementary Figure 4: *In vivo* immune cell composition.

Flow cytometry gating strategy for **(a)** CD11b^{+}Ly6G^{+}, Kupffer cells and **(b)** CD3^{-}CD19^{+} B cells, NK1.1^{+}CD3^{-} and NK1.1^{+}CD3^{+}. Flow cytometry analysis of **(c)** CD11b^{+}Ly6G^{+} **(d)** CD3^{+}CD4^{+} T cells **(e)** Kupffer cells **(f)** CD3^{-}CD19^{+} B cells **(g)** NK1.1^{+}CD3^{-} **(h)** NK1.1^{+}CD3^{+} isolated from whole liver of 13-week-old $NEMO^{\Delta hepa}$ (n=12), $NEMO^{\Delta hepa}/Nlrp6^{-/-}$(n=11) and respective controls (*WT* (n=7), $Nlrp6^{-/-}$ (n=8), one-way ANOVA, Sidak's multiple comparison test, CD11: $NEMO^{\Delta hepa}$ vs. $NEMO^{\Delta hepa}/Nlrp6^{-/-}$ *95% CI* -10.61 to -0.5226, p=.0256; CD3^{+}CD4^{+} T cells: $NEMO^{\Delta hepa}$ vs. $NEMO^{\Delta hepa}/Nlrp6^{-/-}$ *95% CI* 3.656 to 11.13, p<0.0001*)*. **(i)** RT-qPCR analysis of *Arg1 and Nos2* in whole liver of $NEMO^{\Delta hepa}$ (n=13)*,* $NEMO^{\Delta hepa}/Nlrp6^{-/-}$(n=7) and respective controls (*WT* (n=4)*,* $Nlrp6^{-/-}$(n=4*)*, one-way ANOVA, Tukey's multiple comparison test, Arg1: $NEMO^{\Delta hepa}$ vs. $NEMO^{\Delta hepa}/Nlrp6^{-/-}$ *95% CI* -0.9804 to -0.01621, p=.0409, NOS2: $NEMO^{\Delta hepa}$ vs. $NEMO^{\Delta hepa}/Nlrp6^{-/-}$ *95% CI* 0.03632 to 2.650, p=.0424. All Data are presented as the mean ± standard error of the mean (SEM) and considered significant at p < 0.01 (**), p < 0.001 (***) and p < 0.0001 (****). Individual data points represent biological replicates unless otherwise stated. Source data are provided as a Source data file.

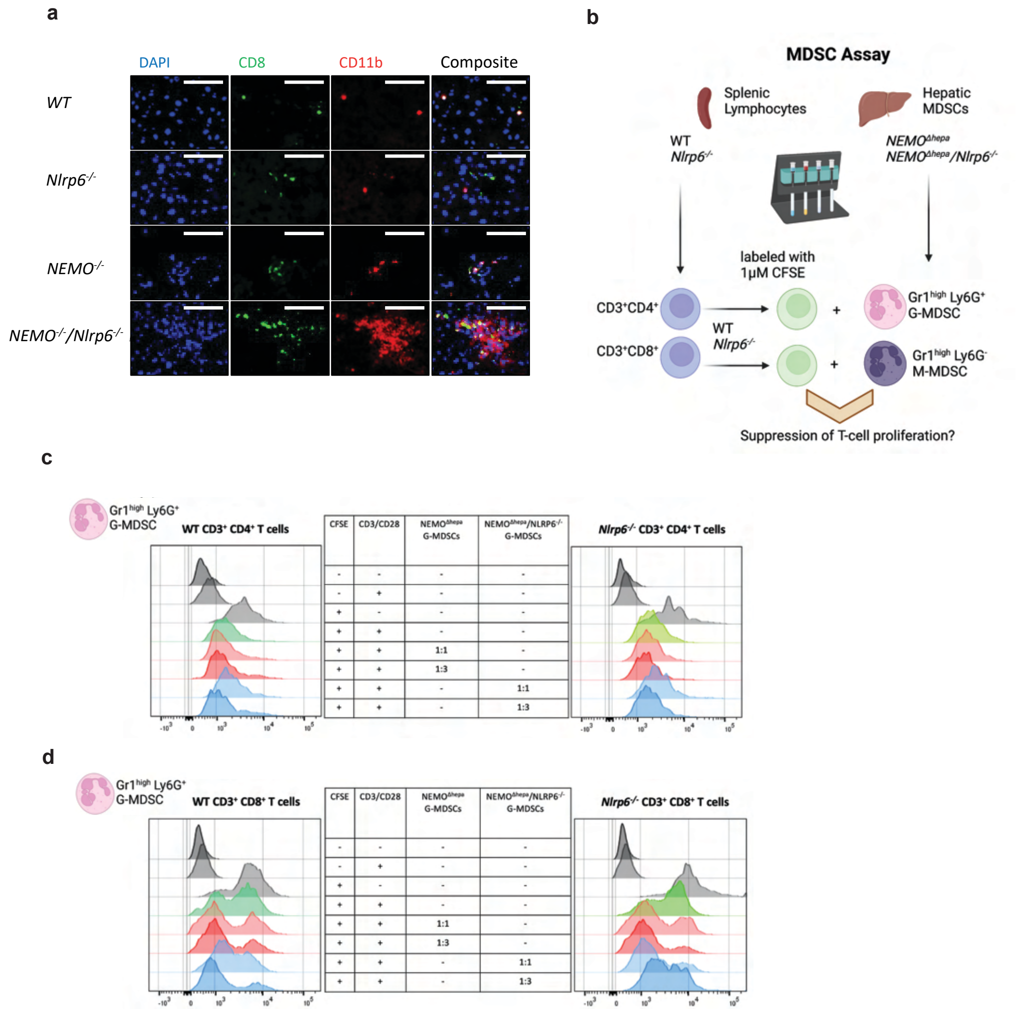

Supplementary Figure 5: *In vitro* T cell proliferation assay.

(a) Representative pictures of immunofluorescence (IF) double stainings of CD11b and CD8 stained liver sections of 13-week-old *WT,* $Nlrp6^{-/-}$*,* $NEMO^{\Delta hepa}$*, and* $NEMO^{\Delta hepa}/Nlrp6^{-/-}$ livers, representative of 2 experiments; Scale bar: 50 µm. **(b)** Schematic overview (created with BioRender) of the MDSC assay. M-MDSC, G-MDSC

were isolated from $NEMO^{\Delta hepa}/Nlrp6^{-/-}$ (n=3) and $NEMO^{\Delta hepa}$ (n=3) livers. T cells were isolated from WT (n=2) and $Nlrp6^{-/-}$ (n=2) spleen using MACS. The suppressive capacity of gMDSCs and mMDSCs isolated from $NEMO^{\Delta hepa}/Nlrp6^{-/-}$ and $NEMO^{\Delta hepa}$ livers on splenic T cells was measured by flow cytometry. **(c)** T cell proliferation upon co-culture of WT or $Nlrp6^{-/-}$ CD3$^+$ CD4$^+$ T-cells or **(d)** CD3$^+$ CD8$^+$ T-cells and gMDSCs isolated from $NEMO^{\Delta hepa}/Nlrp6^{-/-}$ or $NEMO^{\Delta hepa}$ livers; representative of 2 experiments. Abbreviations: G-MDSC, granulocytic myeloid-derived suppressor cell; M-MDSC, monocytic myeloid-derived suppressor cell.

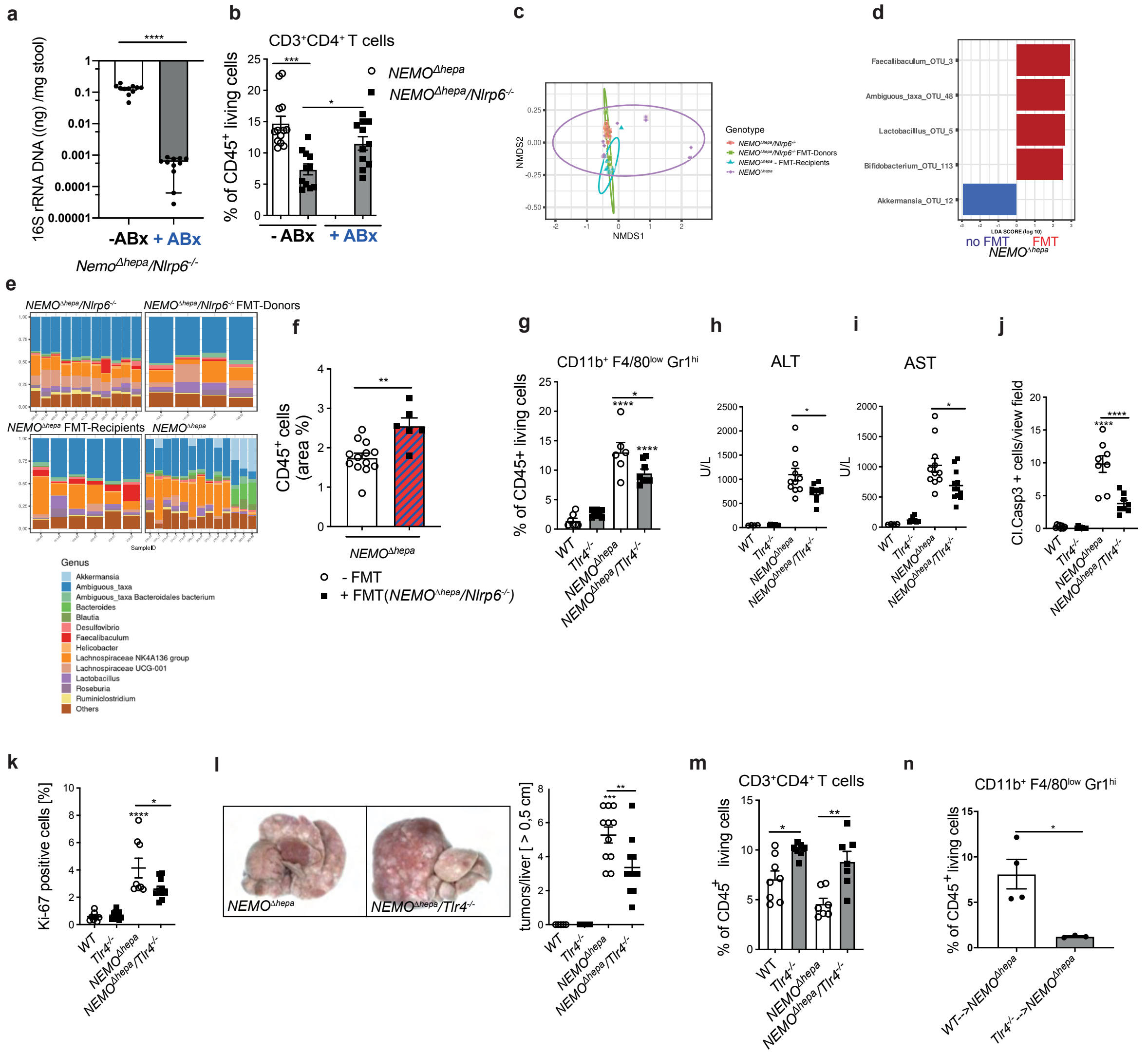

Supplementary Figure 6: Loss of TLR4 protects $NEMO^{\Delta hepa}$ mice from liver disease progression.

(a) 16s rRNA ng/mg stool determined by real time quantitative PCR in 13-week-old $NEMO^{\Delta hepa}/Nlrp6^{-/-}$ with (n=11) or without (n=11) 5 wks Abx treatment (Mann-Whitney-U-Test). **(b)** Flow cytometry analysis of CD3$^+$CD4$^+$ T-cells isolated from whole liver of 13-week-old $NEMO^{\Delta hepa}$ (n=13) and $NEMO^{\Delta hepa}/Nlrp6^{-/-}$ with (n=11) or without (n=11) 5 wks Abx treatment. $NEMO^{\Delta hepa}$ and $NEMO^{\Delta hepa}/Nlrp6^{-/-}$ groups shared with Fig. 3 **(c)** NMDS ordination based on bray-curtis dissimilarity index showing microbiota of $NEMO^{\Delta hepa}$ (n=13), $NEMO^{\Delta hepa}/Nlrp6^{-/-}$ (n=11) and $NEMO^{\Delta hepa}$ – FMT recipient mice (n=6) treated with from $NEMO^{\Delta hepa}/Nlrp6^{-/-}$ FMT Donors (n=4). $NEMO^{\Delta hepa}$ and $NEMO^{\Delta hepa}/Nlrp6^{-/-}$ groups shared with Fig. 2 **(d)** Linear discriminant analysis (LDA) of effect size (LEfSe) between $NEMO^{\Delta hepa}$ mice treated with $NEMO^{\Delta hepa}/Nlrp6^{-/-}$ (+FMT)

(n=6) microbiota or without treatment (-FMT) (n=13). **(e)** Microbiota composition (genus level) of *NEMO*$^{\Delta hepa}$ mice treated with *NEMO*$^{\Delta hepa}$*/Nlrp6*$^{-/-}$ (+FMT) (n=6) microbiota or without treatment (-FMT) (n=13) as well as *NEMO*$^{\Delta hepa}$*/Nlrp6*$^{-/-}$ mice and *NEMO*$^{\Delta hepa}$*/Nlrp6*$^{-/-}$ FMT Donors (n=4). **(f)** Quantification of immunohistochemical (IHC) staining of CD45 stained liver sections *NEMO*$^{\Delta hepa}$ mice treated with *NEMO*$^{\Delta hepa}$*/Nlrp6*$^{-/-}$ (+FMT, n=6) microbiota or without treatment (-FMT, n=13), unpaired t-test 95%CI 0.3571 to 1.258, p=0.0015 **(g)** Flow cytometry analysis of mMDSC from whole liver of 8-week-old *NEMO*$^{\Delta hepa}$ (n=6) and *NEMO*$^{\Delta hepa}$*/Tlr4*$^{-/-}$ (n=8) mice and their respective controls (*WT* (n=7)*, Tlr4*$^{-/-}$(n=8), *one-way ANOVA,* Tukey's multiple comparisons test, *WT* vs. *NEMO*$^{\Delta hepa}$ *95%CI* -14.90 to -8.378, p<0.0001; *Tlr4*$^{-/-}$ vs. *NEMO*$^{\Delta hepa}$*/Tlr4*$^{-/-}$ 95%CI -9.739 to -3.880, p<0.0001; *NEMO*$^{\Delta hepa}$ vs. *NEMO*$^{\Delta hepa}$*/Tlr4*$^{-/-}$ 95%CI 0.4041 to 6.733, p=0.0229). **(h)** Serum alanine aminotransferase (ALT) and **(i)** aspartate aminotransferase (AST) levels of 8-week-old *NEMO*$^{\Delta hepa}$ (n=11) and *NEMO*$^{\Delta hepa}$*/Tlr4*$^{-/-}$ (n=11) mice and their respective controls (*WT* (n=4)*, Tlr4*$^{-/-}$(n=9), *one-way ANOVA,* Tukey's multiple comparisons test, *ALT: NEMO*$^{\Delta hepa}$ vs. *NEMO*$^{\Delta hepa}$*/Tlr4*$^{-/-}$ 95%CI 68.56 to 668.7, p=0.0113; *AST: NEMO*$^{\Delta hepa}$ vs. *NEMO*$^{\Delta hepa}$*/Tlr4*$^{-/-}$ 95%CI 38.10 to 632.7, p=0.0222), pooled data from >2 independent experiments*.* **(j)** Histological quantification of the number of Cleaved Caspase 3 positive cells per viewfield on immunohistochemical stainings of *NEMO*$^{\Delta hepa}$(n=8)*, NEMO*$^{\Delta hepa}$*/TLR4*$^{-/-}$(n=8) and respective controls (*WT* (n=8)*, Tlr4*$^{-/-}$(n=9), *one-way ANOVA,* Tukey's multiple comparisons test*: WT* vs. *NEMO*$^{\Delta hepa}$ *95%CI* -12.14 to -6.950, p<.0001; *NEMO*$^{\Delta hepa}$ vs. *NEMO*$^{\Delta hepa}$*/Tlr4*$^{-/-}$ 95%CI 3.427 to 8.620, p<.0001*)*. **(k)** Quantification of the percentage of KI67 positive cells of *NEMO*$^{\Delta hepa}$ (n=8)*, NEMO*$^{\Delta hepa}$*/TLR4*$^{-/-}$ (n=11) and respective controls (*WT* (n=8)*, Tlr4*$^{-/-}$ (n=11), *one-way ANOVA,* Tukey's multiple comparisons test*: WT* vs. *NEMO*$^{\Delta hepa}$ *95%CI* -4.958 to -2.194, p<0.0001; *NEMO*$^{\Delta hepa}$ vs. *NEMO*$^{\Delta hepa}$*/Tlr4*$^{-/-}$ 95%CI 0.2636 to 2.832, p=0.0131*)*. **(l)** Macroscopic analysis of tumors in livers of 52-week-old *WT* (n=5)*, Tlr4*$^{-/-}$ (n=5)*, NEMO*$^{\Delta hepa}$ (n=11) and *NEMO*$^{\Delta hepa}$*/TLR4*$^{-/-}$ (n=11) mice, *one-way ANOVA,* Dunett's multiple comparisons test*: WT* vs. *NEMO*$^{\Delta hepa}$ *95%CI* 3.45 to 7.09, p<0.0001; *NEMO*$^{\Delta hepa}$ vs. *NEMO*$^{\Delta hepa}$*/Tlr4*$^{-/-}$ 95%CI 0.47 to 3.348, p=.0071*)* **(m)** Flow cytometry analysis of $CD3^+CD4^+$ T-cells isolated from whole liver of *WT* (n=8)*,Tlr4*$^{-/-}$ (n=7)*, NEMO*$^{\Delta hepa}$ (n=7)*,* and *NEMO*$^{\Delta hepa}$*/Tlr4*$^{-/-}$(n=7) mice, *one-way ANOVA,* Tukey's multiple comparisons test, WT vs. *Tlr4*$^{-/-}$ *95%CI* -5.585 to -0.1915, p=0.0326; *NEMO*$^{\Delta hepa}$ vs. *NEMO*$^{\Delta hepa}$*/Tlr4*$^{-/-}$ 95%CI -7.005 to -1.435, p=0.0017. **(n)** Flow cytometry analysis of mMDSCs isolated from whole liver of bone-marrow chimeras (*WT*→ *NEMO*$^{\Delta hepa}$ (n=4)*; Tlr4*$^{-/-}$ → *NEMO*$^{\Delta hepa}$(n=3), unpaired two-tailed Student's t-test, 95% CI -11.81 to -1.929, p=0.016*).* All Data are presented as the mean ± standard error of the mean (SEM) and considered significant at p < 0.05 (*), p < 0.01 (**), p < 0.001 (***) and p < 0.0001 (****), respectively. Individual data points represent biological replicates unless otherwise stated. Source data are provided as a Source data file.

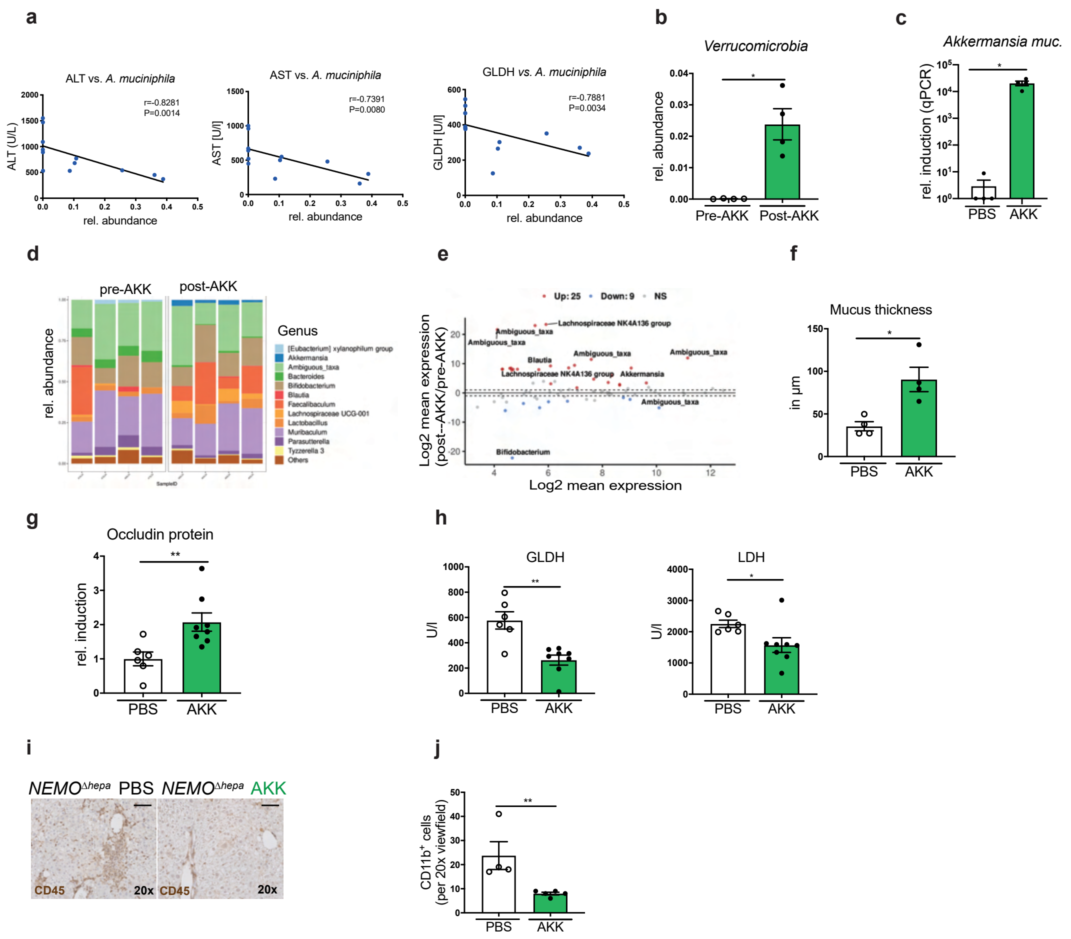

Supplementary Figure 7: *Akkermansia muciniphila* supplementation ameliorates liver disease in *NEMO*$^{\Delta hepa}$ mice.

(a) Inverse correlation (Spearman, two-tailed, 13 pairs) between ALT, AST as well as GLDH and abundance of *A. muciniphila* in 13 weeks old *NEMO*$^{\Delta hepa}$ mice (n=13)**. (b)** Relative abundance of *Verrucomicrobia* in stool of *NEMO*$^{\Delta hepa}$ mice before (n=4) and after (n=4) 5-week treatment with AKK, Mann-Whitney test, *P*=0.0286. **(c)** Relative induction of A. muciniphila abundance after supplementation of *NEMO*$^{\Delta hepa}$ (n=4) compared to PBS treated controls (n=4) determined by real-time quantitative PCR; Mann-Whitney test, *P*=0.0286 **(d)** Microbiota composition (family level) of *NEMO*$^{\Delta hepa}$ mice (n=4) before and after 5-week treatment with *A. muciniphila*. **(e)** Analysis for differential abundance of microbiota via DESeq analysis of *NEMO*$^{\Delta hepa}$ mice (n=4) before and after transfer of AKK. **(f)** Quantification of colonic mucus layers based on MUC2 immunofluorescence stainings of *A. muciniphila* (n=4) or PBS (n=4) treated mice. **(g)** Quantification of occludin protein normalized to β-actin of colon tissue extracts in Immunoblot analysis in PBS (n=6) and AKK (n=8) treated *NEMO*$^{\Delta hepa}$, unpaired two-tailed Student's t-test, *P*= 0.0108, 95% CI 0.2977 to 1.853. **(h)** Serum GLDH and LDH levels in PBS (n=6) and *A. muciniphila* (n=8) treated *NEMO*$^{\Delta hepa}$ mice, unpaired two-tailed Student's t-test, GLDH: p=0.0012, LDH: p=0.0377. **(i)** Representative pictures of immunohistochemical staining against CD45 in *NEMO*$^{\Delta hepa}$ mice treated with PBS or *A. muciniphila* for 5 weeks, representative of 2 experiments; Scale bar: 100 µm. **(j)** Histological quantification of the number of CD11b positive cells per viewfield on IF stainings of *NEMO*$^{\Delta hepa}$ mice treated with PBS (n=4) or *A. muciniphila* (n=5), unpaired two-tailed Student's t-test, p=0.018. All Data are presented as the mean ± standard error of the mean (SEM). Individual data points represent

biological replicates unless otherwise stated. Source data are provided as a Source data file.

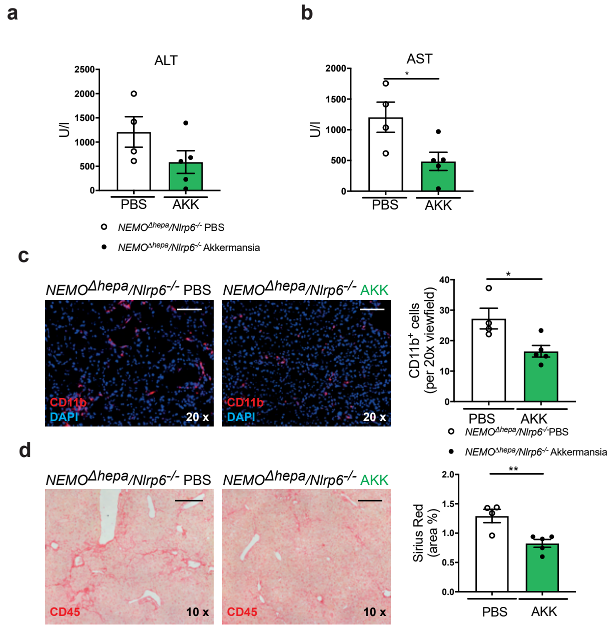

Supplementary Figure 8: *Akkermansia muciniphila* supplementation ameliorates liver disease in *NEMO*$^{\Delta hepa}$*/Nlrp6*$^{-/-}$ mice.

(A + B) Serum aspartate aminotransferase (AST) and alanine aminotransferase (ALT) levels of *NEMO*$^{\Delta hepa}$*/Nlrp6*$^{-/-}$ mice either treated with PBS (n=4) or AKK (n=5) for 5 weeks; ALT: unpaired two-tailed Student's t-test, *P*=0.1484, AST: unpaired two-tailed Student's t-test, *P*=0.0340, 95% CI -1366 to -72.35. **(C)** Representative pictures and quantification of immunofluorescence staining for CD11b in either PBS (n=4) or AKK (n=5) treated *NEMO*$^{\Delta hepa}$*/Nlrp6*$^{-/-}$ mice. Nuclei were counterstained with DAPI. 2 independent experiments; Scale bar: 100 µm. Unpaired two-tailed Student's t-test, *P*=0.0225, 95% CI -19.44 to -2.024. **(D)** Representative pictures and quantification of SR-stained liver sections in either PBS (n=4) or AKK (n=5) treated *NEMO*$^{\Delta hepa}$*/Nlrp6*$^{-/-}$ mice. 2 independent experiments; Scale bar: 200 µm. Unpaired two-tailed Student's t-test, *P*=0.0072, 95% CI -0.7613 to -0.1717. Data are presented as the mean ± standard error of the mean (SEM) and considered significant at $p < 0.05$ (*), $p < 0.01$ (**), respectively. Individual data points represent biological replicates unless otherwise stated. Source data are provided as a Source data file.

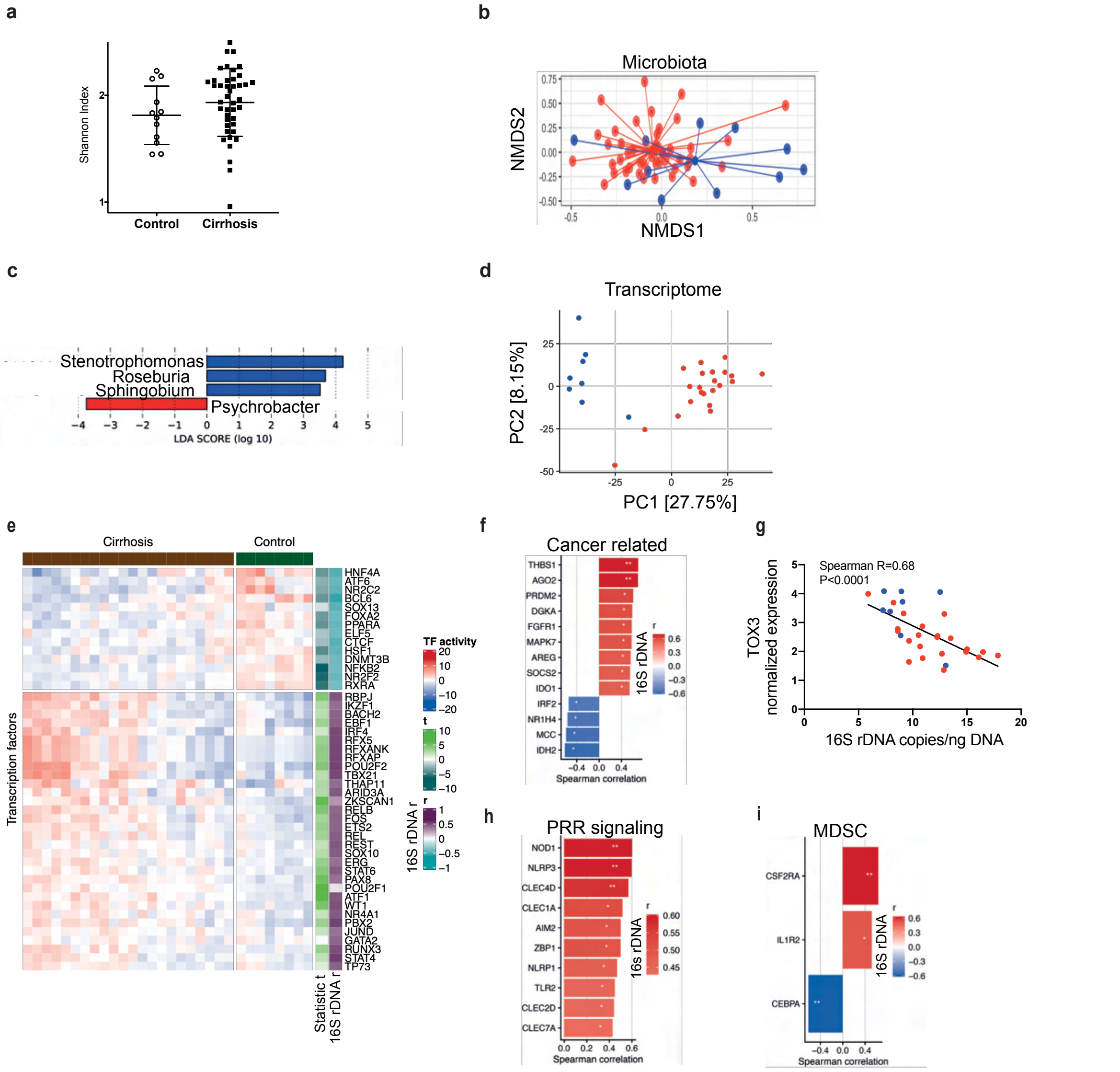

Supplementary Figure 9: Hepatic bacterial 16s rRNA gene abundance is increased in cirrhosis patients and shapes the hepatic transcriptomic landscape. (a) Microbiota diversity expressed as Shannon index based on 16s rRNA gene reads in liver tissue samples of control (n=12) and cirrhosis (n=43) patients. **(b)** NMDS ordination based on bray-Curtis dissimilarity index showing microbiota of cirrhosis and control patients. **(c)** Linear discriminant analysis (LDA) of effect size (LEfSe) between cirrhosis patients (n=43) and healthy controls (n=12). **(d)** PCoA graph of mRNA sequencing data comparing cirrhosis patients (n=22) and healthy controls (n=8). **(e)** Transcription factor (TF) activity inferred from gene expression data by interrogating the expression of respective transcriptional targets comparing cirrhosis patients (n=22) with healthy controls (n=8) and correlation of TF activities with 16S rRNA gene abundance. **(f)** Correlation of cancer-related gene expression with 16S rRNA gene abundance (Spearman correlation, n=30 pairs, two-tailed). **(g)** Strong inverse correlation of TF TOX expression and 16S rRNA gene abundance (Spearman, n=30 pairs, two-tailed, $p<0.0001$). **(h)** Correlation of pathogen recognition receptor and **(i)** MDSC-related gene expression with 16S rRNA gene abundance (Spearman, n=30 pairs, two-tailed). Data are presented as the mean ± standard error of the mean (SEM). Source data are provided as a Source data file.

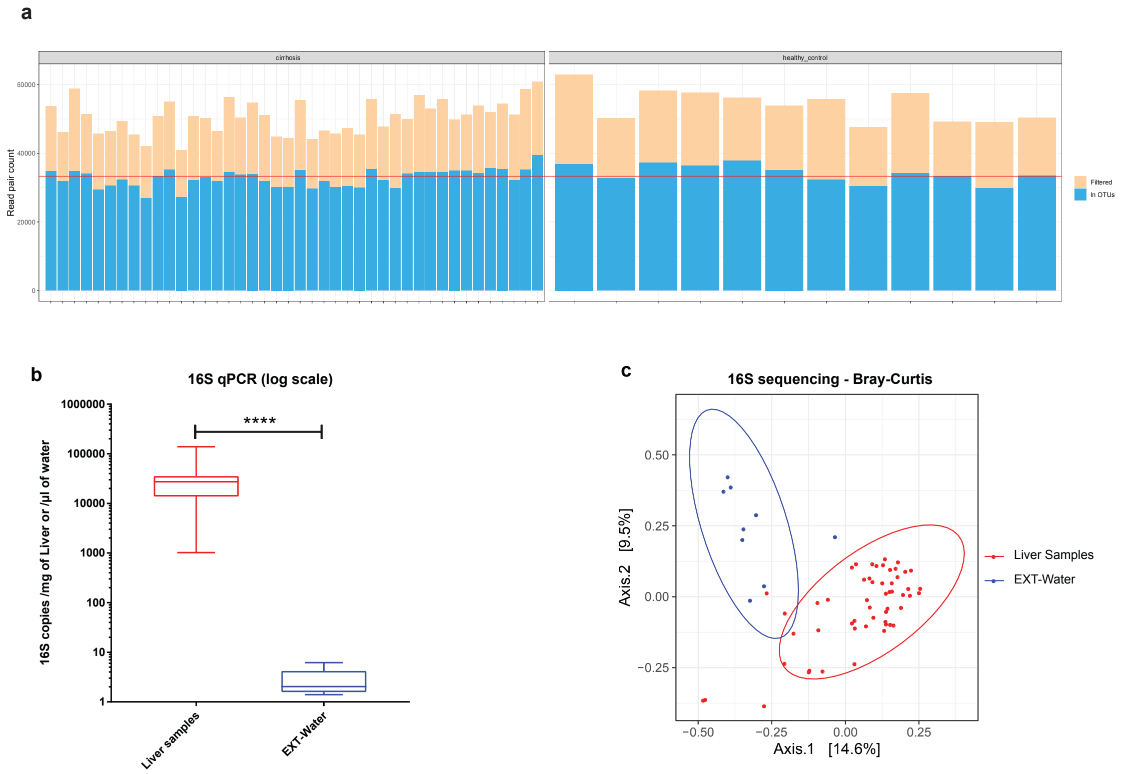

Supplementary Figure 10: Quality controls for 16S rRNA gene amplicon sequencing from tissue specimen.
(a) Total numbers of total sequence (merged paired reads) per liver sample (n=43 cirrhosis, n=12 control) (blue + orange), filtered from the data (orange) and clustered in Operational Taxonomic Units (OTUs, blue). (**b)** Boxplot of the quantity of bacterial DNA assessed by qPCR in liver samples (n=55) and negative controls (n=8, EXT-Water, two-tailed Mann-Whitney test, *p*=<0.0001), Liver samples: Min 1020, max= 139158, 25% percentile 13715, 75% percentile 35541, median 27310; EXT-water: Min 1.4, max = 6.18, 25% percentile = 1.576, 75% percentile 4.213, Median = 2.034 **(c)** Comparison of beta diversities by ordination analysis of the 16S rRNA gene amplicon sequencing data using Bray-Curtis dissimilarity distances in the liver samples (n=55) and negative control (n=9, EXT-Water). EXT-Water: molecular grade water added in an empty tube, extracted, amplified, and sequenced at the same time as the liver samples. Source data are provided as a Source data file.

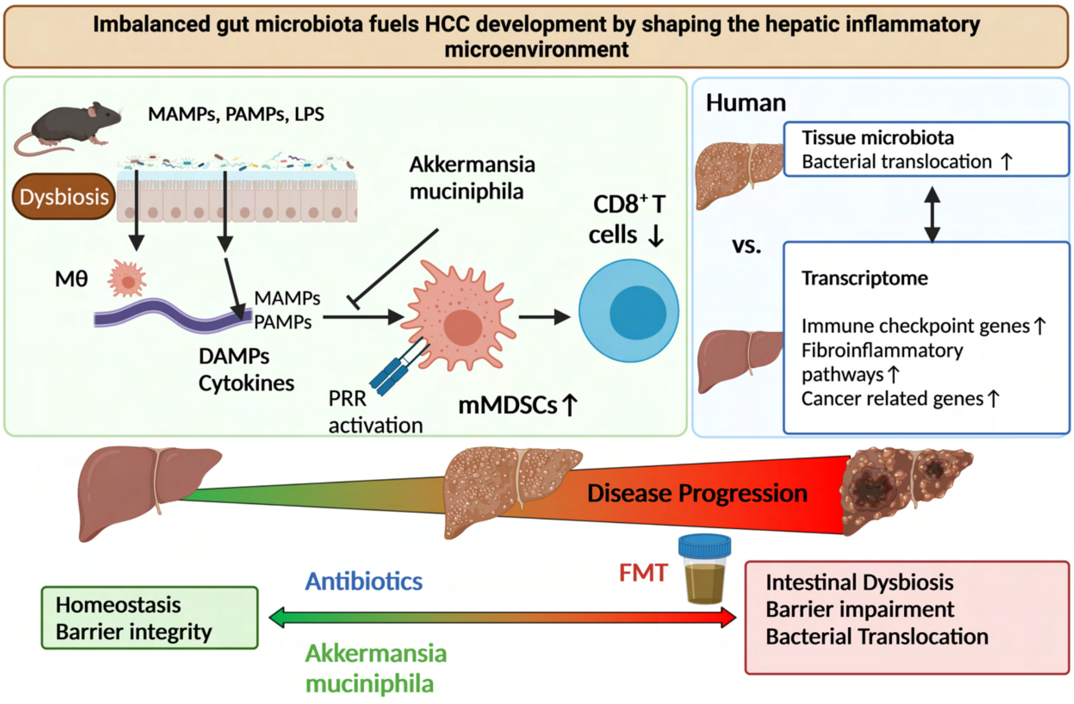

Supplementary Figure 11: Graphical abstract

Supplementary Table 1: Significantly differentially abundant OTUs before vs. after transfer of Akkermansia muciniphila using AldeX2.
Abbreviations: OTU, operational taxonomic unit; BH cor. *P* values, Expected Benjamini-Hochberg corrected *P* value of Welch's test.

Kingdom	Phylum	Class	Order	Family	Genus	effect	*BH Cor. P* value
**Bacteria**	Firmicutes	Clostridia	Clostridiales	Lachnospiraceae	Tyzzerella 3	4.628518289787241	0.007558569311205789
**Bacteria**	Firmicutes	Erysipelotrichia	Erysipelotrichales	Erysipelotrichaceae	Ambiguous_taxa	-3.9294936439919765	0.02988115059983605
**Bacteria**	Actinobacteria	Coriobacteriia	Coriobacteriales	Coriobacteriaceae	Ambiguous_taxa	-3.1818913681445533	0.022855510435574624
**Bacteria**	Proteobacteria	Gammaproteobacteria	Enterobacteriales	Enterobacteriaceae	Escherichia-Shigella	2.8604144392435833	0.0389360466772027
**Bacteria**	Proteobacteria	Betaproteobacteria	Burkholderiales	Alcaligenaceae	Turicimonas	2.3378550188282734	0.03364669552003225
**Bacteria**	Bacteroidetes	Bacteroidia	Bacteroidales	Rikenellaceae	Alistipes	3.724111413554155	0.012797108920769011
**Bacteria**	Bacteroidetes	Bacteroidia	Bacteroidales	Bacteroidales S24-7 group	Ambiguous_taxa	2.529821038839123	0.030873671547166272
**Bacteria**	Bacteroidetes	Bacteroidia	Bacteroidales	Bacteroidales S24-7 group	Ambiguous_taxa	3.00570673438616	0.01498660263825315
**Bacteria**	Bacteroidetes	Bacteroidia	Bacteroidales	Bacteroidaceae	Bacteroides	2.743480939765244	0.01630795975214222
**Bacteria**	Bacteroidetes	Bacteroidia	Bacteroidales	Bacteroidaceae	Bacteroides	3.795790657810244	0.008166480738850084
**Bacteria**	Bacteroidetes	Bacteroidia	Bacteroidales	Porphyromonadaceae	Parabacteroides	4.76884172143162	0.005191197245777214
**Bacteria**	Verrucomicrobia	Verrucomicrobiae	Verrucomicrobiales	Verrucomicrobiaceae	Akkermansia	-4.277096179243404	0.0223341141720361
**Bacteria**	Firmicutes	Clostridia	Clostridiales	Lachnospiraceae	Anaerostipes	3.266885446159116	0.013254168359999301

Supplementary Table 2: Characteristics of cirrhosis patients undergoing liver transplantation.

	**ALC** *n*=12	**HBV** *n*=7	**HCV** *n*=11	**Cholestatic liver disease** *n*=8	**Other** *n*=5
**Characteristics**					
Age at LTX (years)	49(41;55)	49(43;56)	45(42;58)	47(40;55)	34(30;42)
Females (%)	83	85	45	75	40
**Clinical liver status**					
Child Pugh Score (points)	10(7;11)	9(7;11)	7(7;10)	8(6;10)	10(8;11)
Meld Score (points)	18(13;22)	15(12;19)	12(9;19)	8(6;10)	18(16;29)
Esophageal varices (paquet)	2(1;3)	2(2;3)	2(1;2)	2(1;2)	2(1;2)
Variceal bleeding (%)	63	75	43	50	75

Ascites (%)	83	81	50	38	60
Hepatorenal Syndrome (%)	25	14	9	13	0
SBP (%)	17	0	9	0	0
Hepatic encephalopathy (%)	6	14	9	0	20
Time from first diagnosis to transplant (years)	1(1;3)	8(3;18)	3(6;16)	5(1;9)	2(0;6)
Edema (%)	67	50	43	17	20
**Liver-related blood parameters**					
Alanine aminotransferase (U/l)	23(16;37)	26(17;66)	71(31;131)	53(25;94)	43(33;65)
Aspartate aminotransferase (U/l)	22(15;37)	33(19;51)	52(25;108)	73(39;95)	57(42;65)
Gamma glutamyltransferase (U/l)	58(22;88)	47(14;77)	40(16;51)	201(145;356)	35(29;104)
Direct bilirubin (mg/dl)	3(2;12)	3(1;5)	2(1;3)	4(1;15)	4(3;24)
Alkaline phosphatase (U/l)	159(110;223)	148(97;258)	151(109;195)	601(352;1070)	224(149;664)
Platelet count (10^9/ml)	112(77;134)	65(52;90)	75(59;107)	167(134;276)	60(56;66)
Albumin (g/l)	26(3;30)	4(3;21)	3(3;34)	22(3;41)	33(10;36)
INR	1(1;2)	1(1;1)	1(1;1)	1(1;1)	2(1;2)
AFP (ng/ml)	3(2;7)	26(9;101)	17(5;35)	4(2;7)	12(3;12)
CRP (mg/l)	9(7;11)	5(0;17)	2(0;4)	9(4;22)	12(3;28)
Total protein (g/l)	66(40;70)	8(7;45)	64(7;76)	46(7;80)	49(6;73)
Creatinine (mg/dl)	1(1;1)	1(1;1)	1(1;1)	1(1;1)	1(1;1)
Sodium(mmol/l)	136(131;139)	137(132;143)	138(130;141)	139(135;142)	136(135;138)
Potassium (mmol/l)	4(3;4)	4(4;5)	4(4;4)	4(4;5)	4(4;4)
Calcium (mmol/l)	2(2;2)	2(2;2)	2(2;2)	2(2;3)	2(2;2)
Leucocytes (G/l)	7(6;8)	5(3;6)	4(3;7)	7(5;9)	5(4;6)
Erythrocytes (10^6/µl)	3(3;4)	4(3;4)	4(3;4)	4(4;4)	3(3;4)
Hemoglobin (g/dl)	11(10;13)	12(11;13)	12(11;13)	13(11;14)	11(10;13)
Hematocrit (%)	31(28;37)	35(31;39)	34(30;40)	39(33;40)	33(28;37)
**Liver Microbiota**					
16S copies/ng DNA	11(10;14)	12(9;12)	11(10;14)	9(8;11)	11(8;11)

All data are shown as median (IQR).

Supplementary Table 3: Correlation of bacterial abundance (order level) with clinical features.

Bacteria (Order)	Clinical feature	r	p-value	FDR
Lactobacillales	Bilirubin	-0.434	0.00327	0.0458
Lactobacillales	MELD	-0.4	0.00711	0.0995
Micrococcales	gGT	-0.391	0.00877	0.123
Pseudomonodales	Kreatinin	-0.386	0.00966	0.135
Betaproteobacteriales	Total Protein	0.32	0.0391	0.182
Corynebacteriales	Total Protein	-0.321	0.0382	0.182
Falvobacteriales	Total Protein	0.350	0.0233	0.182
Lactobacillales	INR	-0.367	0.0142	0.199

Spearman correlation, all p-values by two-tailed tests.

Supplementary Table 4: Correlation bacterial abundance (order level) with pathway activation scores. Spearman correlation.

Bacteria	Pathway	r	p-value	fdr
Clostridiales	MAPK	0.505	0.004	0.051
Clostridiales	EGFR	0.479	0.007	0.052
Clostridiales	TNFa	0.428	0.018	0.085
Corynebacteriales	PI3K	-0.488	0.006	0.087
Clostridiales	NFkB	0.396	0.030	0.105
Bacteroidales	TNFa	0.411	0.024	0.181
Bacteroidales	MAPK	0.405	0.026	0.181
Bacteroidales	EGFR	0.379	0.038	0.181
Pseudomonadales	Estrogen	-0.451	0.013	0.184
Bacteroidales	NFkB	0.352	0.056	0.196

Spearman correlation, all p-values by two-tailed tests.

Supplementary Table 5: Correlation of 16S rDNA copies/ngDNA with xcell Scores of different cell types.

Celltype	r	p-value	fdr
CD8+ T-cells	0.534	0.002	0.14
NKT	0.508	0.004	0.14
Th1 cells	-0.458	0.011	0.196
CD4+ Tcm	0.441	0.015	0.196
Tregs	0.419	0.021	0.204
MicroenvironmentScore	0.405	0.027	0.213
aDC	0.399	0.029	0.213
Fibroblasts	0.393	0.032	0.213
StromaScore	0.34	0.066	0.354
ImmuneScore	0.326	0.079	0.354
pDC	0.318	0.087	0.355
B-cells	0.315	0.09	0.355
Eosinophils	0.298	0.109	0.388
Epithelial cells	0.298	0.11	0.388
CD8+ Tcm	0.274	0.143	0.447
Hepatocytes	-0.268	0.152	0.447
cDC	0.268	0.153	0.447
DC	0.266	0.155	0.447
iDC	0.245	0.192	0.468
naive B-cells	0.243	0.196	0.468
Macrophages M2	-0.241	0.2	0.468
Tgd cells	-0.239	0.203	0.468
Basophils	0.232	0.217	0.469
Class-switched memory B-cells	0.216	0.253	0.502
CD8+ naive T-cells	0.182	0.335	0.612
pro B-cells	-0.164	0.388	0.666

Plasma cells	0.148	0.436	0.701
CD4+ memory T-cells	0.147	0.439	0.701
CD4+ Tem	-0.139	0.465	0.724
Monocytes	0.114	0.55	0.818
NK cells	0.099	0.603	0.86
Macrophages	-0.083	0.663	0.871
CD8+ Tem	0.069	0.716	0.873
Megakaryocytes	-0.066	0.727	0.873
Memory B-cells	0.061	0.75	0.873
CD4+ naive T-cells	0.056	0.77	0.873
Neutrophils	-0.045	0.814	0.873
CD4+ T-cells	-0.044	0.818	0.873
Platelets	0.042	0.827	0.873
Macrophages M1	-0.036	0.85	0.873
Th2 cells	0.034	0.86	0.873
Mast cells	-0.009	0.964	0.964

Spearman correlation, all p-values by two-tailed tests.

Supplemental Table 6: Antibodies and staining conditions

Target	Primary antibody		Secondary antibody	
	Antibody	Dilution	Antibody	Dilution
Leukocytes	Rat anti- CD45 [550994, BD bioscience, Heidelberg, Germany]	1:400	Rabbit anti-rat IgG, biotinylated [BA-4000-1.5, Vector Laboratories, Burlingame, CA, USA]	1:1000
Macrophages	Rat anti-F4/80 [MCA497, Bio-Rad, Feldkirchen, Germany]	1:50	Rabbit anti-rat IgG, biotinylated [BA-4000-1.5, Vector Laboratories, Burlingame, CA, USA]	1:1000
Proliferation	Rabbit anti-KI67 [ab16667, Abcam, Cambridge, UK]	1:1000	Goat anti-rabbit IgG, biotinylated [BA-1000-1.5, Vector Laboratories, Burlingame, CA, USA]	1:2000
CD8+ Lymphocytes	Rat anti-CD8	1:400	Cy3 goat anti-rat [A10522, Thermo Fisher Scientific, Waltham. MA, USA]	1:400
Cleaved Caspase 3	Rabbit anti-cleaved Caspase 3 [#9661, Cell signaling, Danvers, MA, USA]	1:1000	Goat anti-rabbit IgG, biotinylated [BA-1000-1.5, Vector Laboratories, Burlingame, CA, USA]	1:2000
Tight-Junctions	Rabbit anti-ZO-1 [ab96587, Abcam, Cambridge, UK]	1:200	Alexa-Fluor 488 goat anti-rabbit [A11088, Thermo Fisher Scientific, Waltham, MA, USA]	1:400

Macrophages/ Neutrophils	Rat anti-CD11b [55022, BD bioscience, Heidelberg, Germany]	1:200	Cy3 goat anti-rat [A10522, Thermo Fisher Scientific, Waltham, MA, USA]	1:400
Mucus	Rabbit anti-Muc2 [sc-158 15334, Santa Cruz Biotechnology, Dallas, TX, USA]	1:500	Alexa-Fluor 488 goat anti-rabbit [A11088, Thermo Fisher Scientific, Waltham, MA, USA]	1:200

Supplemental Table 7: Primer Sequences

Target gene symbol	Primer sequence (5'->3')	
	forward	reverse
Arg1	ACAAGACAGGGCTCCTTTCAG	GGCTTATGGTTACCCTCCCG
Caspase1	CCAAGCTTGAAAGACAAGCCC	ACTCCTTGTTTCTCTCCACGG
Ccl5	TCCTCATTGCTACTGCCCTC	GGCATCCTTGACCTGTGGAC
Col1a1	TGTGTGCGATGACGTGCAAT	GGGTCCCTCGACTCCTAC
Gapdh	TGTTGAAGTCACAGGAGACAACCT	AACCTGCCAAGTATGATGACATCA
Gli1	AACTCCACAGGCACACAGG	GCTCAGGCTTCTCCTCTCTC
Gli2	CCATTCATAAGCGGAGCAAG	CCAGGTCTTCCTTGAGATCG
Gli3	GCTCTTCAGCAAGTGGTTCC	CTGTCGGCTTAGGATCTGTTG
Ihh	CCGAACCTTCATCTTGGTG	ACAGATGGAATGCGTGTGAA
Il18	GACTCTTGCGTCAACTTCAAGG	CAGGCTGTCTTTTGTCAACGA
Il1b	CCTGAACTCAACTGTGAAATGCCAC	GGATGCTCTCATCTGGACAGCC
Il6	ACTTCACAAGTCGGAGGCTT	TGCAAGTGCATCATCGTTGT
Mcp1	GTGTTGGCTCAGCCAGATGC	GACACCTGCTGCTGGTGATCC
Nlrp3	CCACAAGATCGTGAGAAAACCC	CGGTCCTATGTGCTCGTCA
Nos2	GTTCTCAGCCCAACAATACAAGA	GTGGACGGGTCGATGTCAC
Ptc	ATGCTCCTTTCCTCCTGAAACC	TGAACTGGGCAGCTATGAAGTC
Shh	CTGGCCAGATGTTTTCTGGT	TAAAGGGGTCAGCTTTTTGG
Smo	GCCTGGTGCTTATTGTGG	GGTGGTTGCTCTTGATGG
Tgf-beta	GCTCGCTTTGTACAACAGCACC	GCGGTCCACCATTAGCACG
Tlr4	TTCAGAACTTCAGTGGCTGGATT	CCATGCCTTGTCTTCAATTGTTT
Tnfa	GCCGATTTGCTATCTCATACCAGG	CCCAGGTATATGGGCTCATACC
