# Supplementary material for: Imbalanced gut microbiota fuels hepatocellular carcinoma development by shaping the hepatic inflammatory microenvironment
Source: Nat Commun. 2022 Jul 8;13:3964. doi: 10.1038/s41467-022-31312-5 (PMC9270328; doi:10.1038/s41467-022-31312-5)
Supplement: Supplementary file 2 — Reporting Summary [file 41467_2022_31312_MOESM2_ESM.pdf]

nature research

Corresponding author(s): Christian Trautwein

Last updated by author(s): Jun 10, 2022

Reporting Summary

Nature Research wishes to improve the reproducibility of the work that we publish. This form provides structure for consistency and transparency in reporting. For further information on Nature Research policies, see our Editorial Policies and the Editorial Policy Checklist.

Statistics

For all statistical analyses, confirm that the following items are present in the figure legend, table legend, main text, or Methods section.

n/a	Confirmed	
☐	☒	The exact sample size (*n*) for each experimental group/condition, given as a discrete number and unit of measurement
☐	☒	A statement on whether measurements were taken from distinct samples or whether the same sample was measured repeatedly
☐	☒	The statistical test(s) used AND whether they are one- or two-sided<br>*Only common tests should be described solely by name; describe more complex techniques in the Methods section.*
☐	☒	A description of all covariates tested
☐	☒	A description of any assumptions or corrections, such as tests of normality and adjustment for multiple comparisons
☐	☒	A full description of the statistical parameters including central tendency (e.g. means) or other basic estimates (e.g. regression coefficient) AND variation (e.g. standard deviation) or associated estimates of uncertainty (e.g. confidence intervals)
☐	☒	For null hypothesis testing, the test statistic (e.g. *F*, *t*, *r*) with confidence intervals, effect sizes, degrees of freedom and *P* value noted<br>*Give P values as exact values whenever suitable.*
☒	☐	For Bayesian analysis, information on the choice of priors and Markov chain Monte Carlo settings
☒	☐	For hierarchical and complex designs, identification of the appropriate level for tests and full reporting of outcomes
☒	☐	Estimates of effect sizes (e.g. Cohen's *d*, Pearson's *r*), indicating how they were calculated

Our web collection on statistics for biologists contains articles on many of the points above.

Software and code

Policy information about availability of computer code

Data collection	BD FACSDiva 6 (BD bioscience), AxioVision 4.9 (Zeiss), Carl-Zeiss ZEN 2.6, ImageJ 1.52p (NIH, USA)
Data analysis	16S rRNA analysis was conducted based on previously described computational workflow (Galvez, et al 2017). The OTU absolute abundance table and mapping file were used for statistical analyses and data visualization in the R statistical programming environment (http://www.rproject.org) package phyloseq (McMurdie and Holmes, 2013). RNA Sequencing data were analysed using R as detailed in the methods section. Flow cytometry data were analysed using FlowJo (Ashland) version 9. Histological images were analysed with imageJ 1.52p (NIH, USA). of the cirrhosis cohort were performed using SPSS version 25. For graphic representation and statistical analysis R version 3.6, Rstudio and GraphPad Prism 8.0 were used.

For manuscripts utilizing custom algorithms or software that are central to the research but not yet described in published literature, software must be made available to editors and reviewers. We strongly encourage code deposition in a community repository (e.g. GitHub). See the Nature Research guidelines for submitting code & software for further information.

Data

Policy information about availability of data

All manuscripts must include a data availability statement. This statement should provide the following information, where applicable:

- Accession codes, unique identifiers, or web links for publicly available datasets
- A list of figures that have associated raw data
- A description of any restrictions on data availability

Data Availability

Raw sequence reads of 16s rRNA amplicon sequencing are available via BioProject databases:

- Murine data BioProject ID:PRJNA648423
- Human data BioProject ID: PRJNA842663

Raw sequence reads of bulk RNA sequencing of human samples are available via BioProject ID:PRJNA844027
Taxonomy assignment was performed using a curated Silva database v128 (Quast C, 2013). The remaining data are available within the Article, Supplementary Information or Source Data file.

The remaining data are available within the Article, Supplementary Information or Source Data file.

Field-specific reporting

Please select the one below that is the best fit for your research. If you are not sure, read the appropriate sections before making your selection.

[x] Life sciences [ ] Behavioural & social sciences [ ] Ecological, evolutionary & environmental sciences

For a reference copy of the document with all sections, see nature.com/documents/nr-reporting-summary-flat.pdf

Life sciences study design

All studies must disclose on these points even when the disclosure is negative.

Sample size	Patients included in our study were part of an observational trial and samples were taken during surgery. All patients were diagnosed according to established criteria. Sample size was determined by tissue availability and our own prior studies (Brol et al., Am J Physiol Gastrointest Liver Physiol. 2019, Schierwagen et al., Frontiers in Immunology 2020).<br>In the case of mouse studies, sample size was determined based on published as well as our own prior studies (Schneider et al., Hepatology 2015; Liao et al., Gut 2019)
Data exclusions	No data were excluded.
Replication	All data were successfully replicated and data from multiple experiments were pooled at least from two independent trials. All attempts at replication were successful.
Randomization	Upon birth, male mice were randomized to either no treatment, FMT or ABx groups and followed up until week 13. Experiments for these age progression experiments were run and analyzed in parallel. FMT or ABx was initiated in the respective groups at 7-9 weeks of age and continued until week 13.
Blinding	Library preparation for 16s rRNA amplicon sequencing, preliminary analysis of sequencing and flow cytometry data were performed in a blinded fashion. Investigators were blinded during manual cell counting and imaging analysis.<br>Moreover, Investigators were blinded regarding the genotypes during in-vivo experiments. Processing of serum and tissue samples were performed based on mouse ID without genotype/group information.<br>Investigators were not blind to treatment groups for experiments involving oral gavage (Akkermansia transfer) or antibiotic treatment since the treatment groups had to be clearly identified throughout the study to prevent cross contamination between groups.

Reporting for specific materials, systems and methods

We require information from authors about some types of materials, experimental systems and methods used in many studies. Here, indicate whether each material, system or method listed is relevant to your study. If you are not sure if a list item applies to your research, read the appropriate section before selecting a response.

Materials & experimental systems

n/a	Involved in the study	
[ ]	[x]	Antibodies
[x]	[ ]	Eukaryotic cell lines
[x]	[ ]	Palaeontology and archaeology
[ ]	[x]	Animals and other organisms
[ ]	[x]	Human research participants
[x]	[ ]	Clinical data
[x]	[ ]	Dual use research of concern

Methods

n/a	Involved in the study	
[x]	[ ]	ChIP-seq
[ ]	[x]	Flow cytometry
[x]	[ ]	MRI-based neuroimaging

Antibodies

Antibodies used

IHC and IF stainings:
- Leukocytes: Rat anti- CD45 [BD bioscience, Heidelberg, Germany, LOT #8193659], Dilution 1:400; Secondary Rabbit anti-rat IgG [BA-4000-1.5, Vector Laboratories, Burlingame, CA, USA, LOT #ZF0422], Dilution 1:1000
- Macrophages: Rat anti-F4/80 [Bio-Rad, Feldkirchen, Germany, LOT #1702], Dilution 1:50; Secondary Rabbit anti-rat IgG [BA-4000-1.5, Vector Laboratories, Burlingame, CA, USA, LOT #ZF0422], Dilution 1:1000
- Tight-Junctions: Rabbit anti-ZO-1[Abcam, Cambridge, UK, Ab96587, LOT #QA213066], Dilution 1:200; Secondary Alexa-Fluor 488 IgG Goat anti-rabbit [A11088, Thermo Fisher Scientific,Waltham, MA, USA, LOT #1885240], Dilution 1:400
- MDSCs: Rat anti-CD11b [BD bioscience, Heidelberg, Germany, LOT #7152719], Dilution 1:200; Secondary Cy3 IgG Goat anti-rat [A10522, Thermo Fisher Scientific,Waltham, MA, USA, LOT #1842204], Dilution 1:400
- Proliferation: Rabbit anti-KI67 [ab16667, Abcam, Cambridge, UK], Dilution 1:1000; Secondary biotinylated Goat anti-rabbit IgG [BA-1000-1.5, Vector Laboratories, Burlingame, CA, USA], Dilution 1:2000
- CD8+ Lymphocytes: Rat anti-CD8, FITC conjugated IgG [#14-0081-82, Thermo Fisher Scientific,Waltham, MA, USA], Dilution 1:400
- Apoptosis: Rabbit anti-cleaved Caspase 3 [#9661, Cell signaling, Danvers, MA, USA], Dilution 1:1000; Secondary Goat anti-rabbit IgG, biotinylated [BA-1000-1.5, Vector Laboratories, Burlingame, CA, USA], Dilution 1:2000
- Mucus: Rabbit anti-Muc2 [sc-158 15334, Santa Cruz Biotechnology, Dallas, TX, USA], Dilution 1:500, Secondary goat anti-rabbit IgG, Alexa-Fluor 488 [A11088, Thermo Fisher Scientific, Waltham, MA, USA], Dilution 1:200

Western Blot:
- β-actin (A2066, Sigma-Aldrich, St. Louis, MO, USA), Dilution 1:1000
- Occludin (71-1500, Thermo Fisher Scientific, Waltham, MA, USA 71-1500), Dilution 1:1000
- p-JNK/p-SAPK (#9251S, Cell signaling, Danvers, MA, USA), Dilution 1:1000
- JNK/SAPK (#9252S, Cell signaling, Danvers, MA, USA), Dilution 1:1000
- GAPDH (AHP1628, Bio-Rad, Hercules CA, USA), Dilution 1:1000
- Secondary HRP Anti-rabbit [Cell signaling, Danvers, MA, USA, #7074, LOT #70749], Dilution 1:2000

FACS:
Fluorochrome-conjugated antibodies either for myeloid cells FITC Rat anti-Mouse Ly-6G (561105; BD bioscience, Heidelberg, Germany), CD11b Monoclonal Antibody (M1/70), PE (12-0112-82, Thermo Fisher Scientific, Waltham, MA, USA), APC anti-mouse CD11c (117310, Biolegend, San Diego, CA, USA), F4/80 Monoclonal Antibody (BM8), PE-Cyanine7 (25-4801-82, Thermo Fisher Scientific, Waltham, MA, USA), PerCP-Cy™5.5 Rat Anti-Mouse Ly-6G and Ly-6C (552093, BD bioscience, Heidelberg, Germany), APC-Cy™7 Rat Anti-Mouse CD45 (557659, BD bioscience, Heidelberg, Germany) (1:200) or lymphocytes CD3e Monoclonal Antibody (145-2C11), APC (17-0031-83, Thermo Fisher Scientific, Waltham, MA, USA), CD4 Monoclonal Antibody (GK1.5), PE (12-0041-83, Thermo Fisher Scientific, Waltham, MA, USA) CD8a Monoclonal Antibody (53-6.7), FITC (11-0081-85, Thermo Fisher Scientific, Waltham, MA, USA), PerCP-Cy™5.5 Rat Anti-Mouse CD19 (551001, BD bioscience, Heidelberg, Germany), NK1.1 Monoclonal Antibody (PK136), PE-Cyanine7 (25-5941-82, Thermo Fisher Scientific, Waltham, MA, USA), APC-Cy™7 Rat Anti-Mouse CD45 (557659, BD bioscience, Heidelberg, Germany) (1:200) were used.

Validation

Primary antibodies were chosen based on prior publications and on the manufacturer's validation reports. Optimal staining conditions and dilutions were determined.
All FACS antibodies were selected based on the manufacturer's validation reports. Antibodies were tested using single stainings of various tissue suspensions as well as flow cytometry compensation beads.

FITC Rat anti-Mouse Ly-6G (561105; Lot number: 8116599 (https://regdocs.bd.com/regdocs/qcSearchResults) ,BD bioscience, Heidelberg, Germany)
CD11b Monoclonal Antibody (M1/70), PE (12-0112-82, Lot number: 2101306 (https://www.thermofisher.com/document-connect/document-connect.html?url=https%3A%2F%2Fassets.thermofisher.com%2FTFS-Assets%2FLSG%2Fcertificate%2FCertificates-of-Analysis%2F12011282_2101306.PDF&title=TG90ICMmbmJzcDsyMTAxMzA2), Thermo Fisher Scientific, Waltham, MA, USA)
APC anti-mouse CD11c (117310, Lot Number: B331091 (https://www.biolegend.com/Default.aspx?Id=18921), Biolegend, San Diego, CA, USA)
F4/80 Monoclonal Antibody (BM8), PE-Cyanine7 (25-4801-82, Lot number: 2279168 (https://www.thermofisher.com/document-connect/document-connect.html?url=https%3A%2F%2Fassets.thermofisher.com%2FTFS-Assets%2FLSG%2Fcertificate%2FCertificates-of-Analysis%2F25480182_2279168.PDF&title=TG90ICMmbmJzcDsyMjc5MTY4), Thermo Fisher Scientific, Waltham, MA, USA)
PerCP-Cy™5.5 Rat Anti-Mouse Ly-6G and Ly-6C (552093, Lot number: 9273679 (https://regdocs.bd.com/regdocs/qcSearchResults), BD bioscience, Heidelberg, Germany)
APC-Cy™7 Rat Anti-Mouse CD45 (557659, Lot number: 1334944 (https://regdocs.bd.com/regdocs/qcSearchResults), BD bioscience, Heidelberg, Germany)
CD3e Monoclonal Antibody (145-2C11), APC (17-0031-83, Lot number: 2324878 (https://www.thermofisher.com/document-connect/document-connect.html?url=https%3A%2F%2Fassets.thermofisher.com%2FTFS-Assets%2FLSG%2Fcertificate%2FCertificates-of-Analysis%2F17003183_2324878.PDF&title=TG90ICMmbmJzcDsyMzI0ODc4), Thermo Fisher Scientific, Waltham, MA, USA)
CD4 Monoclonal Antibody (GK1.5), PE (12-0041-83, Lot number: 1934791 (https://www.thermofisher.com/antibody/product/CD4-Antibody-clone-GK1-5-Monoclonal/12-0041-83), Thermo Fisher Scientific, Waltham, MA, USA)
CD8a Monoclonal Antibody (53-6.7), FITC (11-0081-85, Lot number: 2002714 (https://www.thermofisher.com/document-connect/document-connect.html?url=https%3A%2F%2Fassets.thermofisher.com%2FTFS-Assets%2FLSG%2Fcertificate%2FCertificates-of-Analysis%2F11008185_2002714.PDF&title=TG90ICMmbmJzcDsyMDAyNzE0), Thermo Fisher Scientific, Waltham, MA, USA)
PerCP-Cy™5.5 Rat Anti-Mouse CD19 (551001, Lot number: 3157934 (https://regdocs.bd.com/regdocs/qcSearchResults), BD bioscience, Heidelberg, Germany)

	NK1.1 Monoclonal Antibody (PK136), PE-Cyanine7 (25-5941-82, Lot number: 2103491 (https://www.thermofisher.com/document-connect/document-connect.html?url=https%3A%2F%2Fassets.thermofisher.com%2FTFS-Assets%2FLSG%2Fcertificate%2FCertificates-of-Analysis%2F25594182_2103491.PDF&title=TG90ICMmbmJzcDsyMTAzNDkx), Thermo Fisher Scientific, Waltham, MA, USA)

Animals and other organisms

Policy information about studies involving animals; ARRIVE guidelines recommended for reporting animal research

Laboratory animals	Male Alb-cre-NEMOΔhepa, Alb-cre-NEMOfl/fl referred to as WT, Alb-cre-NEMOΔhepa/Nlrp6-/- and Alb-cre-NEMOΔhepa/Tlr4-/- of the C57Bl6 background were bred and housed in the central animal facility of the University hospital RWTH Aachen. All mice were housed in the individually ventilated cages with access to a standard chow diet and drinking water ad libitum. For all experiments mice were used at 7-9 weeks of age and male gender. All mice were housed at a temperature of 21°-23°C with relative humidity of 35%-65% and 12h light/dark cycle.
Wild animals	The study did not include wild animals.
Field-collected samples	The study did not include field-collected samples.
Ethics oversight	All animal experiments were approved by the appropriate German authorities (LANUV. North Rhine-Westphalia. (#AZ84-02.04.2013.A184(C.T.), (#AZ84-02.04.2013.A260(C.T.), #AZ84-02.04.2017.A327 (C.T.), #AZ84-03.04.2013.A240 (C.T.)) All mice were treated in accordance to the criteria of the German administrative panels on laboratory animal care as outlined in the “Guide for the Care and Use of Laboratory Animals” prepared by the National Academy of Sciences and published by the National Institutes of Health (NIH publication 86-23 revised 1985).

Note that full information on the approval of the study protocol must also be provided in the manuscript.

Human research participants

Policy information about studies involving human research participants

Population characteristics	Patients included in our study (n= 14 males, n= 29 females, mean age = 45) were part of an observational trial and samples were collected for the purpose of our study. Human cirrhosis liver tissue specimen were taken from patients that underwent liver transplantation between 1999 and 2005 at the University Hospital Bonn (Table S1). Healthy surgical tissue specimen were obtained from patients who underwent clinically indicated liver resection at University Hospital Bonn or University Hospital rechts der Isar of the Technical University Munich.
Recruitment	Patients were recruited at admission to the University Hospital Bonn or University Hospital rechts der Isar of the Technical University Munich. Most eligible patients agreed to participation. As a consequence, self-selection bias is low.
Ethics oversight	The human ethics committee of the University of Bonn (029/13) approved the study. All patients gave written informed consent to use excess biopsy material for research purposes. The study of these pseudonymized tissue specimen has been approved by the local ethics committee RWTH Aachen University (EK 196/19).

Note that full information on the approval of the study protocol must also be provided in the manuscript.

Flow Cytometry

Plots

Confirm that:

- [x] The axis labels state the marker and fluorochrome used (e.g. CD4-FITC).
- [x] The axis scales are clearly visible. Include numbers along axes only for bottom left plot of group (a 'group' is an analysis of identical markers).
- [x] All plots are contour plots with outliers or pseudocolor plots.
- [x] A numerical value for number of cells or percentage (with statistics) is provided.

Methodology

Sample preparation	Same amounts of livers were digested by collagenase type IV for 1 hour at 37 °C (Worthington Biochemical Corporation, Lakewood, NJ, USA) and intrahepatic immune cells were isolated by multiple differential centrifugation steps. Cell isolates were incubated with blocking buffer for 30 min to block the unspecific binding sites of cell surface, then divided into 2 subgroups and stained with fluorochrome-conjugated antibodies either for myeloid cells Ly6G, CD11b, CD11c, F4/80, Gr1.1 and CD45 (1:200) or lymphocytes CD3, CD4, CD8, CD19, NK1.1 and CD45 (1:200). All flow cytometry antibodies were purchased from ebioscience (Frankfurt, Germany).
Instrument	Flow cytometry measurements were performed on a FACS Fortessa or FACS Canto instrument (BD, bioscience, Heidelberg, Germany).
Software	BD FACSDiva Software (BD Bioscience), FlowJo (Ashland) version 9

Cell population abundance	Cell population abundances were analyzed using FlowJo software version 9 and reported in figures. This study did not involve any cell-sorting experiments.
Gating strategy	We used standard gating strategies:<br><br>Myeloid cells:<br>1. Gating on the typical leukocyte population based on FSC-SSC signals<br>2. Single cells were selected using FSC-W, FSC-A<br>Cell populations were identified based on the expression of specific surface markers:<br>1. Living Leukocytes: CD45 expression, negative for Hoechst<br>2. Ly6G+ CD11b+ population (GMDCs): CD45+/CD11b+/Ly6G+<br>3. mMDSC: CD45+/Ly6G-/CD11b+/F4/80low Gr-1hi<br>4. KupfferCells: CD45+/Ly6G-/CD11b+/F4/80high<br><br>Lymphoid cells:<br>1. Gating on the typical leukocyte population based on FSC-SSC signals<br>2. Single cells were selected using FSC-W, FSC-A<br>Cell populations were identified based on the expression of specific surface markers:<br>1. Living Leukocytes: CD45 expression, negative for Hoechst<br>2. CD3+ CD8+ T-cells<br>3. CD+CD4+ T-cells<br>4. CD3+ Nk1.1+ Nk-cells<br>5. CD3-Nk1.1+ NK-cells<br>6. CD3- CD19+ B-cells<br><br>Gating strategy is described in Supplementary Figure 4.

☒ Tick this box to confirm that a figure exemplifying the gating strategy is provided in the Supplementary Information.
